# Prediction and immune landscape study of potentially key autophagy-related biomarkers in preeclampsia with gestational diabetes mellitus

**DOI:** 10.3389/fimmu.2025.1571795

**Published:** 2025-07-02

**Authors:** Qin Wang, Xiaoqi Li, Wen Ye, Lin Lin, Kejun Ye, Mengjia Peng

**Affiliations:** ^1^ Department of Geriatric Integrative, Second Affiliated Hospital of Xinjiang Medical University, Urumqi, Xinjiang, China; ^2^ The Second Affiliated Hospital of Xinjiang Medical University, The Second Clinical Medical College, Xinjiang Medical University, Urumqi, Xinjiang, China; ^3^ Nephrology Department, Second Affiliated Hospital of Xinjiang Medical University, Xinjiang, Urumqi, China; ^4^ Wenzhou Key Laboratory for the Diagnosis and Prevention of Diabetic Complications, Department of Gynecology and Obstetrics, The Third Affiliated Hospital of Wenzhou Medical University (Ruian People's Hospital), Rui’an, Zhejiang, China

**Keywords:** preeclampsia, gestational diabetes mellitus, autophagy, gene expression, omnibus, single-cell RNA

## Abstract

**Introduction:**

Gestational diabetes mellitus (GDM) and preeclampsia are prevalent pregnancy complications that threaten maternal and infant health while imposing substantial socioeconomic burdens. Although several interventions exist, shortcomings in individualized treatment and other limitations necessitate urgent in-depth research. This study aimed to examine alterations in autophagy-related gene expression in preeclampsia combined with GDM.

**Methods:**

We conducted bioinformatics analyses including gene expression profiling, weighted gene co-expression network analysis (WGCNA), gene ontology (GO) and KEGG enrichment analyses, machine learning modeling, immune infiltration analyses, and single-cell RNA sequencing. Differentially expressed autophagy-related genes linked to preeclampsia with GDM were identified. Expression levels of four key genes were validated in placental samples using reverse transcription quantitative polymerase chain reaction (RT-qPCR).

**Results:**

Our findings identified potential biomarkers and molecular mechanisms underlying preeclampsia with GDM. Single-cell analysis corroborated these results, revealing distinct autophagy-related gene signatures and enhancing understanding of the pathophysiology.

**Discussion:**

This study elucidates molecular mechanisms connecting GDM and preeclampsia, identifies novel biomarkers and therapeutic targets, and provides a valuable reference for future research and clinical applications. The integration of multi-omics approaches advances precision medicine strategies for these comorbid conditions.

## 1 Introduction

Preeclampsia (PE) and gestational diabetes mellitus (GDM) represent two major pregnancy complications that have the potential to affect maternal and foetal health. PE affects 2-8% of pregnancies globally, whereas GDM occurs in approximately 1.8-20.3% of pregnancies (1, 2). These conditions pose an immediate risk to the mother and foetus, and have long-term health consequences (3, 4). PE is a significant pregnancy complication characterised by high blood pressure and proteinuria after 20 weeks of gestation (5). GDM is characterised by glucose intolerance that occurs or is diagnosed for the first time during pregnancy, leading to hyperglycaemia and associated metabolic disorders (6). The potentially severe consequences of these disorders underscore the importance of identifying reliable biomarkers for early diagnosis and intervention.

Emerging evidence suggests that PE and GDM share common pathophysiological mechanisms, including endothelial dysfunction, inflammation, and metabolic dysregulation (7). GDM in late pregnancy increases the risk of developing PE, and patients with PE tend to have features of GDM, suggesting that the underlying biological pathways may overlap (8, 9). A number of large-scale cohort studies conducted among different populations have confirmed this association. For instance, a Latin American and Caribbean cohort demonstrated that GDM significantly elevates PE risk (RR: 1.93; 95% CI: 1.66–2.25) (10). Similarly, Swedish and Chinese cohorts revealed that GDM increases the likelihood of severe PE (Sweden: OR 2.29, 95% CI 1.88–2.80; China: OR 2.13, 95% CI 1.58–2.87) (11).

Autophagy, a cellular self-degradation process that supplies degradation products, is crucial for cellular homeostasis and linked to the pathogenesis of PE and GDM (12). In PE, abnormal autophagy can lead to an increased stress response and apoptosis of placental cells, resulting in placental dysfunction and impaired foetal growth and development (13). Autophagy may influence the onset and development of GDM by regulating the stress response and metabolic state of placental cells (13). Although extensive research has been conducted, the precise function of autophagy in PE and GDM remains unclear, necessitating additional studies to clarify its mechanism of action and therapeutic potential.

Previous studies have emphasised the significance of autophagy and immune cell infiltration in PE and GDM. The infiltration of immune cells into the placenta contributes significantly to the progression of these diseases (14–16). Autophagy regulates immune responses and inflammation, which are key components of the pathophysiology of PE and GDM (17). Interactions between autophagy-related genes (ARGs) and immune cell infiltration in these diseases remain underexplored and require comprehensive research.

This study used bioinformatics to identify differentially expressed autophagy-related genes (DE-AGs) in PE with GDM. We conducted differential expression and weighted gene co-expression network analysis (WGCNA) to identify DE-AGs in conjunction with autophagy-associated genes. We examined the biological functions and pathways of these DE-AGs using functional enrichment analysis, constructed protein-protein interaction (PPI) networks, and identified key genes using various machine learning techniques. Receiver operating characteristic (ROC) curves were used to assess the diagnostic potential of DE-AGs, and immune cell infiltration was evaluated to understand their immune efficacy. Finally, single-cell RNA sequencing data were analysed to determine the distribution of DE-AGs and different cell types in PE and GDM placental tissues. Our study comprehensively analysed the molecular mechanisms of PE complicating GDM and highlighted the roles of ARGs in these disorders. The identification of DE-AGs and their associated pathways provides potential biomarkers for early diagnosis and identification of therapeutic targets in PE and GDM.

## 2 Materials and methods

### 2.1 Data gathering and preparation

Gene expression profiles related to GDM and PE were obtained from the NCBI Gene Expression Omnibus (GEO) database (https://www.ncbi.nlm.nih.gov/geo/). Using the R package ‘GEOquery’ (v2.64.2) (18), data related to ‘preeclampsia’ and ‘gestational diabetes mellitus’ were retrieved from the GEO database. Five datasets were obtained from the GEO database: GSE103552, GSE75010, GSE24129, GSE154414, and GSE30186. The GSE75010 dataset comprises 80 patients with PE and 77 controls, the GSE24129 dataset contains eight patients with PE and eight control cases, the GSE30186 dataset contains six patients with PE and six control cases, the GSE154414 dataset contains four patients with GDM and four control cases, and the GSE103552 dataset includes ten patients with GDM and eight controls, and the GSE173193 dataset includes two placenta samples from PE, GDM and the control group respectively. We performed preprocessing on each dataset, employing the “leave-one-out” method to retain only the first occurrence of duplicate gene names in each dataset, the gene expression levels for all genes in each dataset were log-transformed to ensure that the gene expression values within each dataset had the same distribution. Next, we removed the batch effects between GSE75010 and GSE24129 using the normalizeBetweenArrays function from the “limma” (v3.52.2) package, enabling comparability of expression levels between the two datasets, and subsequently merged GSE75010 and GSE24129. Principal component analysis (PCA) was conducted on the normalised dataset, and box plots along with PCA plots were created using the ‘ggplot2’ R package (v3.3.6) (19) to visualise sample distribution and clustering.

### 2.2 Identification of differentially expressed genes

DEGs were identified by extracting samples from the GSE103552 and Merged_Dataset_GSE75010_GSE24129 datasets and conducting differential analysis using the R package ‘limma’(v3.60.4) (20). To ensure higher sensitivity in detecting differentially expressed genes, we established a more permissive fold change threshold to capture a broader range of potential variations.DEGs were identified in the two datasets using the criteria |log2 fold change (log2 FC)| > 0 and *p* < 0.05 (21, 22), followed by de-duplication of the results (23). Volcano maps were created with the R package ‘ggplot2’, while heat maps utilised the R package ‘ComplexHeatmap’ (v2.13.1) (24).

### 2.3 WGCNA

The raw gene expression data were preprocessed using the R package ‘WGCNA’ (v1.72-5) (25), and The distances between samples were calculated using the dist function, with the default metric being Euclidean distance. Subsequently, the pickSoftThreshold function was used to select the optimal soft threshold. Dynamic modules were identified using the cutreeDynamic function, with each module containing at least 50 genes (26). A dynamic dendrogram was drawn using the plotDendroAndColors function to show the associations and differences between different modules.

Topological Overlap Matrix (TOM) was calculated by the TOMsimilarity function to quantify gene co-expression similarity. Module eigengenes (MEs) were extracted for Pearson correlation analysis with clinical traits. Statistical significance was evaluated using Student’s asymptotic P-value (corPvalueStudent function), and results were visualized through a labeledHeatmap displaying correlation coefficients and P-values.

Based on the visualization results of the module clustering, we defined a cutting height: MEDissThres. Subsequently, by calling the mergeCloseModules function, we merged similar gene modules based on this cutting height, producing merged module colors and new module eigengenes (MEs). This simplification of the module structure enhances the biological significance of the analysis and facilitates subsequent functional enrichment and network analysis.

Modules significantly associated with preeclampsia (PE) were prioritized based on P-value ranking. Genes within PE-related modules were extracted for subsequent functional enrichment and regulatory network analyses.

### 2.4 Screening of ARGs

ARGs were sourced from four complementary databases: GeneCards (https://www.genecards.org/): A comprehensive repository integrating gene annotations from >150 biomedical resources; Human Autophagy Database (http://www.autophagy.lu/): A manually curated knowledgebase specializing in autophagy pathways and regulators; HAMdb (http://hamdb.scbdd.com/home/index/): A disease-focused platform linking autophagy genes to pathological mechanisms; MSigDB (https://www.gsea-msigdb.org/) (version: MSigDB 2023.2.Hs): A functional genomics resource providing hallmark gene sets for pathway enrichment. These genes were then intersected with DEGs and WGCNA modules and analysed to identify the DE-AGs in PE with GDM. Finally, the genes were visualized using the R package ‘VennDiagram’(v1.7.3).

### 2.5 Enrichment analysis of DE-AGs was conducted using Gene Ontology and the Kyoto Encyclopedia of Genes and Genomes

We performed GO enrichment analysis on the DE-AGs in *Homo sapiens*, systematically evaluating three functional categories: biological processes (BP), cellular components (CC), and molecular functions (MF). KEGG pathway analysis was concurrently conducted (27–29). Gene identifiers were standardized using the R package ‘org.Hs.eg.db’(v3.19.1), followed by functional enrichment analysis with ‘clusterProfiler’ (v4.12.6) (30). To quantify directional enrichment patterns, z-scores were calculated for each term using ‘GOplot’ (v1.0.2) (31), enabling quantitative assessment of biological pathway activation states. Terms with p < 0.05 and false discovery rate (FDR) < 0.2 were considered statistically significant (32). Results were filtered for both statistical significance and biological relevance, with final visualizations were generated.

### 2.6 PPI network

We utilized the STRING database (https://string-db.org/)(version:12.0) (33) to analyze protein–protein interactions among DE-AGs. The combined interaction confidence score (joint score) greater than 0.4 was selected as the medium confidence interaction threshold, and the interaction node data from STRING were imported into Cytoscape (v3.9.1) for PPI network analysis (34). Hub genes were systematically identified through the CytoHubba plugin by applying four complementary algorithms: Maximum Clique Centrality (MCC), Degree, Edge Percolated Component (EPC), and Density of Maximum Neighborhood Component (DMNC). The top 15 genes from each algorithm were cross-compared, and consensus hub genes were defined as those overlapping across all four methods. This integrative approach was visualized through a Venn diagram, highlighting genes consistently prioritized by multiple centrality metrics (35).

### 2.7 Identification of PE with GDM-related DE-AGs using machine learning

This study employed three machine learning models: least absolute shrinkage and selection operator (LASSO), support vector machine (SVM), and random forest (RF). The R package ‘DALEX’ (v2.4.3) was used to interpret these models and visualize residual distributions and feature significance. Hyperparameter optimization was systematically performed using the R package ‘caret’ (v6.0-94) through grid search across predefined parameter spaces. All models were evaluated via 10-fold cross-validation, with final parameters retained after validation.

Subsequently, the R package ‘pROC’ (36) was utilized to plot the area under the receiver operating characteristic (ROC) curve (AUC). Feature screening was then performed using LASSO, RF, and SVM methods. The intersection of features derived from these complementary algorithms was prioritized to mitigate model-specific biases. This integrative approach enhanced biomarker discovery reliability, as consensus genes were more likely to reflect biologically stable signatures in gestational diabetes mellitus (GDM) pathogenesis.

For LASSO analysis, we employed the R package ‘glmnet’ (v4.1.7) to screen coefficients. This involved analyzing cleaned data, extracting lambda values, likelihood values, L1 regularization values, and classification error rates. The results were visualized as described previously (37).

The SVM-based recursive feature elimination (SVM-RFE) (38) technique was implemented using the R package ‘e1071’ (v1.7-13) (39, 40). By incorporating a feature ranking process into the outer layer of cross-validation (41), we achieved an unbiased estimate of the generalization error.

In the RF algorithm, gene importance rankings were obtained using the average reduction in the Gini index as the indicator (42). The intersection of results from LASSO, SVM-RFE, and RF identified PE with GDM-related DE-AGs. These consensus genes were visualized using UpSet plots to demonstrate multi-algorithm superiority over single-method outputs. These consensus genes were visualized using UpSet plots to demonstrate the advantages of multiple algorithms over single-method outputs.

We employed the Spearman correlation method to evaluate relationships between four DE-AGs. Correlation heatmaps generated with the R package ‘corrplot’ (v0.92) illustrated gene associations and interactions. The non-parametric Spearman approach was chosen instead of Pearson correlation to account for potential nonlinear relationships and reduce sensitivity to expression value outliers. This strategy was critical for identifying robust co-expression patterns in heterogeneous clinical samples.

### 2.8 Examination of differential expression PE with GDM-related DE-AGs

We investigated the differences in the expression of PE with GDM-related DE-AGs between the experimental and control groups. Using Shapiro-Wilk tests for normality assessment (α=0.05) and F-tests for variance homogeneity, we selected appropriate statistical comparisons: independent t-tests for parametric data with equal variance (p>0.05) and Welch’s t-tests for unequal variance (p<0.05). Integrated visualizations combining scatter plots (showing individual data points), box plots (depicting quartiles), and violin plots (illustrating probability density) to comprehensively present distribution characteristics. Statistical significance thresholds were maintained as: ns p≥0.05; *p<0.05; **p<0.01; ***p<0.001, with detailed annotation in figure captions.

### 2.9 ROC analysis of PE with GDM-related DE-AGs

The ROC curves for the GSE103552, Merged_Dataset_GSE75010_GSE24129, GSE154414, and GSE30186 datasets were analysed using the R package ‘pROC’ (V1.18.0) to evaluate sensitivity and specificity. The accuracy of genes for diagnosing PE with GDM was assessed by predicting ROC-related information at specific cutoff values, quantified as the AUC. Genes with an AUC > 0.6 were visualized (43).

### 2.10 Exploration of the biological functions and signalling pathways of PE with GDM-related DE-AGs

We used the R package ‘clusterProfiler’(v4.12.6) to conduct gene set enrichment analysis (GSEA) (44) to identify pathways significantly linked to PE with GDM-related DE-AGs. Species: *Homo sapiens*; reference gene set: c2.cp.all.v2022.1.Hs.symbols.gmt; reference gene set source R package: msigdbr (v7.5.1); ID-converted R package: org.Hs.eg.db. The results of the enrichment analyses were filtered according to the following criteria: normalised enrichment score |NES| > 1, FDR < 0.25, *p.adj* < 0.05.

### 2.11 Methods for evaluation of immune cell infiltration

The infiltration frequency of immune cells in placental tissues was analyzed and compared between the normal group (placental tissues from healthy individuals) and the disease group (placental tissues from patients with specific conditions) using single-sample Gene Set Enrichment Analysis (ssGSEA) (45) implemented via the R package “GSVA”. We selected ssGSEA for its ability to provide a robust assessment of immune cell infiltration based on gene expression profiles, enabling evaluation of individual sample enrichment scores. Enrichment scores for each immune cell class were calculated using class-specific gene sets: LM22 (46), allowing assessment of immune cell infiltration in each sample. Comparisons were made between the clinically defined immune cell infiltration patterns of the two groups. Additionally, Spearman’s statistical method was used to analyze: pairwise correlations between different immune cell subtypes, and pairwise correlations between DE-AGs and immune cell proportions. The analysis results were visualized as group comparison plots, lollipop plots, and Plotted correlation scatter plots, along with data analysis and visualization of network diagrams using the R package “linkET” (v0.0.7.4), thereby enabling a more intuitive demonstration of the immune infiltration patterns associated with DE-AGs.

### 2.12 Single-cell data pre-processing and clustering annotation

High-throughput sequencing data from the single-cell dataset GSE173193 (47, 48) were obtained from the GEO database. We screened eligible samples, including two placental tissue samples from patients with gestational diabetes, two placental tissue samples from patients with pre-eclampsia, and two placental tissue samples from normal controls. The R package ‘Seurat’ (v5.1.0) (49) was used for data analysis. First, the relative proportions of mitochondrial, ribosomal, and erythrocyte genes were calculated using the Seurat function PercentageFeatureSet. Data quality was ensured by applying the following criteria: cells must express over 500 genes, genes should be present in more than three cells (prevents low-abundance artifact retention), mitochondrial gene expression must be below 25% (excludes apoptotic cells per 10x Genomics standards), ribosomal gene expression must be above 3% (ensures active translation while filtering empty droplets), and haemoglobin gene expression must be less than 1% (removes erythrocyte contamination in non-hematopoietic tissues). The dataset was normalised using the NormalizeData function, and 2000 highly variable genes were identified using the FindVariableFeatures function (50). The data were then scale-normalised using the ScaleData function. Highly variable genes were used as input features for PCA, and the RunPCA function was used to perform PCA analysis on the normalised data. To eliminate the batch effect, based on inspection of the PCA elbow plot (**Supplementary Figure 3**.) which revealed that the first 15 principal components captured the majority of variance while minimizing noise from additional dimensions, the Harmony algorithm (51) from the R package ‘harmony’ (v1.2.0) was used to select these dimensions for single-cell RNA sequencing data integration. Batch-corrected single-cell RNA sequencing data were visualised using the t-distributed stochastic neighbour embedding (t-SNE) method (52). Initially, cell-cell relationships were established using neighbourhood maps. Subsequently, clustering analysis was performed using the FindClusters function at a resolution of 0.3 to distinguish various cell populations, with these clustering results serving as the basis for further analyses. We manually annotated the data by integrating established lineage markers and consulting the human placental cell atlas available on the CellMarker website (http://xteam.xbio.top/CellMarker/)(version: CellMarker 1.0) to ensure annotation accuracy and reliability (53)(“Marker genes can be found in **Supplementary File 1**”). DEGs in each cell cluster were identified using the Wilcoxon rank sum test via Seurat’s FindMarkers function, with criteria of p_val < 0.05 and abs (avg_log2 FC) > 0.5.

### 2.13 Cell-cell communication

We employed the R package ‘CellChat’ (v1.6.1) (54) to analyse potential cell-cell interactions. In the present study, we focused on the extravillous trophoblast (EVT) cell population. First, PE samples were extracted, and 3000 cells were randomly selected to create CellChat objects. We then used the ‘human’ related data from the CellChatDB database (http://www.cellchat.org/). The ‘secretion signalling’ subset was prioritized for analyzing ligand-receptor interactions due to its critical involvement in cell-cell communication mechanisms essential for EVT cell function, particularly in placental development and crosstalk with the maternal immune system. This subset specifically encapsulates core ligand-receptor pairs that drive these biological processes. Overexpressed ligand-receptor pairs in CellChat objects were identified using the identifyOverExpressedGenes and identifyOverExpressedInteractions functions and mapped to PPI networks using the R package ‘CellChat’. The probability of intercellular communication was calculated using the computeCommunProb function, excluding communication between cell populations involving fewer than three cells. Finally, the probability of communication for specific pathways was refined using the computeCommunProbPathway function. The number and strength of intercellular interactions were visualised using the netVisual_circle function, and chord plots were used to show the expression of vascular endothelial growth factor (VEGF), insulin-like growth factor (IGF), epidermal growth factor (EGF), and macrophage migration inhibitory factor (MIF) in PE. The interactions involving VEGF, IGF, EGF, and MIF reveal critical insights into EVT cell regulatory mechanisms. For instance, VEGF is essential for angiogenesis, which is indispensable for placental development. IGF and EGF mediate cellular proliferation and differentiation, while MIF modulates immune responses. The ligand-receptor pairs involved in intercellular communication when EVT cells acted as signal senders and receivers were visualised using netVisual_bubble plots. All visualized communication probability results were subjected to significance screening using a threshold of (p < 0.05). Network centrality analysis was performed using the netAnalysis_computeCentrality function (55) and visualised as a heatmap. GDM samples were extracted, and the above process was repeated.

### 2.14 Pseudo-temporal analysis

We then performed a pseudotemporal analysis of the EVT cell population. The R package ‘monocle’ (v2.32.0) (56) was utilised for unsupervised pseudo-temporal analysis. The EVT cell clusters from the GDM and PE samples underwent further clustering analysis to identify significantly different cell clusters between the diseased and healthy samples. Then, using the gene-cell matrix at the original unique molecular identifier count scale derived from the Seurat-processed data as input, a cellular dataset containing the expression matrix, phenotypic data, and feature data was constructed using the newCellDataSet function with the parameter expressionFamily = negbinomial.size. Next, the discrete nature of the scale factors and gene expression between cells was corrected using the estimateSizeFactors and estimateDispersions functions. Dimensionality reduction was conducted using the DDRTree method (max_components set to 2), followed by cell sorting and visualisation using the plot_cell_trajectory function. DDRTree effectively captures the intrinsic structure of data, demonstrating particular suitability for single-cell RNA sequencing datasets. Specifically designed to handle complex trajectories and model branching structures, this method proves critical for pseudotime analysis of EVT cell populations. Compared to alternative dimensionality reduction techniques like PCA or t-SNE, DDRTree’s superiority lies in its capacity to preserve biologically meaningful relationships while maintaining cellular lineage associations. This capability enables accurate reconstruction of developmental trajectories, which is fundamentally important for delineating EVT cell dynamics in both physiological and pathological contexts (57). Scatter plots, violin plots, and proposed time trajectory plots were then used to display the potential marker genes screened in the bulk RNA analysis and visualised using functions inside the R package “monocle”. Pseudo-temporal highly variant genes were filtered by ‘qval < 1e-50’, ‘mean_expression ≥ 1 & dispersion_empirical ≥ 3 * dispersion_fit’, and the differentialGeneTest function was used to analyse the expression changes of these genes in pseudo-time (The threshold of qval < 1e-50 ensures that only genes with highly significant differential expression in pseudotime are included. This threshold minimizes the risk of false positives and ensures that the identified genes are robustly associated with the temporal dynamics of EVT cells. The choice of this threshold is consistent with standard practices in single-cell RNA-seq analysis). Finally, the plot_pseudotime_heatmap function was used to cluster and visualise the screened genes according to their expression patterns. We conducted KEGG enrichment analyses for each gene cluster individually using the R packages ‘clusterProfiler’ and ‘org.Hs.eg.db’. The KEGG enrichment analysis revealed several biological pathways significantly associated with the gene clusters identified in our study. These pathways provide a deeper understanding of the underlying molecular mechanisms and highlight potential therapeutic intervention targets.

### 2.15 Patient and tissue samples

Twelve placental samples were collected from women who delivered at the Third Affiliated Hospital of Wenzhou Medical University. Six of the women had PE with GDM, whereas the remaining six were healthy controls at the same gestational week of delivery. To avoid the potential effects of uterine contractions on placental metabolism during labour, all women underwent elective caesarean section for clinical reasons that did not affect placental metabolism or perfusion. All women were aged 20–40 years, had singleton pregnancies, and underwent regular obstetric examinations with complete clinical data. The Research Ethics Committee of Ruian People’s Hospital approved this study (approval number YJ2024114), and all participating mothers provided written informed consent. The inclusion criteria for the PE with GDM group were: (1) blood pressure of at least 140/90 mmHg with 24-hour urine protein levels of 0.3 g or more after 20 weeks’ gestation; (2) a 75 g oral glucose tolerance test conducted between 24 and 28 weeks’ gestation showing fasting glucose ≥5.1 mmol/L or 1-hour postprandial glucose ≥10.0 mmol/L or 2-hour postprandial glucose ≥8.5 mmol/L. The inclusion criteria for the control group were as follows: no abnormalities in blood pressure, glucose monitoring, oral glucose tolerance test, or routine urine tests. The exclusion criteria were as follows: (1) Mothers who had severe heart, liver, or kidney disease during pregnancy; preexisting hypertension, diabetes, or other serious medical or surgical conditions; or severe obstetric complications or foetal congenital diseases, including abnormal amniotic fluid volume, placenta previa, placental abruption, intrauterine distress, or foetal congenital heart disease during pregnancy or at the time of delivery; (2) those who did not undergo regular and periodic obstetric examinations; and (3) pregnant women with a history of drug, alcohol, or drug addiction or who use drugs that affect the experimental results during pregnancy and delivery. Under strictly sterile conditions, within 15 minutes after delivery, a professional doctor takes placental tissue of 1cm³ from the central part, avoiding the umbilical cord insertion point and the infarcted area. The extracted placental tissues were washed with blood in 0.9% normal saline and transferred to a refrigerator at -80°C for long-term storage.

### 2.16 Reverse transcription quantitative polymerase chain reaction

RNA was extracted using the Tissue Total RNA Isolation Kit V2 (Vazyme), followed by cDNA synthesis using HiScript III All-in-one RT SuperMix (Vazyme). RT-qPCR was performed on a CFX Connect Real-Time PCR System (Bio-Rad, Hercules, CA, USA) using Taq Pro Universal SYBR qPCR Master Mix (Vazyme). The 2−ΔΔCt method was employed to quantify relative gene expression, using GAPDH as the reference gene.

### 2.17 Statistical analysis

R software (v4.4.1) was used for data processing and analysis. Unless otherwise stated, we used the independent Student’s t-test to evaluate the statistical significance of normally distributed variables when comparing two continuous groups. We used the Mann–Whitney U-Test (Wilcoxon rank-sum test) to assess differences in non-normally distributed variables. The Kruskal–Wallis test was used to compare three or more groups. Spearman’s correlation analysis was used to calculate the correlation coefficients between different molecules. P-values were reported as two-tailed, with statistical significance set at *p* < 0.05.

## 3 Results

### 3.1 Differential expression analysis of PE with GDM

**Figure 1:**
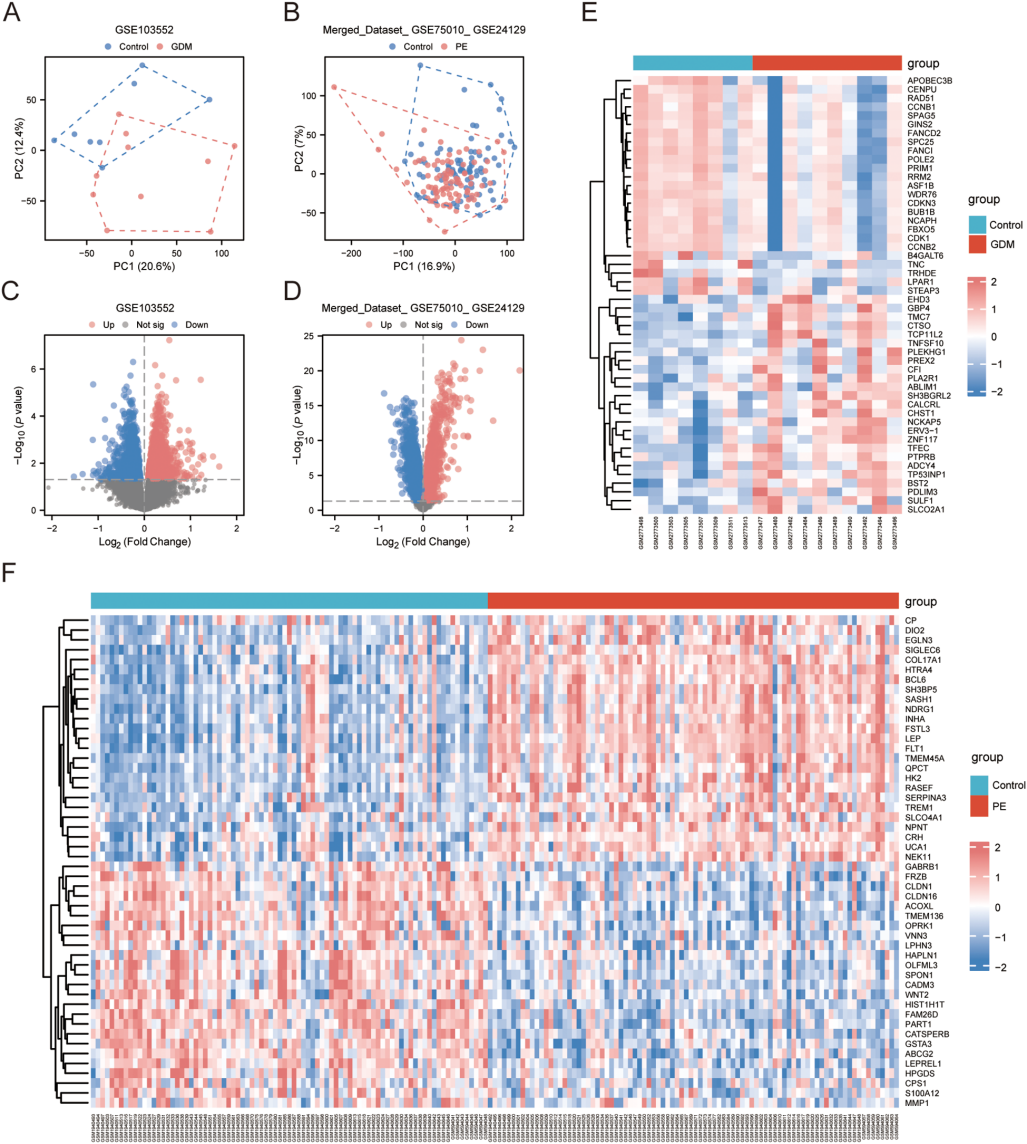
DEGs. **(A, B)** PCA of GSE103552 and Merged_Dataset_GSE75010_GSE24129 dataset. **(C, D)** Volcano plots of DEGs in GSE103552 and Merged_Dataset_GSE75010_GSE24129 datasets, |log2 FC| > 0, *p* < 0.05. **(E, F)** Expression heatmap of top 25 up- and downregulated genes in GSE103552 and Merged_Dataset_GSE75010_GSE24129 datasets. DEG, differentially expressed gene; PCA, principal component analysis.

The GSE103552 and Merged_Dataset_GSE75010_GSE24129 datasets were normalised separately. PCA was conducted, and both datasets showed more significant clustering results. In the GSE103552 dataset, PC1 was 20.6% and PC2 was 12.4% (**Figure 1A**), whereas in the Merged_Dataset_GSE75010_GSE24129 dataset, PC1 was 16.9% and PC2 was 7% (**Figure 1B**), indicating a significant difference between the groups. Volcano plot analysis of the GSE103552 dataset, using a screening threshold of |log2 FC| > 0 and *p* < 0.05, identified 2767 DEGs, with 1261 upregulated and 1506 downregulated (**Figure 1C**). In the Merged_Dataset_GSE75010_GSE24129 dataset, application of the same screening threshold revealed 6523 DEGs, with 3437 upregulated and 3086 downregulated (**Figure 1D**). The heat maps show the top 25 upregulated and downregulated genes in both datasets (**Figures 1E– F**).

### 3.2 WGCNA

**Figure 2:**
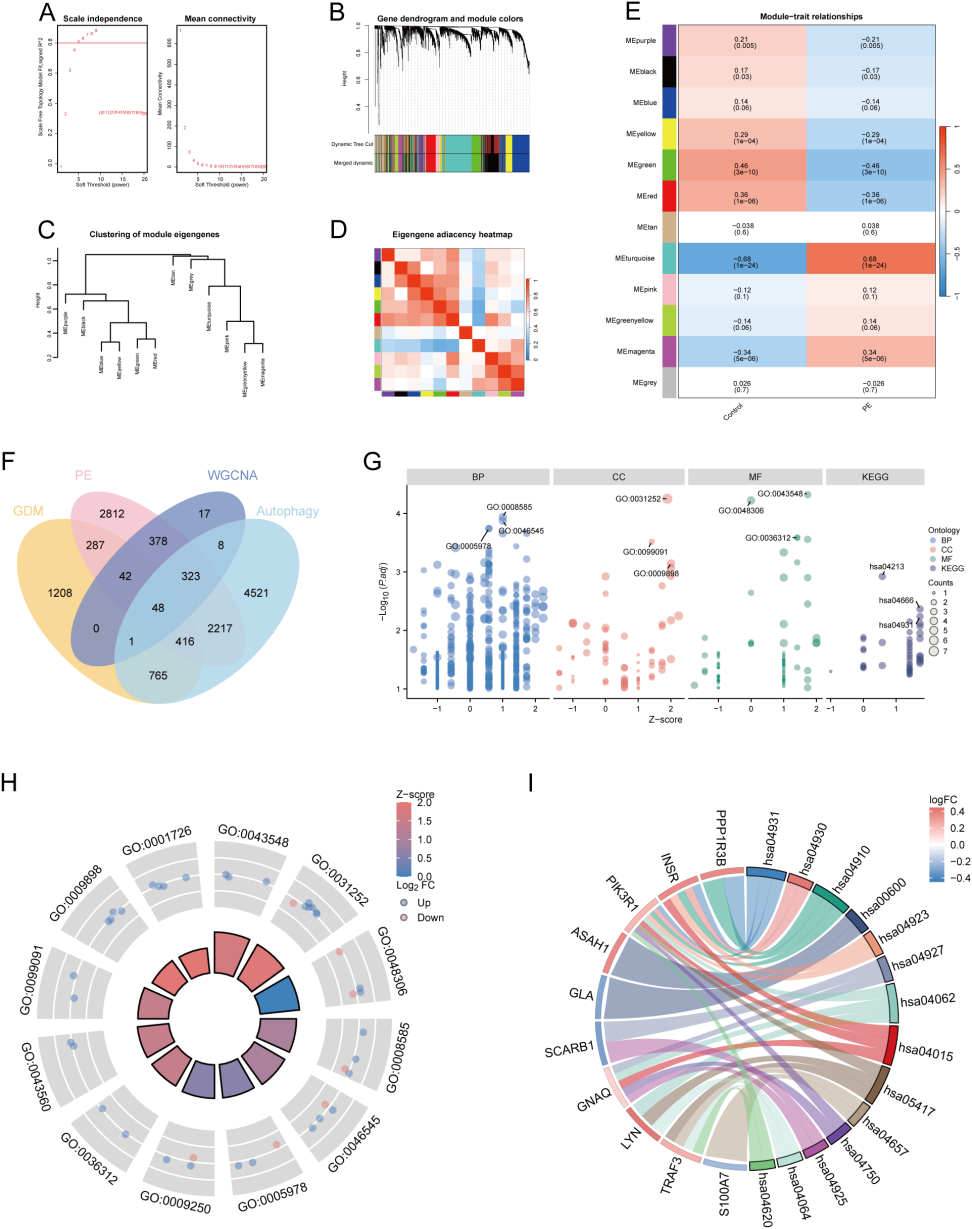
WGCNA and functional enrichment analysis of 48 DE-AGs. **(A)** Analysis of scale-free fit index and average connectivity across various soft thresholds. **(B)** Gene clustering tree integrated into a hierarchical clustering heatmap. **(C)** Module feature gene clustering tree. **(D)** Module correlation heatmap. **(E)** Gene-feature correlation heatmap. WGCNA, weighted gene co-expression network analysis. **(F)** Venn diagram plots illustrating the overlap of co-expressed genes among DEGs, MEturquoise module genes in WGCNA, and ARGs. **(G)** Enrichment analysis was conducted using GO and KEGG. GO analysis included BP, CC, and MF. **(H)** Enrichment results of 12 GO entries. **(I)** Enrichment analysis of 14 key KEGG pathways. DE-AG, differentially expressed autophagy-related gene; DEG, differentially expressed gene; WGNCA, weighted gene co-expression network analysis; ARG, autophagy-related gene; GO, Gene Ontology; KEGG, Kyoto Encyclopedia of Genes and Genomes; BP, biological processes; CC, cellular component; MF, molecular function.

Using 88 PE samples and 85 control samples from the Merged_Dataset_GSE75010_GSE24129 dataset, the top 25% of genes with the largest fluctuations were selected for WGCNA, based on the standard deviation order. Next, the pickSoftThreshold function was constructed based on the scale-free R², and the scale-free power of different soft thresholds was evaluated for scale-free scale fit indices and average connectivity (**Figure 2A**). In this study, β = 5 and scale-free R² = 0.8 were chosen as soft threshold powers. A minimum of 50 genes per module was established, with hierarchical clustering via the cutreeDynamic function used to assign genes to the modules. These modules were depicted as a dynamic shear dendrogram, and the module labels were subsequently converted to colour labels for heat map visualisation. Feature genes from each module underwent secondary hierarchical clustering, leading to the merging of highly similar modules into a new module, followed by redrawing of the heatmap (**Figure 2B**). Hierarchical clustering trees were drawn to show the clustering results of the feature genes of the modules (**Figure 2C**), and correlation heatmaps were drawn to show the correlations between the different modules (**Figure 2D**). We then identified 12 modules and calculated and visualised the correlations and p-values between the different modules and traits (**Figure 2E**). Finally, the genes in the MEturquoise module were selected as alternative genes.

### 3.3 Screening of co-expressed DEGs and results of GO and KEGG enrichment analysis

The DEGs, genes in the MEturquoise module in WGCNA, were crossed with 8299 extracted ARGs, and the Venn diagram showed that 48 DE-AGs were obtained (**Figure 2F**). The 48 DE-AGs were analysed for GO and KEGG enrichment, with 438 BPs, 47 CCs, 56 MFs, and 39 KEGGs. These were then ranked from lowest to highest FDR and visualised (**Figure 2G**). DEGs were significantly enriched in GO terms related to female gonad development, development of primary female sexual characteristics, glycogen biosynthesis, glucan biosynthesis, phosphatidylinositol 3-kinase binding, calcium-dependent protein binding, phosphatidylinositol 3-kinase regulatory subunit binding, insulin receptor substrate binding, etc. (**Figure 2H**). KEGG enrichment analysis revealed that DE-AGs were associated with pathways such as insulin resistance, type II diabetes mellitus, insulin signalling, regulation of lipolysis in adipocytes, cortisol synthesis and secretion, lipid and atherosclerosis, IL-17 signalling pathway, aldosterone synthesis and secretion, NF-kappa B signalling pathway, Toll-like receptor (TLR) signalling pathway, etc. (**Figure 2I**). Insulin resistance in GDM impairs glucose metabolism, raising blood glucose levels and triggering metabolic disturbances that can lead to preeclampsia through endothelial dysfunction and inflammation. Factors like lipolysis regulation and cortisol may worsen insulin resistance, linking obesity and stress to GDM and preeclampsia risk. The IL-17 pathway affects vascular health, while lipid metabolism and atherosclerosis connect dyslipidemia to cardiovascular issues in GDM, increasing preeclampsia risk. These pathways illustrate the complex relationship between metabolic dysregulation, inflammation, and vascular health in pregnant women with GDM.

### 3.4 PPI network

**Figure 3:**
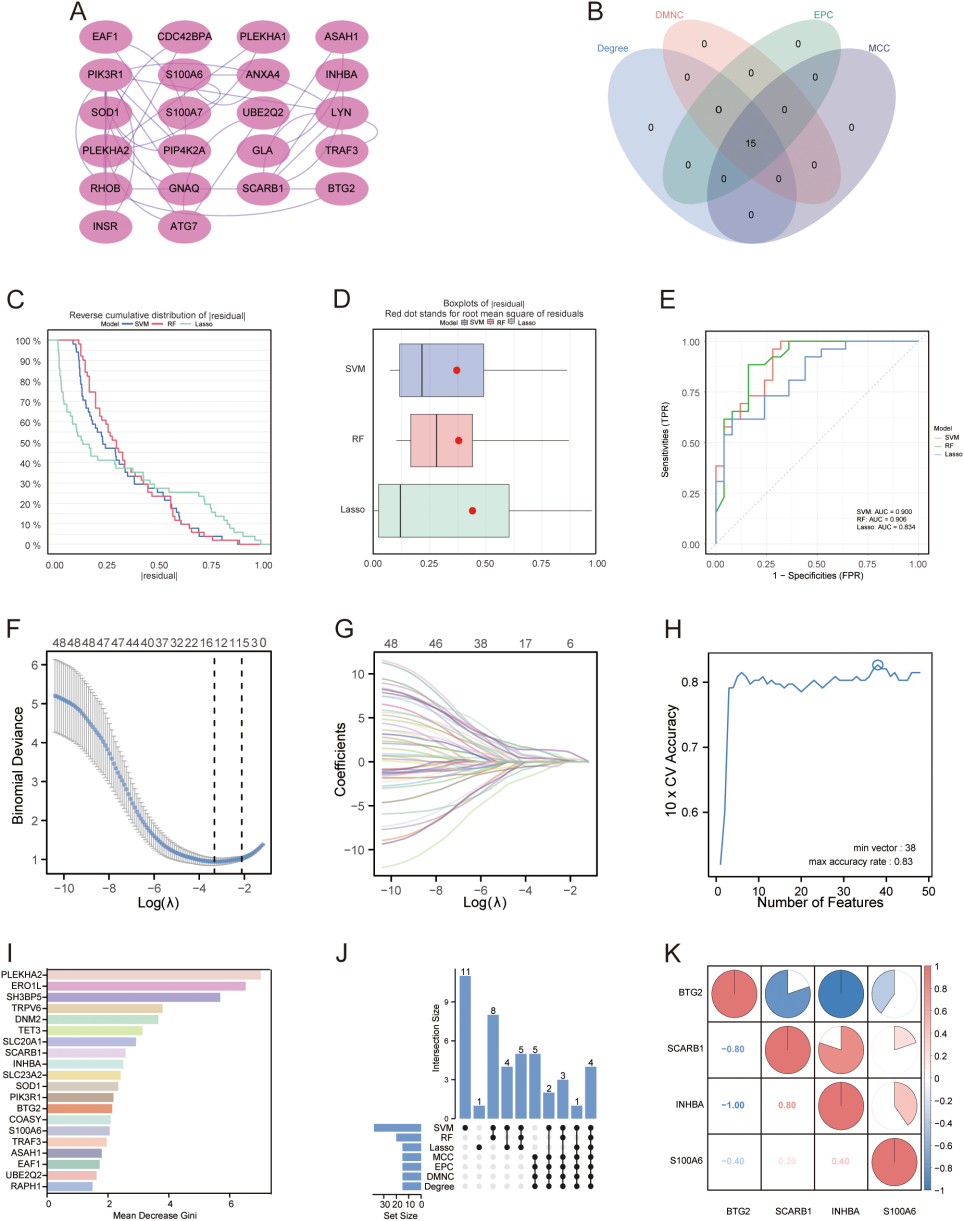
PPI networks and machine learning. **(A)** PPI network. Proteins are represented as nodes, and their interactions are depicted as edges. Shading of the node colour indicates the importance of the corresponding protein in the network. **(B)** Venn diagram illustrating the gene count overlap among MCC, Degree, EPC, and DMNC algorithms. **(C, D)** Root mean square of residuals for three machine learning models: LASSO, SVM-RFE, and RF. **(E)** ROC curves of the three machine learning models. **(F)** Cross-validation for parameter selection in LASSO regression. **(G)** LASSO regression for 48 DE-AGs. **(H)** Tenfold cross-validation with SVM-RFE used to identify the optimal feature subset. **(I)** RF algorithm for ranking feature importance based on average Gini index reduction. **(J)** Upset diagram plots illustrating the gene counts across LASSO, SVM, RF, MCC, Degree, EPC, and DMNC methods. **(K)** Correlation heatmap: used to identify correlations between four GDM with PE-related DE-AGs. PPI, protein-protein interaction; LASSO, least absolute shrinkage and selection operator; SVM-RFE, support vector machine-based recursive feature elimination, RF, random forest; GDM, gestational diabetes mellitus; PE, preeclampsia.

A PPI network of the 48 DE-AGs was constructed using the STRING database (**Figure 3A**). The top 15 hub genes were identified using the MCC, Degree, EPC, and DMNC algorithms with the CytoHubba plugin. These genes were further refined to 15 DE-AGs by overlapping the results of the four algorithms (**Figure 3B**).

### 3.5 Construction and screening of multiple machine learning models for PE with GDM-related DE-AGs

We developed machine learning models, including LASSO, SVM, and RF, utilising the expression features of 48 DE-AGs. All three models showed a low root mean square of residuals (**Figures 3C, D**). ROC analysis indicated AUC values of 0.834, 0.900, and 0.906 for the LASSO, SVM-RFE, and RF models, respectively (**Figure 3E**). We employed the LASSO, SVM-RFE, and RF methods to collectively identify hub genes for detecting GDM alongside PE-related DE-AG biomarkers. Using LASSO, 15 variables were screened: *BTG2*, *S100A6*, *PLEKHA1*, *SCARB1*, *COASY*, *DCXR*, *DNM2*, *RHOB*, *SLC23A2*, *SH3BP5*, *RELL1*, *KIAA0319*, *INHBA*, *PLEKHA2*, and *GLA* (**Figures 3F–G**). Thirty-eight significant variables were obtained by SVM-RFE, including *SH3BP5*, *ERO1L*, *TET3*, *SCARB1*, *INHBA*, *PLEKHA2*, *SOD1*, *EAF1*, *UBE2Q2*, *TRPV6*, *DNM2*, *SLC20A1*, *BTG2*, *ASAH1*, *PPP1R3B*, *PIK3R1*, *GSTA3*, *LYN*, *SLC23A2*, *S100A6*, *ANXA4*, *VTCN1*, *XPO6*, *RAPH1*, *TRAK2*, *FRMD4B*, *GLA*, *RHOB*, *KIAA0319*, *TRAF3*, *TMEM106C*, *DDIT4L*, *PLEKHA1*, *VWA5A*, *RELL1*, *CDC42BPA*, *COASY*, and *NT5E* (**Figure 3H**). The top 20 features in terms of importance were obtained using the RF model with the average Gini index reduction as an indicator, including *PLEKHA2*, *ERO1L*, *SH3BP5*, *TRPV6*, *DNM2*, *TET3*, *SLC20A1*, *SCARB1*, *INHBA*, *SLC23A2*, *SOD1*, *PIK3R1*, *BTG2*, *COASY*, *S100A6*, *TRAF3*, *ASAH1*, *EAF1*, *UBE2Q2*, and *RAPH1* (**Figure 3I**). The results obtained using the three machine learning methods and the 15 key genes obtained by the MCC, Degree, EPC, and DMNC algorithms were considered as intersections to obtain four GDM-merged PE-related DE-AGs: *BTG2*, *S100A6*, *SCARB1*, and *INHBA* (**Figure 3J**). Spearman’s correlations between the four biomarkers and their significance were calculated, and correlation heatmaps were generated (**Figure 3K**).

### 3.6 Analysis of expression differences and screening identification

**Figure 4:**
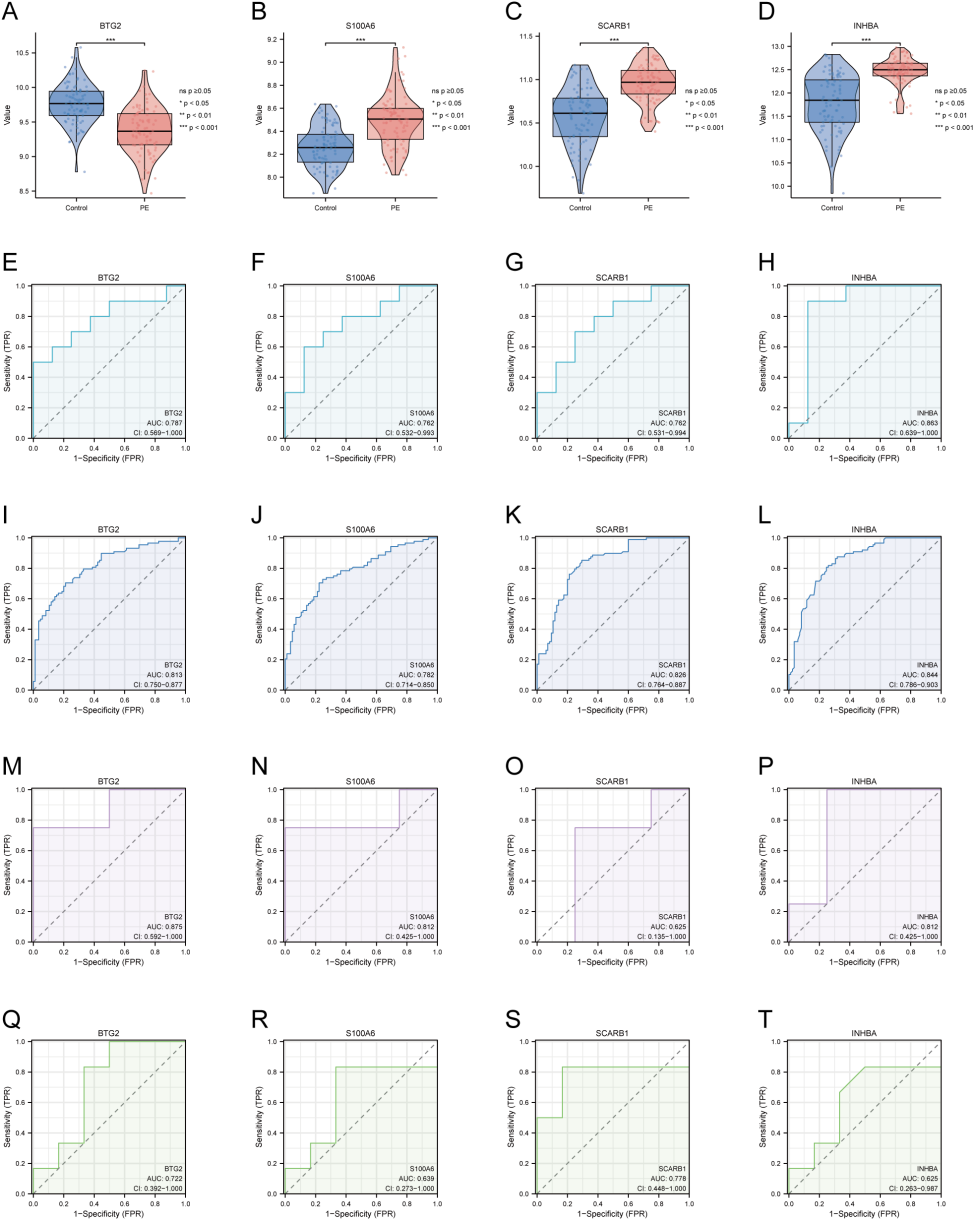
Expression of four DE-AGs and ROC validation. **(A–D)** Expression of four DE-AGs: *BTG2* **(A)**, *S100A6* **(B)**, *SCARB1* **(C)**, and *INHBA* **(D)**. **(E–H)** ROC curves for the training set GSE103552. AUC>0.700 for four DE-AGs (*BTG2*, *S100A6*, *SCARB1*, and *INHBA*). *BTG2* **(E)**, *S100A6* **(F)**, *SCARB1* **(G)**, and *INHBA* **(H)**. **(I–L)** ROC curves for the Merged_Dataset_GSE75010_GSE24129 training set. AUC>0.700 for four DE-AGs (*BTG2*, *S100A6*, *SCARB1*, and *INHBA*). *BTG2* **(I)**, *S100A6* **(J)**, *SCARB1* **(K)**, and *INHBA* **(L)**. **(M-P)** ROC curves for validation set GSE15441. AUC>0.600 for four DE-AGs (*BTG2*, *S100A6*, *SCARB1*, and *INHBA*). *BTG2* **(M)**, *S100A6* **(N)**, *SCARB1* **(O)**, and *INHBA* **(P)**. **(F–T)** ROC curves for validation set GSE30186. Note: AUC>0.600 for four DE-AGs (*BTG2*, *S100A6*, *SCARB1*, and *INHBA*). *BTG2* **(Q)**, *S100A6* **(R)**, *SCARB1* **(S)**, and *INHBA* **(T)**. DE-AG, differentially expressed autophagy-related gene; ROC, receiver operating characteristic; AUC, area under the curve; TPR, true positive rate; FPR, false positive rate. Significance levels are denoted as follows: ns *p* ≥0.05; *p < 0.05; **p < 0.01; ***p < 0.001.

**Figure 5:**
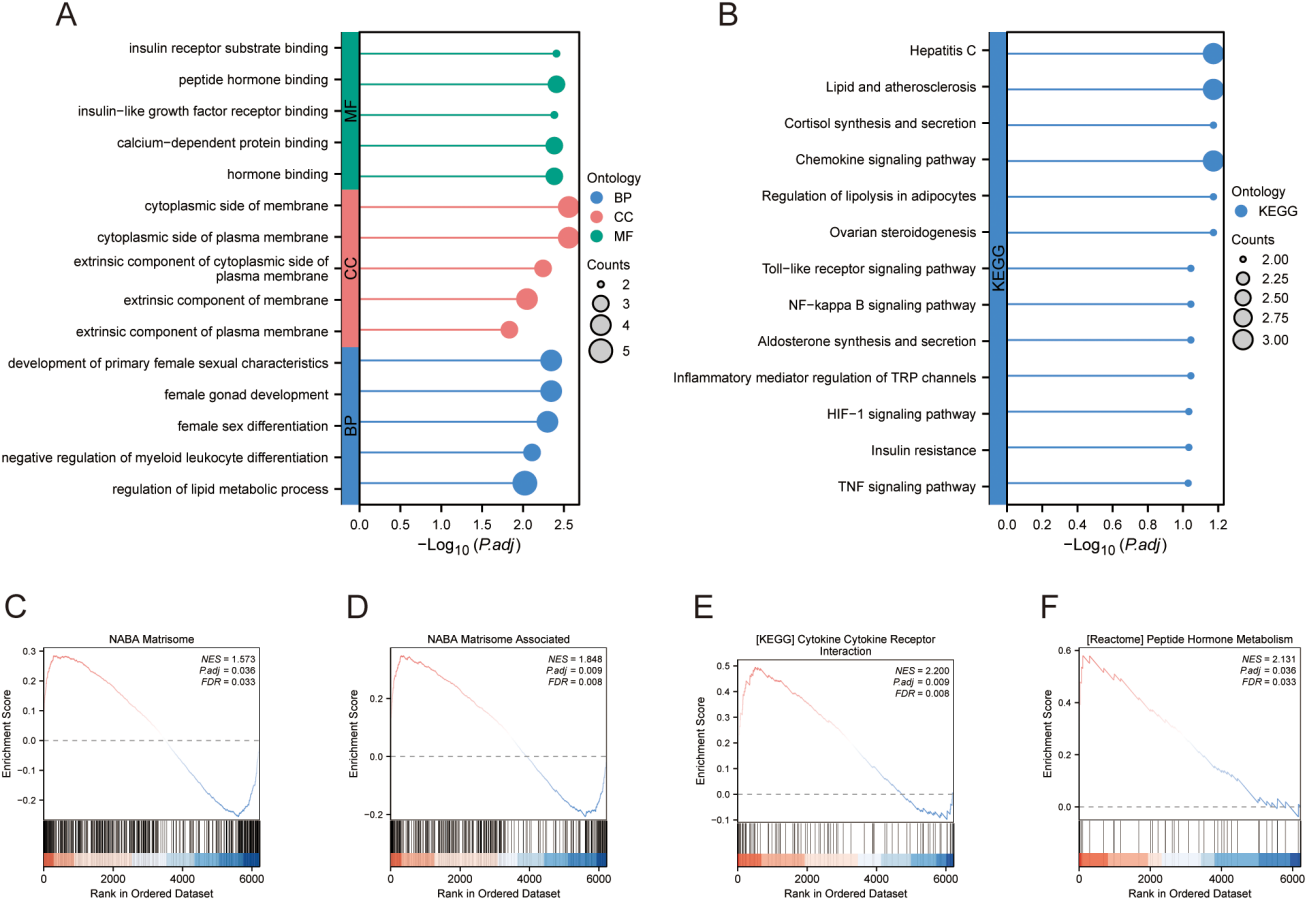
Enrichment analysis. **(A)** GO enrichment analysis. **(B)** KEGG enrichment analysis. **(C–F)** GSEA. GO, Gene Ontology; KEGG, Kyoto Encyclopedia of Genes and Genomes; GSEA, gene set enrichment analysis.

Merged_Dataset_GSE75010_GSE24129 was used as the training set to analyse the expression of the four PE with GDM-related DE-AGs. The results showed that the expression of *BTG2* was lower in the PE group than in the control group (**Figure 4A**), whereas the expression levels of *S100A6*, *SCARB1*, and *INHBA* were higher in the PE group than in the control group (**Figures 4B–D**). The diagnostic performances of the four genes were evaluated using ROC curves. Analysis of the GSE103552 dataset revealed that *BTG2*, *S100A6*, *SCARB1*, and *INHBA* each achieved an AUC exceeding 0.7, indicating a high predictive accuracy (**Figures 4E–H**). In Merged_Dataset_GSE75010_GSE24129, the AUCs of *BTG2*, *S100A6*, *SCARB1*, and *INHBA* were higher than 0.7, and their predictive ability was highly accurate (**Figures 4I–L**). External validation utilised the GSE154414 and GSE30186 datasets with diagnostic models assessed via ROC curves. The analysis of independent external datasets GSE154414 and GSE30186 validated the significant diagnostic value of *BTG2*, *S100A6*, *SCARB1*, and *INHBA*, each demonstrating AUCs exceeding 0.6, which aligned with the predicted outcomes (**Figures 4M–T**). Subsequently, GO and KEGG enrichment analyses were conducted (**Figures 5A, B**). In the KEGG enrichment analysis, *SCARB1* was mainly enriched in ovarian steroidogenesis, cortisol synthesis and secretion, and aldosterone synthesis and secretion (**Figure 5B**). In GSEA, *S100A6* and *INHBA* were mainly enriched in NABA_MATRISOME and NABA_MATRISOME_ASSOCIATED (**Figures 5C, D**). *INHBA* was predominantly associated with KEGG_CYTOKINE_CYTOKINE_RECEPTOR_INTERACTION and REACTOME_PEPTIDE_HORMONE_METABOLISM (**Figures 5E, F**).

### 3.7 Immune cell infiltration and functional analysis

**Figure 6:**
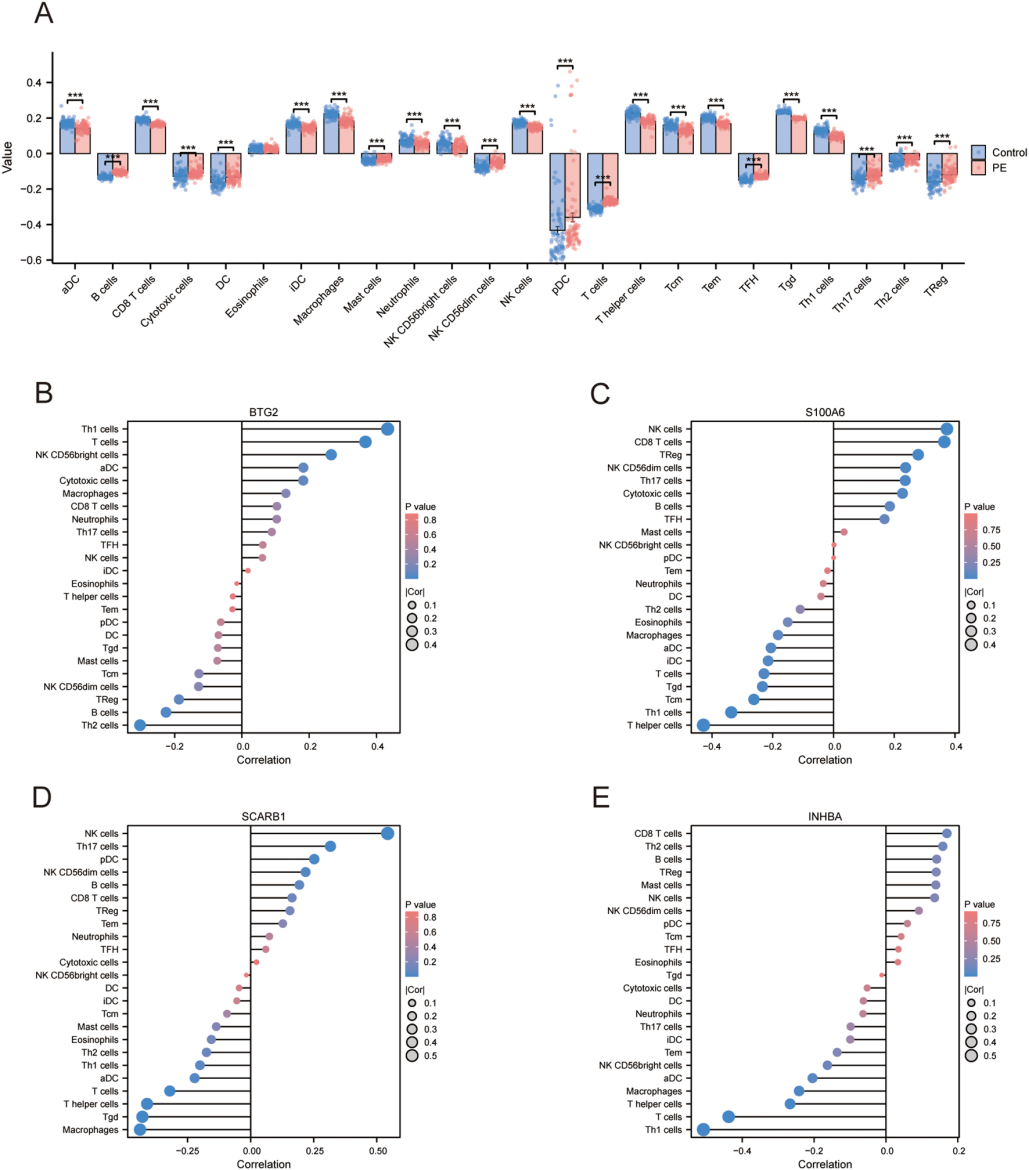
Assessment of immune cell infiltration. **(A)** Subgroup comparison plot illustrating immune cell infiltration differences between the two groups determined using the ssGSEA algorithm. Significance level is denoted as follows: *** p < 0.001. **(B)** Lollipop plot showing the correlation between *BTG2* and immune cells. **(C)** Lollipop plot illustrating the correlation between *S100A6* and immune cells. **(D)** Lollipop plot illustrating the correlation between *SCARB1* and immune cells. **(E)** Lollipop plot showing the correlation between *INHBA* and immune cells. ssGSEA, single sample gene set enrichment analysis.

A comparative analysis of immune cell infiltration revealed elevated levels of B cells, cytotoxic cells, dendritic cells, mast cells, NK CD56dim cells, plasmacytoid dendritic cells, T cells, T follicular helper cells, Th17 cells, Th2 cells, and regulatory T cells in the PE group. The levels of activated dendritic cells, CD8+ T cells, immature dendritic cells, macrophages, neutrophils, NK CD56bright cells, NK cells, T helper cells, central memory T cells, effector memory T cells, γδT cells, and Th1 cells decreased (**Figure 6A**). The correlation coefficient indicates the relationship between two variables: positive for direct correlation and negative for inverse correlation. The absolute value signifies the correlation’s strength, with 0.3-0.5 as weak, 0.5-0.8 as moderate, and 0.8–1 as strong. A *p*-value less than 0.05 denotes statistical significance. In PE cases, the correlation lollipop plots indicated that the four DE-AGs exhibited varying degrees of correlation with multiple immune cell types (**Figures 6B–E**). The expression of *BTG2* was positively correlated with the infiltration levels of Th1 and T cells (**Figure 6B**), with correlation coefficients (R) of 0.433 and 0.367, respectively (**Supplementary Figures 3A, B**); while Th2 cell infiltration levels showed a negative correlation with *BTG2* expression (**Figure 6B**), with a correlation coefficient (R) of -0.303 (**Supplementary Figure 3C**). The expression of *S100A6* was positively correlated with the infiltration levels of NK and CD8+ T cells (**Figure 6C**), with correlation coefficients (R) of 0.373 and 0.365, respectively (**Supplementary Figures 3E, F**). The expression of *S100A6* was inversely associated with T helper and Th1 cells (**Figure 6C**), with correlation coefficients (R) of -0.428 and -0.336, respectively (**Supplementary Figures 3D, G**). The expression of *SCARB1* was positively correlated with the infiltration levels of NK cells and Th17 cells (**Figure 6D**), with a correlation coefficient R of 0.543 and 0.315 (**Supplementary Figures 3H, M**). The expression of *SCARB1* was inversely associated with the infiltration levels of macrophages, γδT cells, T helper cells, and T cells (**Figure 6D**), with correlation coefficients (R) of -0.438, -0.429, -0.411, and -0.320 (**Supplementary Figures 3I–L**), respectively. A negative correlation was observed between *INHBA* expression and the infiltration levels of Th1 and T cells (**Figure 6E**), with correlation coefficients (R) of -0.508 and -0.438, respectively (**Supplementary Figures 3N, O**). There was also a correlation between different types of immune cells (**Supplementary Figure 3P**).

### 3.8 Single-cell data pre-processing and clustering annotation

**Figure 7:**
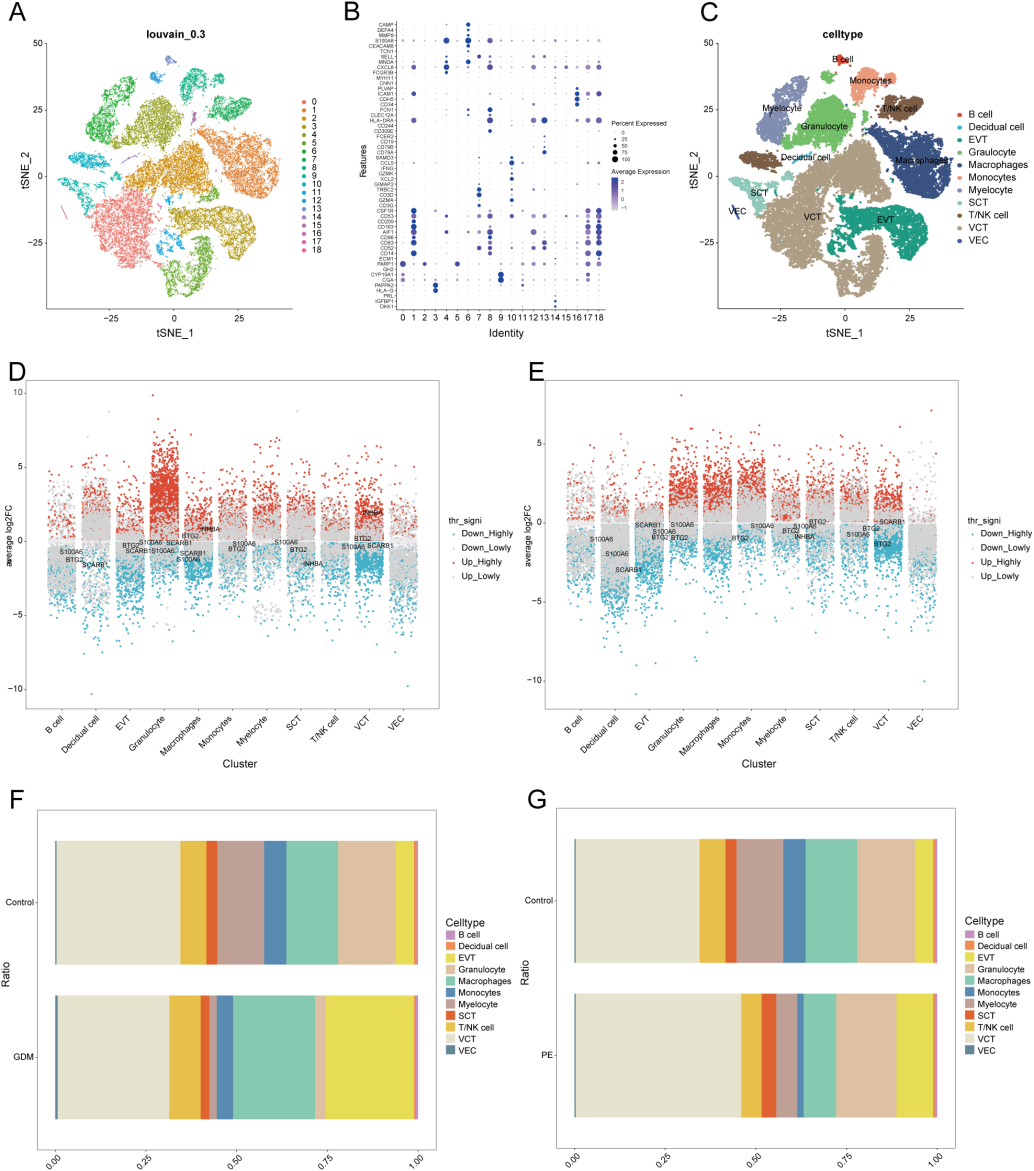
Single-cell sample clustering annotation and difference analysis. **(A)** t-SNE plot showing cell clustering results at 0.3 resolution. **(B)** Bubble plots showing marker gene expression in different clusters. **(C)** t-SNE plot after annotation. **(D, E)** Multi-subgroup volcano plots showing differential genes in GDM and PE samples, respectively. **(F, G)** Scale bar plots showing the difference in the proportion of each cell between groups in GDM and PE. t-SNE, t-distributed stochastic neighbour embedding; GDM, gestational diabetes mellitus; PE, preeclampsia.

We conducted an extensive single-cell RNA sequencing analysis on the GSE173193 dataset. At a resolution of 0.3, 19 distinct cell clusters were identified (**Figure 7A**). Bubble plots further showed the expression of signature genes in different cell clusters (**Figure 7B**). Our analysis identified 11 cell populations: B cells (marker genes were *CD79A*, *CD79B*, *CD19*, *FCER2*), decidual cells (marker genes were *DDK1*, *IGFBP1*, *PRL*), EVT (marker genes were *HLA-G*, *PAPPA2*), granulocyte cells (marker genes *FCGR3B*, *CXCL8*, *MNDA*, *SELL*), macrophages (marker genes *AIF1*, *CD14*, *CD163, CD209*, *CD53*, *CSF1R*), monocytes (marker genes *CD14*, *CD300E*, *CD244*, *HLA-DRA*, *CLEC12A*, *FCN1*), myelocytes (marker genes *TCN1*, *CEACAM8*, *S100A8*, *MMP8*, *DEFA4*, *CAMP*), syncytiotrophoblast (SCT, marker genes *CGA*, *CYP19A1*, *GH2*), T/NK cells (marker genes are *CD3G*, *GZMA*, *CD3D*, *TRBC2*, *GIMAP2*, *XCL2*, *GZMK*, *IFNG*, *CCL5*, *SAMD3*), villous cytotrophoblast (VCT, marker gene is *PARP1*), and venous endothelial cells (VECs, marker genes are *CD34*, *CDH5*, *ICAM1*, *PLVAP*). Subsequently, we applied t-SNE for visualisation (**Figure 7C**). Based on the criteria of |avg_log2 FC|>0 and p_val<0.05, we identified significant differential genes in GDM and PE samples compared with those in normal control samples using the FindMarkers function and presented these differential genes using multi-group volcano plots (**Figures 7D, E**). Notably, the potential marker genes for GDM versus PE identified in the bulk RNA analysis also showed significant differences in some cell populations in single-cell differential analysis. A significant difference in EVT distribution was observed between the control and disease groups (**Figures 7F–G**). Therefore, we selected EVT for further in-depth analysis.

### 3.9 Cell-cell communication

**Figure 8:**
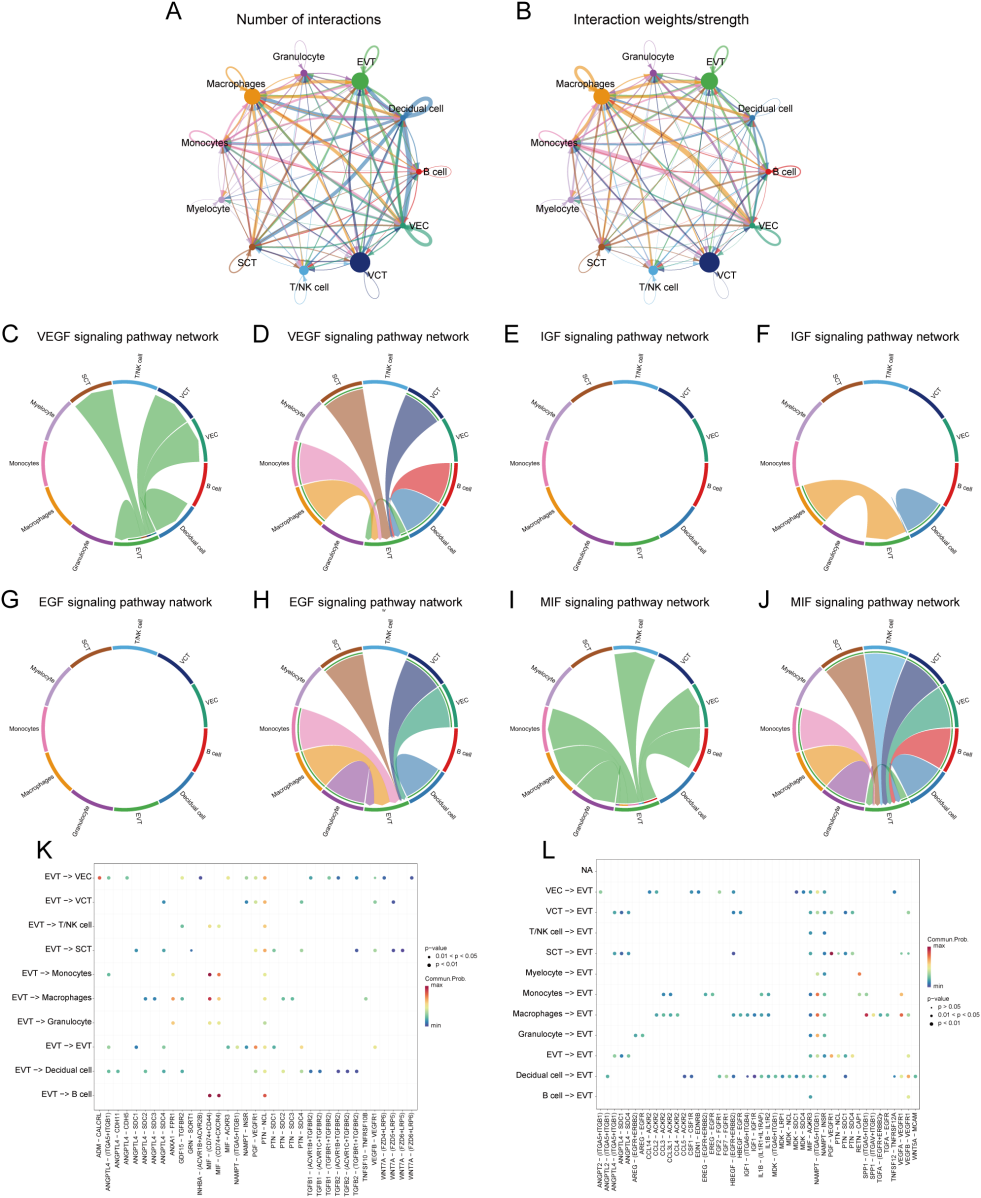
Analysis of cellular communication in GDM single-cell samples. **(A, B)** The number of interactions between cell populations and their strength in GDM samples. **(C, D)** Communication between EVT as a signal sender or receiver and other cell populations via the VEGF signalling pathway in GDM samples. **(E, F)** Communication of EVT as a signal sender and receiver via the IGF pathway with other cell populations in GDM samples. **(G, H)** Communication between GDM samples in which EVT acts as a signal sender to or receiver from other cell populations via the EGF pathway. **(I, J)** Communication between GDM samples in which EVT acts as a signal sender/receiver to or from other cell populations via the MIF pathway. **(K, L)** Ligand-receptor pairs are involved in generating communication of EVT cells as signal senders and receivers with other cells in GDM samples. GDM, gestational diabetes mellitus; EVT, extravillous trophoblast; IGF, insulin-like growth hormone; VEGF, vascular endothelial growth factor; EGF, epidermal growth factor; MIF, macrophage migration inhibitory factor.

**Figure 9:**
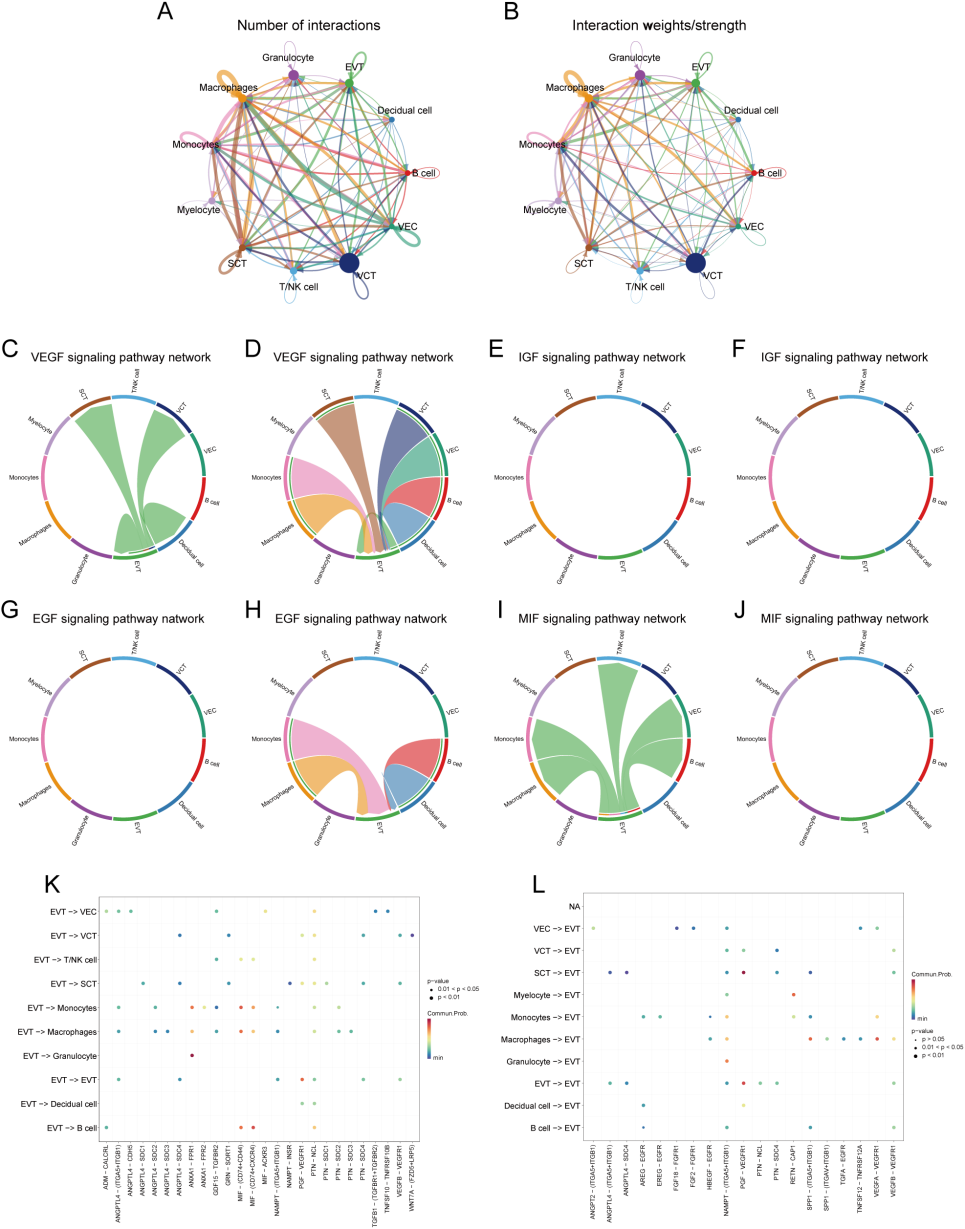
Analysis of cellular communication in PE single-cell samples. **(A, B)** Number of interactions between cell populations and their strength in PE samples. **(C, D)** Communication between EVT as a signal sender or receiver and other cell populations through the VEGF signalling pathway in PE samples. **(E, F)** Communication of EVT as a signal sender and receiver via the IGF pathway with other cell populations in PE samples. **(G, H)** Communication between PE samples in which EVT acts as a signal sender to or receiver from other cell populations via the EGF pathway. **(I, J)** Communication between PE samples in which EVT acts as a signal sender/receiver to or from other cell populations via the MIF pathway. **(K, L)** Ligand-receptor pairs are involved in the generation of communication of EVT cells as signal senders or signal receivers with other cells in PE samples. PE, preeclampsia; EVT, extravillous trophoblast; VEGF, vascular endothelial growth factor; IGF, insulin-like growth hormone; EGF, epidermal growth factor; MIF, macrophage migration inhibitory factor.

We analysed the cell-cell communication networks between different cell populations in GDM and PE samples using the R package ‘CellChat’. Circle plots show the number of interactions and their strength between cell populations in GDM (**Figures 8A, B**) and PE (**Figures 9A, B**). In GDM, chord plots showed communication with other cells through the VEGF (**Figures 8C, D**), IGF (**Figures 8E, F**), EGF (**Figures 8G, H**), and MIF (**Figures 8I, J**) pathways when EVT acted as a signal sender and receiver. We also visualised communication with other cells via the VEGF (**Figures 9C, D**), IGF (**Figures 9E, F**), EGF (**Figures 9G, H**), and MIF (**Figures 9I, J**) pathways when EVT acted as a signal transmitter and receiver in PE. The bubble diagrams show the ligand-receptor pairs involved in the communication of EVT cells as signal senders and receivers with other cells in GDM (**Figures 8K, L**) and PE (**Figures 9K, L**). As a signal transmitter, EVT communicated with SCT, VCT, decidual cells, and EVT itself via the VEGF pathway in both GDM and PE (**Figures 8C**, **9C**). EVT, as a signal receiver, communicated with macrophages, monocytes, SCT, VCT, B cells, and decidual cells via the VEGF pathway (**Figures 8D**, **9D**). As a signal sender, EVT did not communicate with other cells via the IGF pathway in either GDM or PE (**Figures 8E**, **9E**). EVT as a signal receiver generated communication with macrophages and decidual cells via IGF in GDM, but not with others via the IGF pathway in PE (**Figures 8F**, **9F**). As a signal emitter, EVT did not generate communication with other cells via the EGF pathway in either GDM or PE (**Figures 8G**, **9G**). As a signal receiver, EVT communicated with macrophages, monocytes, and decidual cells via EGF in both GDM and PE (**Figures 8H**, **9H**). As a signal transmitter, EVT communicated with macrophages in both GDM and PE, monocytes, T/NK cells, VECs, and B cells through the MIF pathway in both GDM and PE cells (**Figures 8I**, **9I**). EVT, as a signal receiver, communicated with cells other than myeloid cells through the MIF pathway in GDM but not in PE (**Figures 8J**, **9J**). We then performed a network centrality analysis of cellular communication in the GDM and PE samples, revealing the possible roles of EVT cell populations in the VEGF, IGF, EGF, and MIF pathways in cellular communication in GDM (**Supplementary Figures 5A–D**) and PE (**Supplementary Figures 5G–I**). We then comprehensively analysed the roles that different cell populations may play in the overall communication network in GDM (**Supplementary Figures 5E, F**) and PE (**Supplementary Figures 5J, K**).

### 3.10 Proposed temporal trajectory analysis

**Figure 10:**
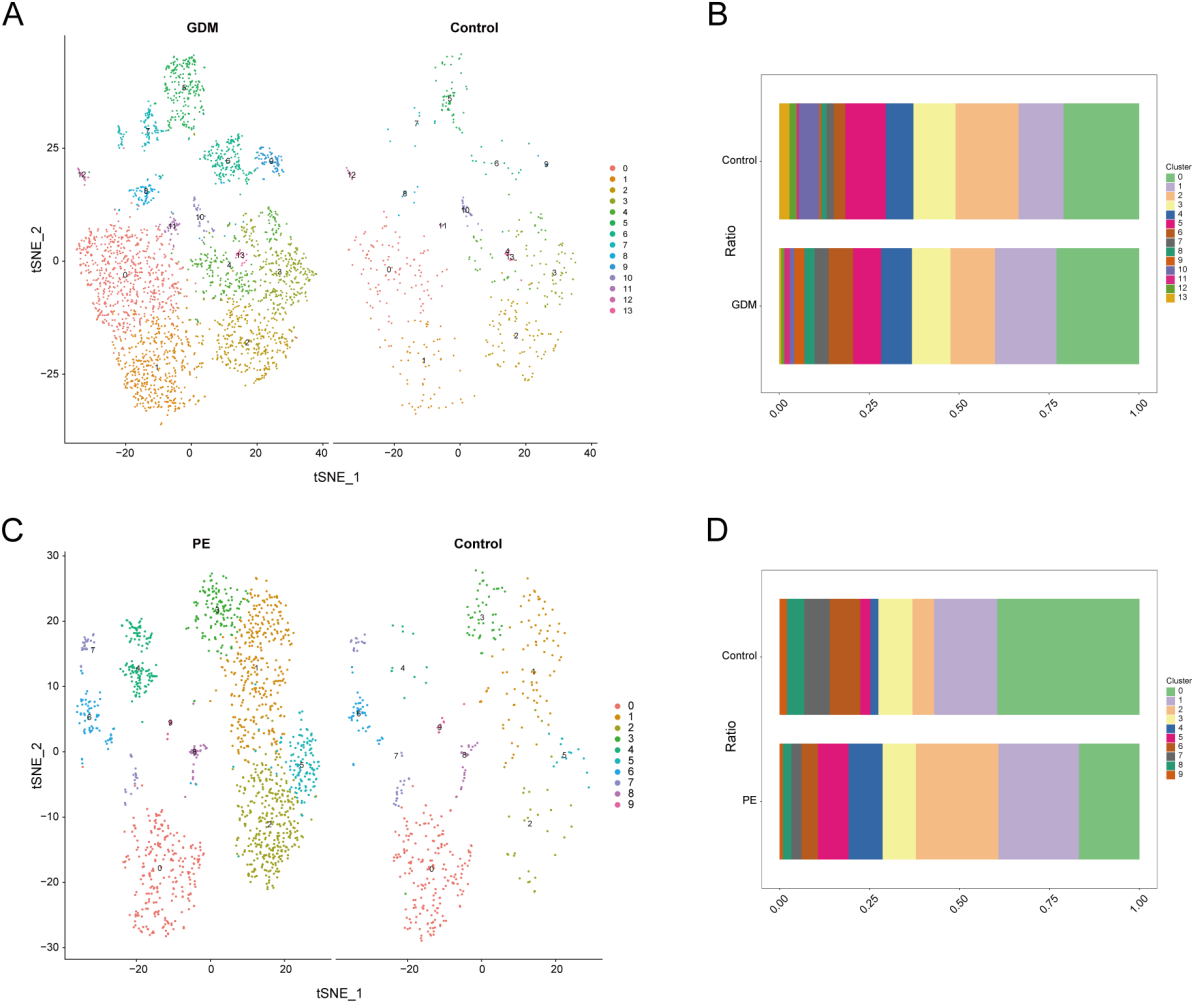
Clustering analysis of EVT cell population subpopulations. **(A)** EVT subpopulations of GDM samples. **(B)** Histogram showing the proportion of each EVT subpopulation in GDM samples. **(C)** EVT subpopulations of PE samples. **(D)** Histogram showing the proportion of each EVT subpopulation in PE samples. EVT, extravillous trophoblast; GDM, gestational diabetes mellitus; PE, preeclampsia.

**Figure 11:**
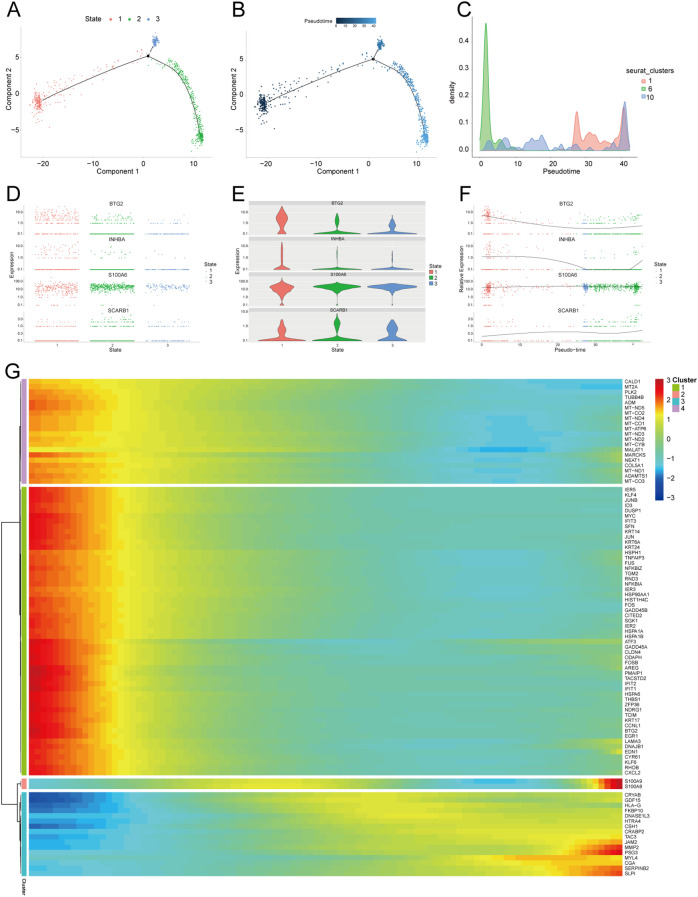
Proposed temporal analysis of GDM single-cell samples. **(A)** Three stages of EVT in GDM samples in the proposed temporal trajectory analysis. **(B)** The direction of differentiation and evolution of EVT in GDM samples in the proposed temporal trajectory analysis. **(C)** Cell density plots of EVT in GDM samples along the time axis. **(D, E, F)** Fluctuation of expression of potential biomarkers during EVT mimetic time course in GDM samples. **(G)** Heatmap illustrating expression patterns of significantly DEGs in EVT from GDM samples. GDM, gestational diabetes mellitus; EVT, extravillous trophoblast; DEG, differentially expressed gene.

**Figure 12:**
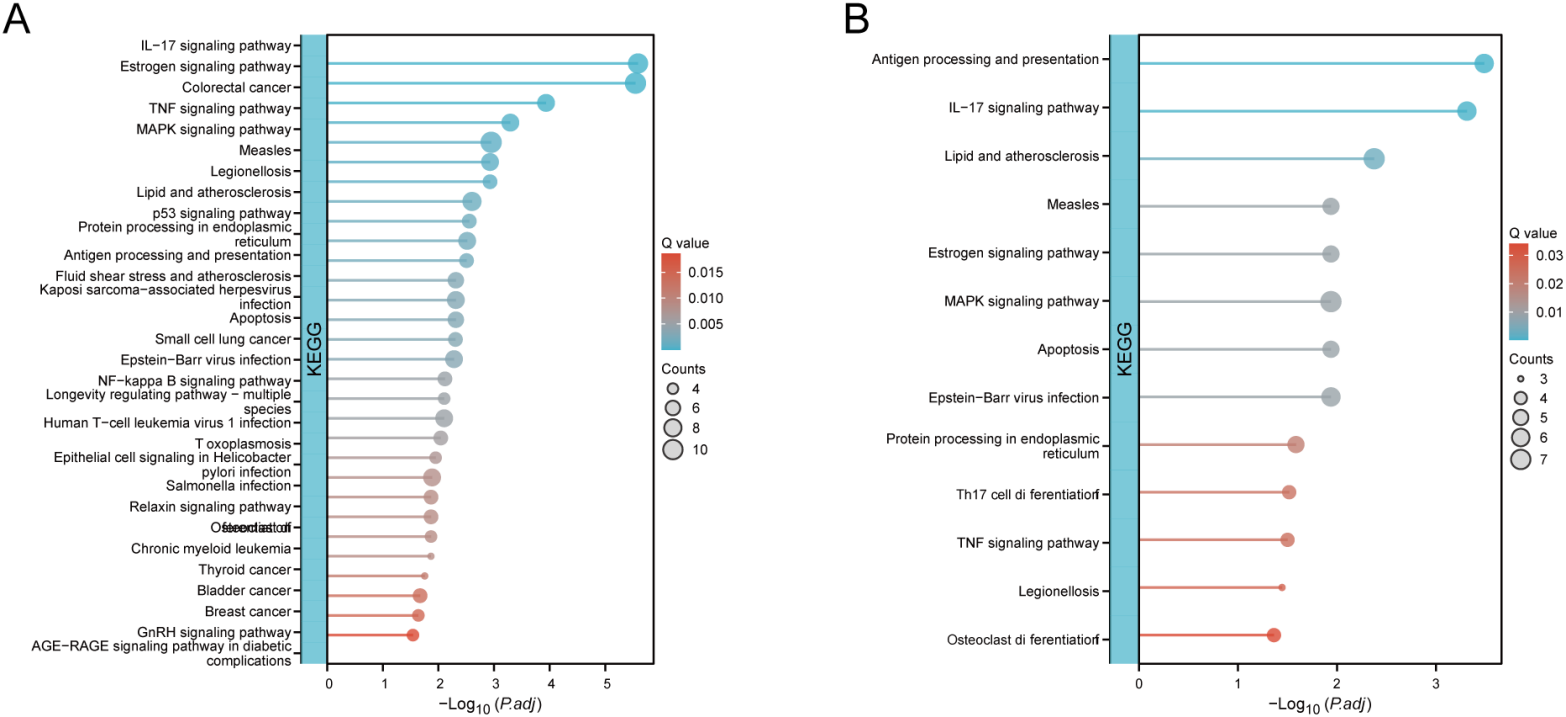
KEGG enrichment analysis of significantly DEGs. **(A)** KEGG enrichment analysis conducted on significantly DEGs in GDM samples with EVT during mimicry. **(B)** KEGG enrichment analysis conducted on significantly DEGs in PE samples with EVT during mimicry. KEGG, Kyoto Encyclopedia of Genes and Genomes; DEG, differentially expressed gene; GDM, gestational diabetes mellitus; EVT, extravillous trophoblast; PE, preeclampsia.

**Figure 13:**
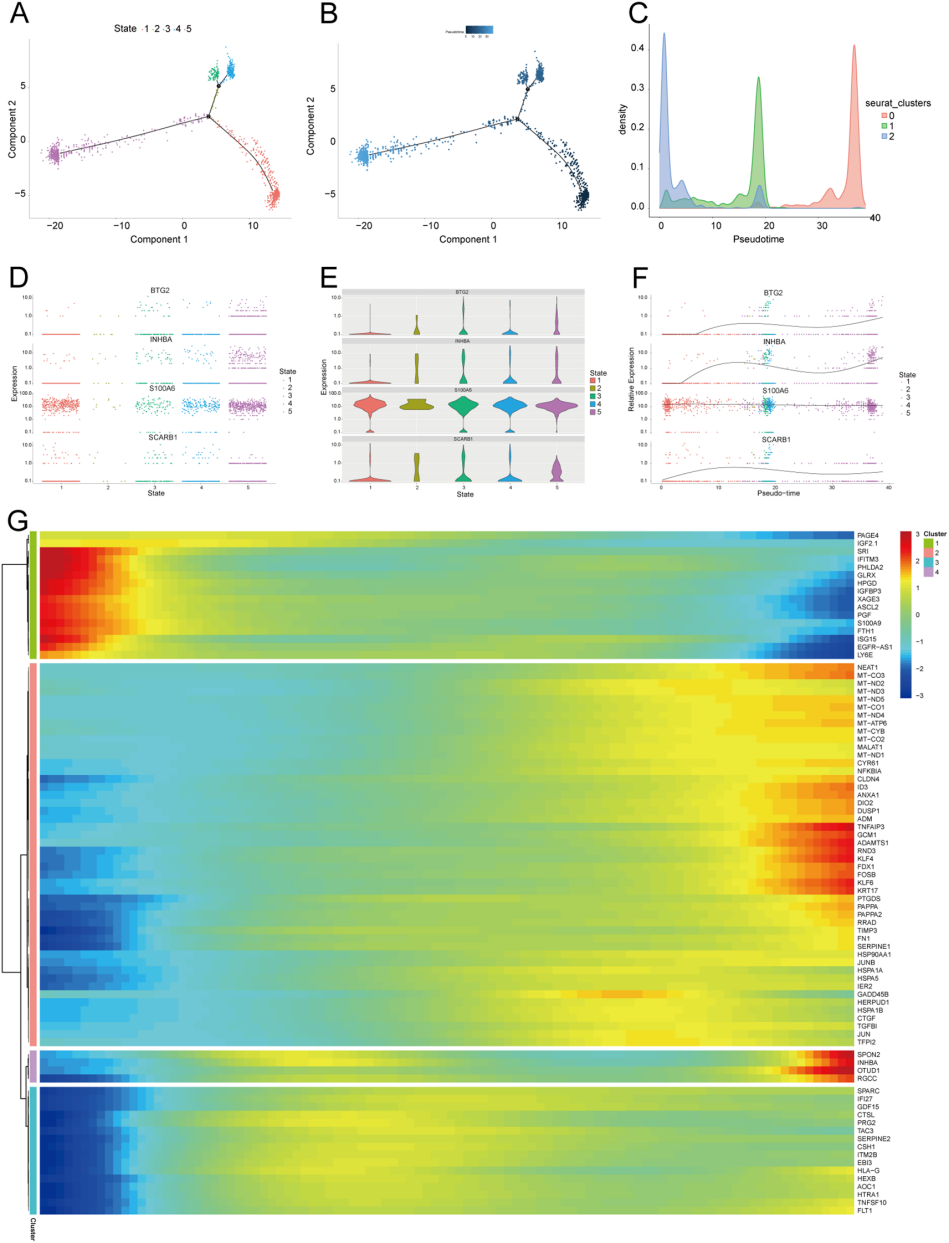
Proposed temporal analysis of PE single-cell samples. **(A)** Five stages of EVT in PE samples in the proposed temporal trajectory analysis. **(B)** Direction of differentiation and evolution of EVT in PE samples in the proposed temporal trajectory analysis. **(C)** Cell density plots of EVT in PE samples along the time axis. **(D-G)** Fluctuation of expression of potential biomarkers in PE samples of EVT during the proposed time course. Heatmap illustrating expression patterns of significantly DEGs in EVT from PE samples. PE, preeclampsia; EVT, extravillous trophoblast.

We further extracted EVT cells from the GDM and PE samples and applied the standard SeuratV5 procedure. In the GDM samples, EVT cells were reclustered into 14 subpopulations (**Figure 10A**). Scaled bar graphs were plotted to visualise the distinct subpopulations between the GDM group and healthy controls (**Figure 10B**). In the PE samples, EVT cells were reclustered into 10 subpopulations (**Figure 10C**), and scaled bar graphs were used to visualise significant differences in the subpopulations between the PE group and healthy controls (**Figure 10D**). Subpopulations 1, 6, and 10 were selected from the GDM samples, and subpopulations 0, 1, and 2 were selected from the PE samples for subsequent analyses. We analysed the proposed time-series trajectories for selected EVT subpopulations in the GDM and PE samples. In the GDM samples, the entire trajectory was divided into three phases (**Figure 11A**). **Figure 11B** shows the direction of cell differentiation. The cell density maps along the time axis further demonstrate the distribution and dynamics of EVT during the proposed time course (**Figure 11C**). We examined the expression changes of four potential biomarkers, *BTG2*, *INHBA*, *S100A6* and *SCARB1*, during the mimetic process and found that *BTG2*, *INHBA*, and *SCARB1* showed large fluctuations during the mimetic process (**Figures 11D–F**), indicating that these factors could play a crucial role in EVT cell development. We analysed the expression patterns of significantly DEGs in EVT during mimicry and categorised them into four distinct clusters (**Figure 11G**). We conducted KEGG enrichment analysis on the significantly DEGs, applying thresholds of *p.adj*<0.05 and qvalue<0.25. This analysis identified 33 enriched pathways, the top 30 of which were visualised using a lollipop plot (**Figure 12A**). For the PE samples, the entire trajectory was divided into five stages (**Figure 13A**), and **Figure 13B** shows the direction of cell differentiation. Cell density maps along the time axis further demonstrated the distribution and dynamics of EVT during the mimetic process (**Figure 13C**). We examined the expression changes of four potential biomarkers, *BTG2*, *INHBA*, *S100A6*, and *SCARB1*, during the mimetic process and found that *BTG2*, *INHBA*, and *SCARB1* showed large fluctuations during the mimetic process (**Figures 13D–F**), suggesting that they may be important factors during EVT cell development. We analysed the expression patterns of significantly DEGs in EVT during mimicry, categorising these genes into four distinct clusters (**Figure 13G**). We conducted KEGG enrichment analysis on significantly DEGs, identifying 13 pathways with *p.adj*<0.05 and qvalue<0.25, which were visualised using a lollipop graph (**Figure 12B**).

### 3.11 Validation of key genes in PE with GDM

**Figure 14:**
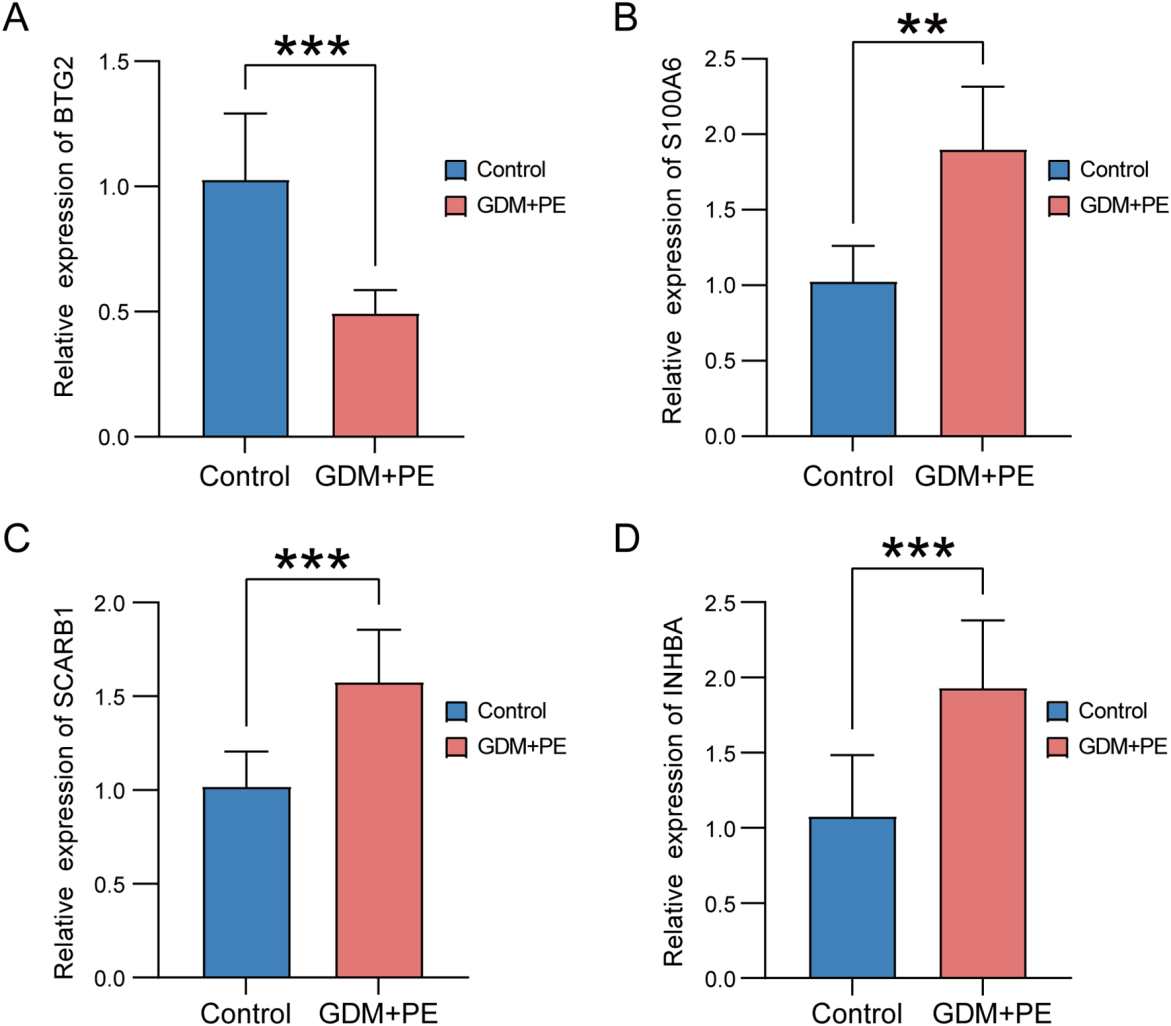
Expression of key genes in placental samples from control versus PE with GDM groups. Expression bars depict the levels of key genes *BTG2* **(A)**, *S100A6* **(B)**, *SCARB1* **(C)**, and *INHBA* **(D)** in both control and PE with GDM groups. Significance levels are denoted as follows: **p < 0.01; ***p < 0.001. PE, preeclampsia; GDM, gestational diabetes mellitus.

We used RT-qPCR to determine the expression levels of the four key genes in placental samples. The analysis included six samples of PE with GDM and six control samples. Refer to **Supplementary Table S1** for the primer sequences. RT-qPCR analysis revealed significantly reduced *BTG2* expression in placental samples from patients with PE and GDM (**Figure 14A**), and the expression levels of *S100A6*, *SCARB1*, and *INHBA* (**Figures 14B–D**) were significantly higher in placental samples from patients with PE complicated by GDM than in those from the control group. The significance levels are denoted as follows: **p < 0.01, ***p < 0.001.

## 4 Discussion

Through comprehensive bioinformatics analysis combining differential expression, WGCNA, and machine learning approaches (LASSO, SVM-RFE, RF), we identified 48 autophagy-related genes (DE-AGs) associated with PE and GDM. Subsequent PPI network analysis and hub gene screening revealed four key candidates: *BTG2* (downregulated), *S100A6*, *SCARB1*, and *INHBA* (all upregulated) in PE with GDM patients compared to controls. While these genes have established roles in other pathologies - *BTG2* in cell cycle regulation (58), *S100A6* in inflammation (59), *SCARB1* in lipid metabolism (60), and *INHBA* in reproductive biology (61) - their specific functions in PE and GDM remain poorly characterized. This knowledge gap underscores the need for further investigation into these potential biomarkers and their shared molecular mechanisms to improve clinical management of these pregnancy complications.

GO and KEGG analyses of DE-AGs revealed significant enrichment of DE-AGs in autophagy-related pathways including PI3K binding, insulin signaling, NF-kappa B signalling, and TLR signaling. The PI3K pathway serves as a central regulator of cell growth and metabolism (62), with its activation promoting autophagosome formation (63). Insulin signaling mediates glucose homeostasis (64), while PI3K-dependent Akt phosphorylation activates downstream effectors including NF-κB, a key mediator of inflammatory responses implicated in chronic diseases (65). These pathways exhibit complex cross-regulation - PI3K/Akt activation can suppress NF-κB to enhance autophagy (66), while NF-κB may reciprocally modulate PI3K/Akt activity (67). TLRs initiate immune responses through pathogen recognition and subsequently regulate autophagy via NF-κB and PI3K/Akt/mTOR pathways (68). Notably, our identified DE-AGs functionally intersect with these pathways: *BTG2* modulates both insulin signaling and NF-κB-mediated inflammation (69, 70); *S100A6* participates in TLR signaling (71) *SCARB1* activates PI3K/Akt; and *INHBA* regulates NF-κB-dependent cellular processes (72). These findings position these genes as potential key regulators in PE and GDM pathogenesis through their involvement in these critical pathways.

Enrichment analyses revealed other notable BPs and signalling pathways, including the development of primary female sexual characteristics, glycogen biosynthesis, glucan biosynthesis, calcium-dependent protein binding, and insulin receptor substrate binding. These pathways may contribute significantly to the pathophysiology of PE in GDM. For example, the enrichment of pathways such as insulin resistance and type II diabetes mellitus suggests a key role of metabolic dysregulation in the disease, whereas the enrichment of pathways such as lipid and atherosclerosis emphasises the impact of abnormal lipid metabolism on the development of the disease. Enrichment of the IL-17 signalling pathway, along with aldosterone and cortisol synthesis and secretion, underscores the significance of inflammatory response and endocrine regulation in PE with GDM.

This study utilised ssGSEA to evaluate the variations in immune cell infiltration between patients with PE and normal controls. A significant increase in the infiltration of B cells, cytotoxic cells, dendritic cells, mast cells, NK CD56dim cells, plasmacytoid dendritic cells, T cells, follicular helper T cells, Th17 cells, Th2 cells, and regulatory T cells was observed in the PE group. The infiltration levels of various immune cells, including activated dendritic cells, CD8+ T cells, immature dendritic cells, macrophages, neutrophils, NK CD56bright cells, NK cells, T helper cells, central memory T cells, effector memory T cells, γδT cells, and Th17 cells, were significantly reduced. PE is widely believed to be associated with placental abnormalities resulting in insufficient uterine placental blood flow and subsequent maternal endothelial dysfunction. Endothelial dysfunction is thought to be caused by an imbalance between pro-and antiangiogenic factors, oxidative stress, and excessive inflammatory response (73). Our study confirmed that significant alterations occurred in the immune microenvironment of patients with PE, highlighting the crucial role of the immune system and immune cell-mediated inflammation in the progression of PE (74).

Immune infiltration analyses revealed significant correlations between the four key DE-AGs (*BTG2*, *S100A6*, *SCARB1*, and *INHBA*) and the infiltration levels of several immune cells. *BTG2* expression was positively correlated with Th1 and T cell infiltration but negatively correlated with Th2 cell infiltration. *S100A6* expression positively correlated with NK and CD8+ T cell infiltration and negatively correlated with helper T and Th1 cell infiltration. *SCARB1* expression was positively correlated with macrophage, γδ T cell, helper T cell, and T cell infiltration. *INHBA* expression was negatively correlated with Th1 and T cell infiltration. These findings underscore the significant role of immune cell infiltration in the pathophysiology of PE and indicate that these key genes may affect the disease by altering the immune microenvironment.

Our study examined the expression patterns and biological roles of four DE-AGs in individual placental cells. Conventional RNA-seq transcriptomic data pose challenges in characterising the heterogeneity of different cell types within the placenta of patients with PE and GDM, and healthy individuals. Technological advancements have led to the development of high-throughput sequencing methods such as scRNA-seq, which offer transcriptomic insights at the cellular level. Based on the scRNA-seq data, we annotated and identified 11 cellular isoforms. The results showed that *BTG2*, *S100A6*, *SCARB1*, and *INHBA* exhibited specific expression patterns in different cell types within the placenta.

*BTG2* exhibited notable differential expression in EVT, which aligned with the trends observed in the bulk RNA analysis. Further refinement of the EVT cell subtypes revealed that *BTG2* was predominantly expressed in specific EVT subpopulations in the placentas of patients with PE and GDM. Functional module scoring and enrichment analysis indicated that EVT subpopulations exhibited elevated autophagic activity and secretion of proinflammatory mediators. GSEA revealed that in patients with GDM and PE, these subpopulations activated pathways related to pro-inflammation and autophagy, influencing cell survival and metabolism regulation.

Intercellular communication analyses revealed that EVT acts as both a signal transmitter and receiver in PE and GDM, communicating with various cells through the VEGF pathway. VEGF is crucial in pregnancy, significantly influencing maternal and foetal health by enhancing placental angiogenesis and improving nutrient and oxygen delivery to both the mother and foetus. Autophagy is crucial for regulating the VEGF pathway. For example, VEGF promotes autophagy by activating adenylate-activated protein kinase, which promotes endothelial cell survival and function. In a hypoxic environment, the upregulation of VEGF expression not only promotes angiogenesis, but also attenuates cellular damage through the autophagy pathway. By analysing the cellular communication of GDM samples, we found that there was intercellular communication between EVT and VECs, and the EVT acted as a signal transmitter to associate with the VEC; however, we did not find the same communication process in PE samples. Compared with EVT in GDM, EVT changed their communication pattern with VEC in PE, and in PE EVT only acted as a signal receiver to associate with VECs, which are not present in GDM, and two diametrically opposed modes of communication between EVTs and VECs were seen in both diseases. Physiological invasion and vascular remodelling of EVT and other BPs are critical for placental health in pregnant mice (13) and this process is influenced by autophagy regulation, which when impaired leads to placental dysplasia under physiological hypoxia in early pregnancy (75), which further supports the results of our analyses. Our study showed that EVT interacts with macrophages through the VEGF, EGF, and MIF pathways in both PE and GDM, indicating potential immune factor interference in their development. Additionally, macrophage infiltration was observed in PE samples, with significant differences in infiltration proportions between the groups, further implying the influence of immune cells in the progression of PE and GDM. Related studies have confirmed that meconium macrophages can promote the remodelling of uterine spiral arteries through the production of angiogenic factors (76), and the dysregulation of macrophage polarisation may lead to insufficient remodelling of the uterus and insufficient invasion of trophoblast cells, which may trigger a series of pregnancy complications, such as spontaneous abortion, preterm delivery, and PE (77). Therefore, the immune-inflammatory response and related mechanisms in PE with GDM are of great value to be investigated.

Temporal trajectory analysis indicated that three DE-AGs—*BTG2*, *INHBA*, and *SCARB1*— showed notable changes in expression during the mimetic process of EVT in PE combined with GDM. This suggests that they have crucial roles and physiological significance in EVT development. *BTG2*, an anti-proliferative factor involved in cell cycle regulation and apoptosis, may reflect the dynamic changes in EVT cell proliferation and apoptosis in GDM and PE (58, 78). *INHBA* plays a crucial role in cell proliferation, differentiation, and autophagy regulation, and its expression levels indicate its importance in the modulation of EVT cell function (79). Moreover, through the mimetic trajectory, we found the key nodes of EVT in GDM and PE during the mimetic process and performed BEAM analysis on them respectively, finding that *BTG2* was the core gene at the branch in GDM, whereas *INHBA* was the core gene at the branch in PE, which further illustrated the core roles of *BTG2* and *INHBA* in PE merged with GDM. KEGG enrichment analysis of genes with significant differential expression at the branches revealed enrichment in lipid and atherosclerosis and NF-kappa B and TNF signalling pathways in GDM cases. In PE cases, genes were enriched in the lipid atherosclerosis and TNF signalling pathways. Additionally, four DE-AGs (*BTG2*, *INHBA*, *SCARB1*, and *S100A6*) were enriched in these pathways in bulk RNA samples, suggesting a potential link to the underlying mechanism of PE combined with GDM. Dyslipidaemia during pregnancy has been linked to both gestational hypertension and chronic hypertension postpartum (80). Additionally, histone deacetylase influences cytokine expression via NF-κB and HIF-1α pathways, potentially contributing to pregnancy-related disorders like PE (81).

This study has some limitations. First, this study focused solely on mRNA levels, necessitating further research to explore the protein-level alterations of DE-AGs in PE with GDM and their functional implications. Second, the single-cell sequencing component was constrained by a relatively small sample size (n=6), which may limit the generalizability of cell-type-specific immune infiltration patterns. The relatively small clinical sample size may also limit the universality of the results. These methodological boundaries highlight the need for expanded multi-omics validation cohorts in subsequent research. The validation of DE-AGs at the protein level is essential to confirm their functional role in PE combined with GDM. Validation at the protein level may provide insights into the post-transcriptional regulation of these genes and their interactions with other proteins and signalling pathways. Future studies should focus on validating the expression and function of DE-AGs at the protein level using techniques, such as western blotting, immunohistochemistry, and mass spectrometry. Longitudinal studies with larger sample sizes are required to determine the clinical relevance of these findings. Larger sample sizes will provide greater statistical power and allow the identification of other DE-AGs that may be involved in PE associated with GDM, and longitudinal studies will help elucidate temporal changes in DE-AG expression and its relationship to disease progression. These studies will also provide insight into the potential use of DE-AGs as predictive biomarkers for the development of PE with GDM.

## 5 Conclusion

In summary, our analysis identified key ARGs involved in the pathogenesis of PE with GDM. These findings offer insights into the molecular mechanisms underlying these diseases and help identify potential therapeutic targets. Future research should aim to validate these targets and investigate their clinical applicability in enhancing pregnancy outcomes in patients with PE combined with GDM.

## Data Availability

Publicly available datasets were analyzed in this study. This data can be found here: https://www.ncbi.nlm.nih.gov/geo/.

## Supplementary material

Supplementary Figure 1 Boxplots of the five datasets after cleaning. **(A)** GSE103552. **(B)** GSE30186. **(C)** GSE24129. **(D)** GSE154414. **(E)** GSE75010.

Supplementary Figure 2 PCAplots of the five datasets after cleaning. **(A)** GSE103552. **(B)** GSE30186. **(C)** GSE24129. **(D)** GSE154414. **(E)** GSE75010.

Supplementary Figure 3 Scatterplot with correlation network heatmap. Scatterplot showing the correlation between *BTG2* **(A–C)**, *S100A6* **(D–G)**, *SCARB1* **(H–M)**, and *INHBA* **(N, O)** and immune cells. **(P)** Heatmap of the correlation network of 24 immune cells.

Supplementary Figure 4 Elbow plot for PCA dimensions selection during Harmony normalization.

Supplementary Figure 5
**(A–D)** Network centrality analysis of four signalling pathways (VEGF, IGF, EGF and MIF) in GDM samples. **(E, F)** Possible roles of different cell populations in the overall communication network in GDM samples. **(G–I)** Network centrality analysis of three signalling pathways (VEGF, EGF and MIF) in PE samples. **(J, K)** Possible roles of different cell populations in the overall communication network in PE samples.GDM, gestational diabetes mellitus; PE, preeclampsia; VEGF, vascular endothelial growth factor; IGF, insulin-like growth hormone; MIF, macrophage migration inhibitory factor.
