# Supplementary figures and images for: Prediction and immune landscape study of potentially key autophagy-related biomarkers in preeclampsia with gestational diabetes mellitus
Source: Front Immunol. 2025 Jul 2;16:1571795. doi: 10.3389/fimmu.2025.1571795 (PMC12263617; doi:10.3389/fimmu.2025.1571795)

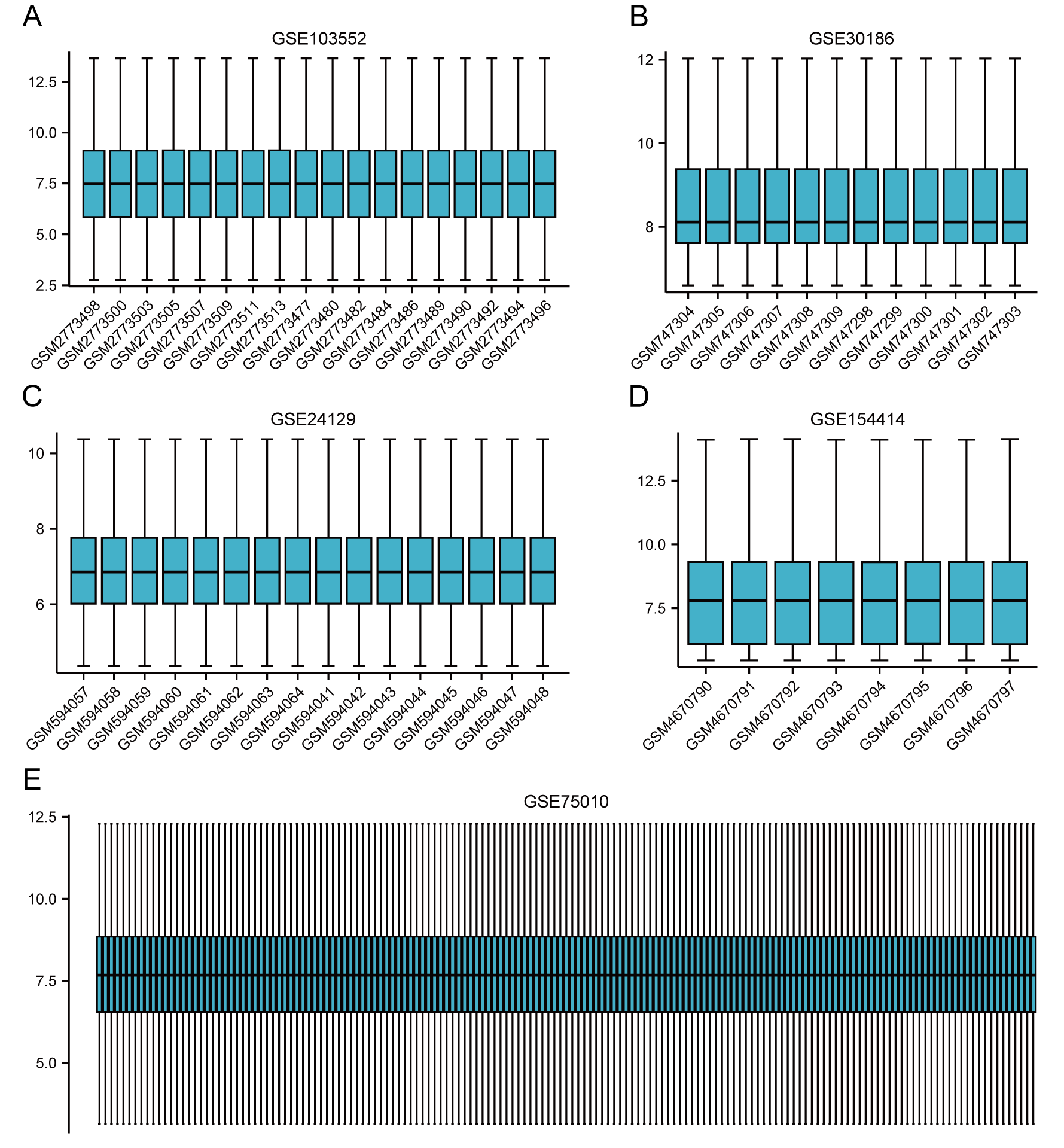

Supplement: Supplementary Figure 1 — Boxplots of the five datasets after cleaning. (A) GSE103552. (B) GSE30186. (C) GSE24129. (D) GSE154414. (E) GSE75010. [file Image1.tif]

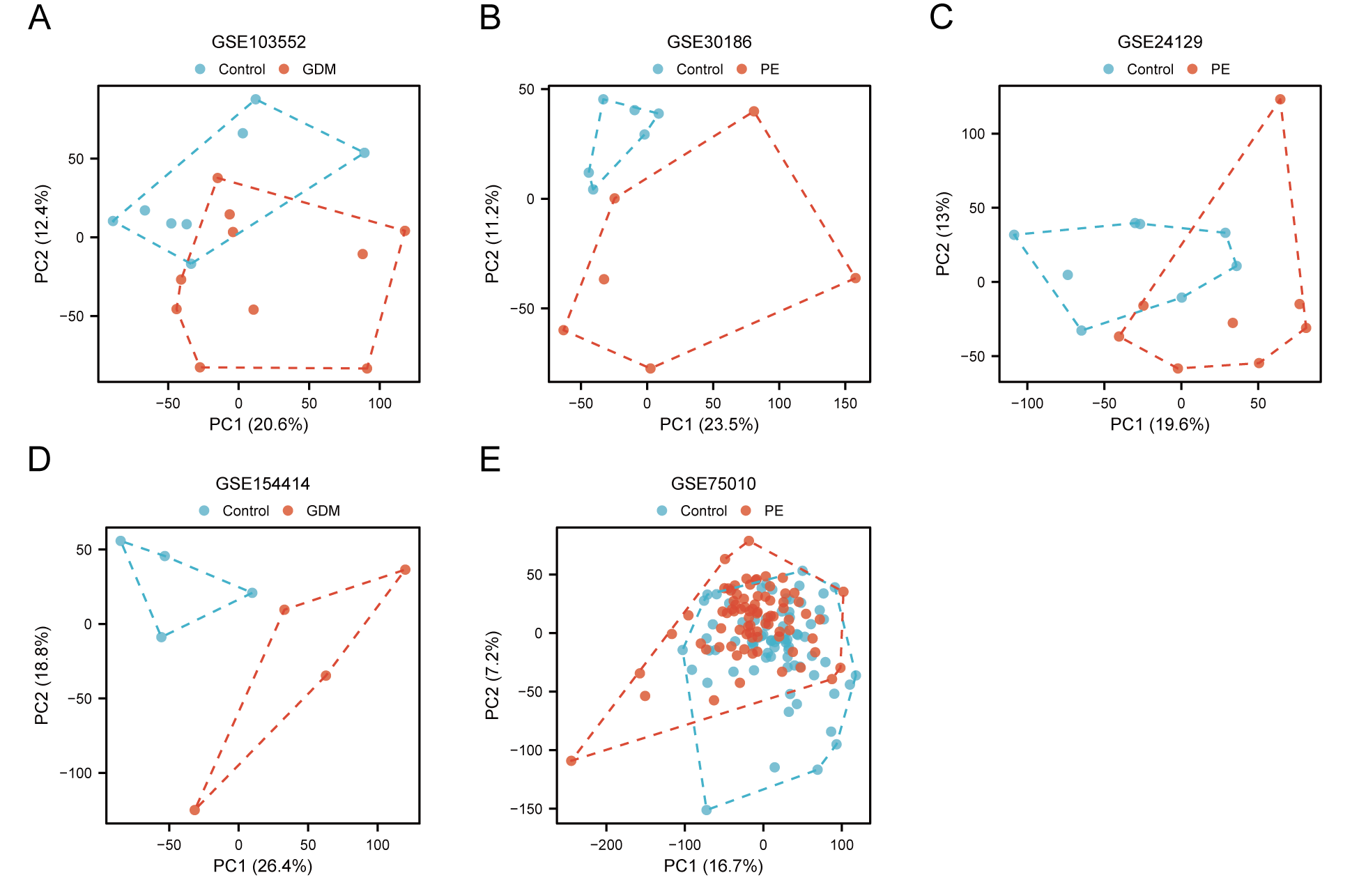

Supplement: Supplementary Figure 2 — PCAplots of the five datasets after cleaning. (A) GSE103552. (B) GSE30186. (C) GSE24129. (D) GSE154414. (E) GSE75010. [file Image2.tif]

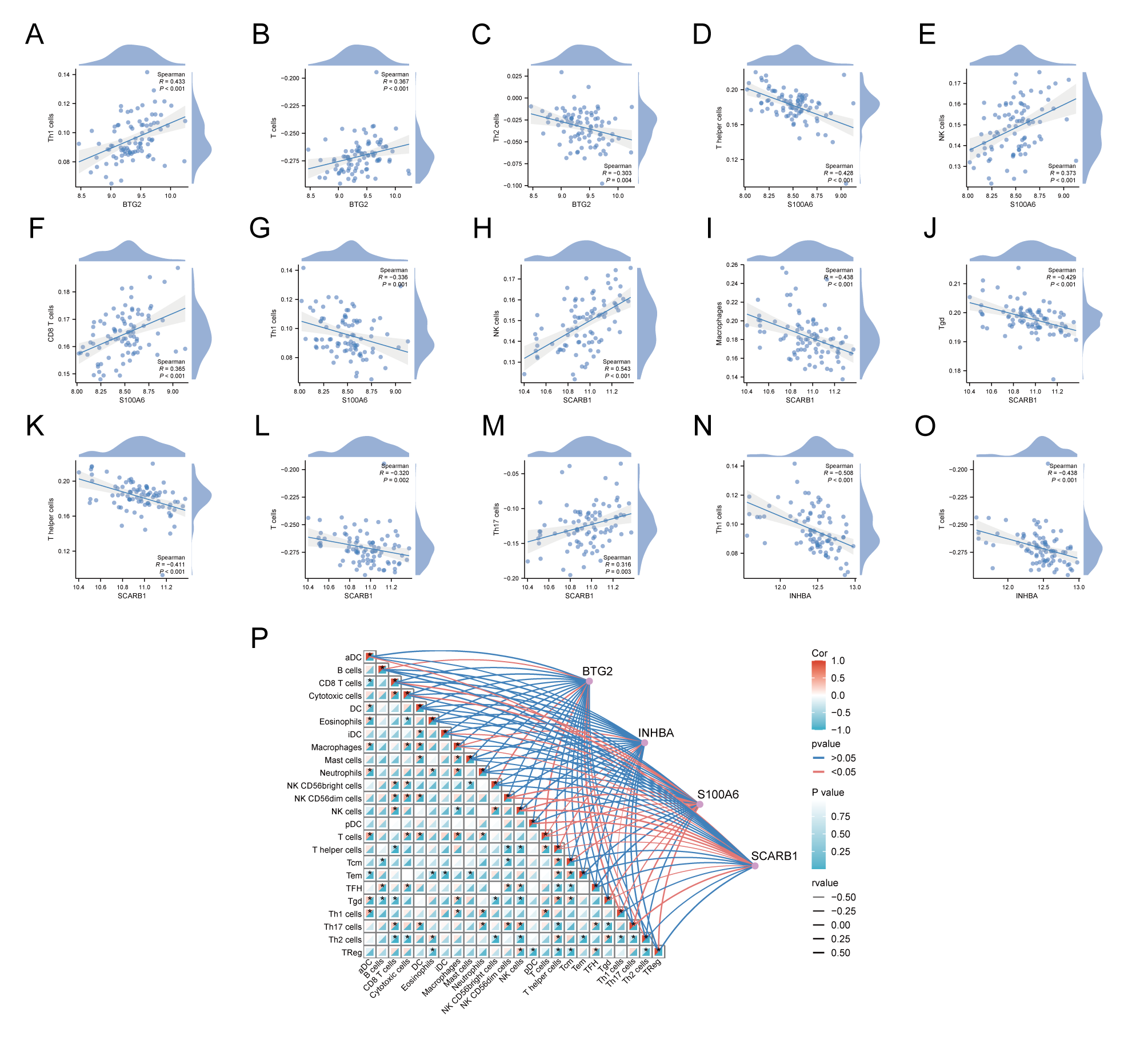

Supplement: Supplementary Figure 3 — Scatterplot with correlation network heatmap. Scatterplot showing the correlation between BTG2 (A–C), S100A6 (D–G), SCARB1 (H–M), and INHBA (N, O) and immune cells. (P) Heatmap of the correlation network of 24 immune cells. [file Image3.tif]

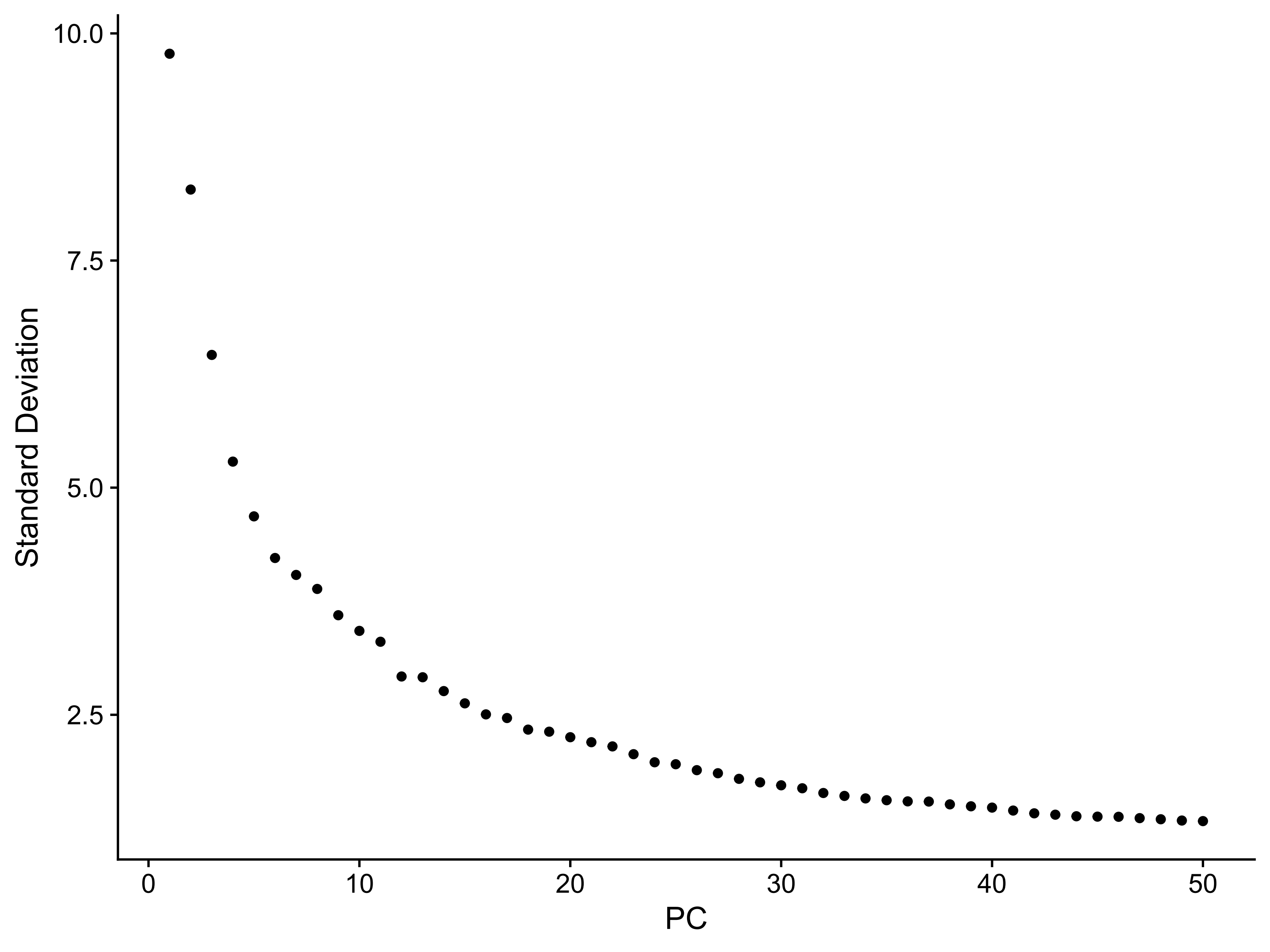

Supplement: Supplementary Figure 4 — Elbow plot for PCA dimensions selection during Harmony normalization. [file Image4.tif]

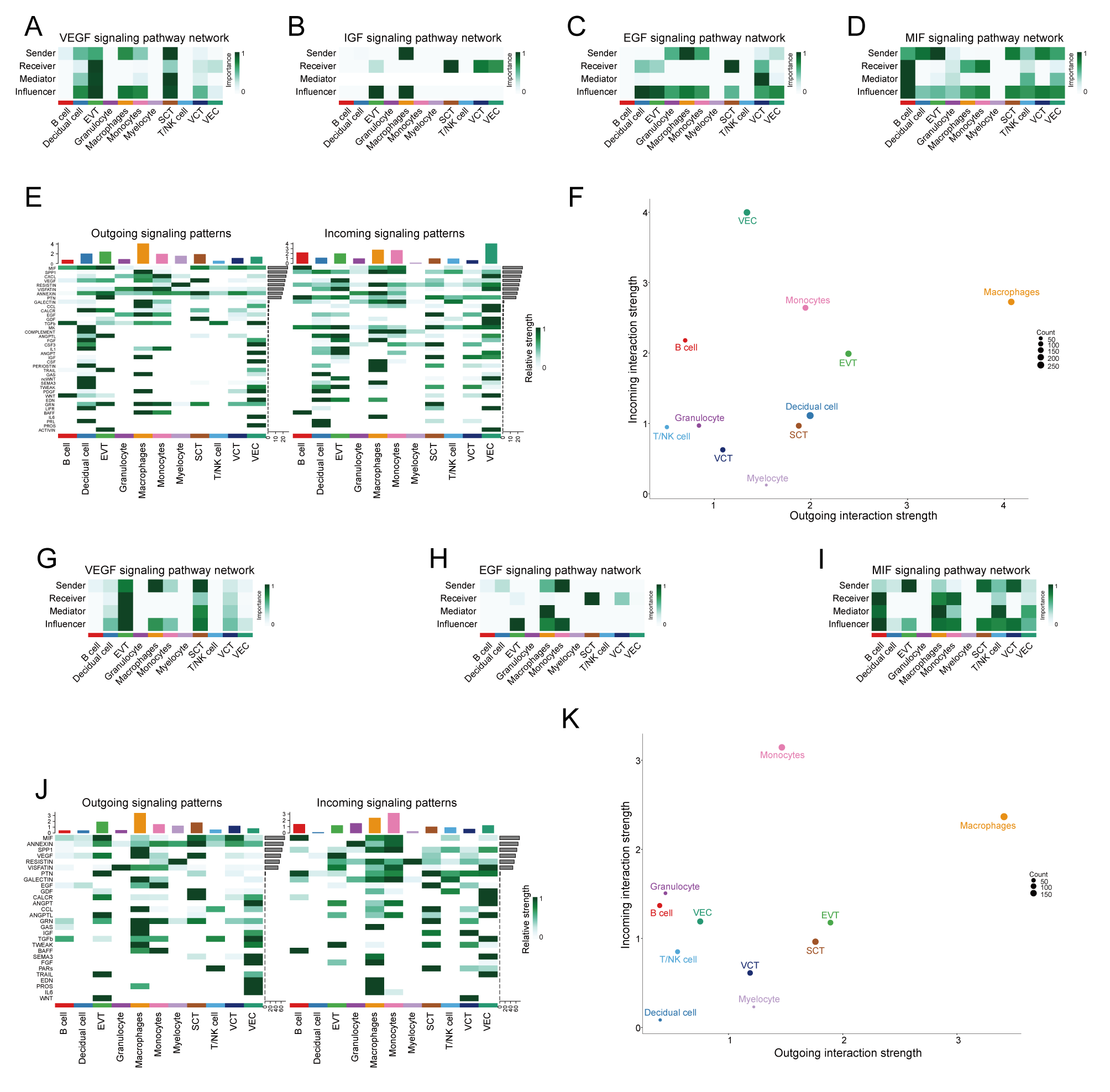

Supplement: Supplementary Figure 5 — (A–D) Network centrality analysis of four signalling pathways (VEGF, IGF, EGF and MIF) in GDM samples. (E, F) Possible roles of different cell populations in the overall communication network in GDM samples. (G–I) Network centrality analysis of three signalling pathways (VEGF, EGF and MIF) in PE samples. (J, K) Possible roles of different cell populations in the overall communication network in PE samples.GDM, gestational diabetes mellitus; PE, preeclampsia; VEGF, vascular endothelial growth factor; IGF, insulin-like growth hormone; MIF, macrophage migration inhibitory factor. [file Image5.tif]
