# Supplementary material for: Prediction and immune landscape study of potentially key autophagy-related biomarkers in preeclampsia with gestational diabetes mellitus
Source: Front Immunol. 2025 Jul 2;16:1571795. doi: 10.3389/fimmu.2025.1571795 (PMC12263617; doi:10.3389/fimmu.2025.1571795)
Supplement: Supplementary file 8 [file Table8.docx]

**#Differential Analysis**

rm(list = ls())

Sys.setenv(LANGUAGE = "en")

options(stringsAsFactors = FALSE)

getwd()

library(limma)

library(tidyverse)

library(data.table)

library(ggplot2)

library(ggrepel)

set.seed(0528)

group <- fread('Group.csv')

group <- factor(Group$group, levels = c("Control", "Disease"))

group

expr <- fread("Data_finished.csv")

expr <- as.data.frame(expr)

rownames(expr) <- expr[,1]

expr <- expr[,2:ncol(expr)]

contrast <- paste0(rev(levels(group)), collapse = "-")

design <- model.matrix( ~ 0 + group)

colnames(design) <- levels(group)

design

contrast.matrix <- makeContrasts(contrast, levels = design)

contrast.matrix

fit <- lmFit(expr, design) # expr design

fit <- contrasts.fit(fit, contrast.matrix)

fit <- eBayes(fit)

DEG <- topTable(fit, coef = 1, n = Inf) %>%

rownames_to_column(var = "symbol")

DEG_p <- DEG %>%

dplyr::filter(abs(logFC) > 0.5, P.Value < 0.05)

write.table(DEG_p, file = 'diff.txt', sep = '\t', quote = F,row.names = T, col.names = T)

group2 <- fread('Group2.csv')

group2 <- factor(Group2$group, levels = c("Control", "Disease"))

group2

expr <- fread("data_finished.csv")

expr <- as.data.frame(expr)

rownames(expr) <- expr[,1]

expr <- expr[,2:ncol(expr)]

contrast <- paste0(rev(levels(group)), collapse = "-")

design <- model.matrix( ~ 0 + group)

colnames(design) <- levels(group)

design

contrast.matrix <- makeContrasts(contrast, levels = design)

contrast.matrix

fit <- lmFit(expr, design) # expr design

fit <- contrasts.fit(fit, contrast.matrix)

fit <- eBayes(fit)

DEG <- topTable(fit, coef = 1, n = Inf) %>%

rownames_to_column(var = "symbol")

DEG_p <- DEG %>%

dplyr::filter(abs(logFC) > 0.5, P.Value < 0.05)

write.table(DEG_p, file = 'diff.txt', sep = '\t', quote = F,row.names = T, col.names = T)

#Volcano Plot

data <- fread('diff.txt')

data <- data[,-1]

logFCfilter = 0

logFCcolor = 0

colnames(data)[2] <- 'logFC'

colnames(data)[1] <- 'gene'

index = data$P.Value <0.05 & abs(data$logFC) > logFCfilter

data$group <- 0

data$group[index & data$logFC>0] = 1

data$group[index & data$logFC<0] = -1

data$group <- factor(data$group,levels = c(1,0,-1),labels =c("Up","Not sig","Down") )

ggplot(data=data, aes(x=logFC, y =-log10(P.Value),color=group)) +

geom_point(alpha=0.8, size=1.2)+

scale_color_manual(values = c("#EFC000", "grey50", "#0073C2"))+

labs(x="Log2 (fold change)",y="-log10 (P.Value)")+

theme(plot.title = element_text(hjust = 0.4))+

geom_hline(yintercept = -log10(0.05),lty=4,lwd=0.6,alpha=0.8)+

geom_vline(xintercept = c(-logFCfilter,logFCfilter),lty=4,lwd=0.6,alpha=0.8)+

theme_bw()+

theme(panel.border = element_blank(),

panel.grid.major = element_blank(),

panel.grid.minor = element_blank(),

axis.line = element_line(colour = "black")) +

theme(legend.position="top")+

geom_point(data=subset(data, abs(logFC) >= logFCcolor & P.Value <0.05),alpha=0.8, size=1,col="green4")+

geom_text_repel(data=subset(data, abs(logFC) >= logFCcolor & P.Value <0.05),

aes(label=gene),col="black",alpha = 0.8)

dev.off()

data <- fread('diff.txt')

data <- data[,-1]

logFCfilter = 0

logFCcolor = 0

colnames(data)[2] <- 'logFC'

colnames(data)[1] <- 'gene'

index = data$P.Value <0.05 & abs(data$logFC) > logFCfilter

data$group <- 0

data$group2[index & data$logFC>0] = 1

data$group2[index & data$logFC<0] = -1

data$group2 <- factor(data$group2,levels = c(1,0,-1),labels =c("Up","Not sig","Down") )

ggplot(data=data, aes(x=logFC, y =-log10(P.Value),color=group2)) +

geom_point(alpha=0.8, size=1.2)+

scale_color_manual(values = c("#EFC000", "grey50", "#0073C2"))+

labs(x="Log2 (fold change)",y="-log10 (P.Value)")+

theme(plot.title = element_text(hjust = 0.4))+

geom_hline(yintercept = -log10(0.05),lty=4,lwd=0.6,alpha=0.8)+

geom_vline(xintercept = c(-logFCfilter,logFCfilter),lty=4,lwd=0.6,alpha=0.8)+

theme_bw()+

theme(panel.border = element_blank(),

panel.grid.major = element_blank(),

panel.grid.minor = element_blank(),

axis.line = element_line(colour = "black")) +

theme(legend.position="top")+

geom_point(data=subset(data, abs(logFC) >= logFCcolor & P.Value <0.05),alpha=0.8, size=1,col="green4")+

geom_text_repel(data=subset(data, abs(logFC) >= logFCcolor & P.Value <0.05),

aes(label=gene),col="black",alpha = 0.8)

dev.off()

#Heatplot

rm(list = ls())

Sys.setenv(LANGUAGE = "en")

options(stringsAsFactors = FALSE)

getwd()

library(pheatmap)

pheat.data <- read.csv("diff.csv")

pheat.data <- pheat.data[1:40,]

rownames(pheat.data) <- pheat.data[,1]

pheat.data <- pheat.data[,-1]

Group <- read.csv("group.csv")

annotation_col = data.frame(group0 = colnames(pheat.data))

annotation_col <- annotation_col %>%

mutate(Group = case_when(

startsWith(group0, "Disease") ~ "GDM",

startsWith(group0, "Control") ~ "Control",

TRUE ~ "Other"

))

rownames(annotation_col) <- annotation_col[,1]

annotation_col <- annotation_col[,-1,drop = FALSE]

my_colors <- colorRampPalette(c("#4DBBD5", "white", "#E64B35"))(100)

annotation_colors <- list(Group = c("Control" = "#4DBBD5", "GDM" = "#E64B35"))

pheatmap(pheat.data, scale="row",

border="white",

cluster_cols = T,

cluster_rows = T,

show_rownames = T,

show_colnames = T,

legend = T,

fontsize_row = 12,

fontsize_col = 16,

legend_breaks=c(-1,0,1),

clustering_distance_rows = "euclidean",

treeheight_col = 50,

treeheight_row = 45,

annotation_col = annotation_col,

annotation_colors = annotation_colors,

color = my_colors)

pheat.data <- read.csv("diff.csv")

pheat.data <- pheat.data[1:40,]

rownames(pheat.data) <- pheat.data[,1]

pheat.data <- pheat.data[,-1]

Group <- read.csv("group.csv")

annotation_col = data.frame(group0 = colnames(pheat.data))

annotation_col <- annotation_col %>%

mutate(Group = case_when(

startsWith(group0, "Disease") ~ "PE",

startsWith(group0, "Control") ~ "Control",

TRUE ~ "Other"

))

rownames(annotation_col) <- annotation_col[,1]

annotation_col <- annotation_col[,-1,drop = FALSE]

my_colors <- colorRampPalette(c("#4DBBD5", "white", "#E64B35"))(100)

annotation_colors <- list(Group = c("Control" = "#4DBBD5", "PE" = "#E64B35"))

pheatmap(pheat.data, scale="row",

border="white",

cluster_cols = T,

cluster_rows = T,

show_rownames = T,

show_colnames = T,

legend = T,

fontsize_row = 12,

fontsize_col = 16,

legend_breaks=c(-1,0,1),

clustering_distance_rows = "euclidean",

treeheight_col = 50,

treeheight_row = 45,

annotation_col = annotation_col,

annotation_colors = annotation_colors,

color = my_colors)

**#WGCNA**

rm(list=ls())

options(stringsAsFactors = F)

setwd("")

library(WGCNA)

library(data.table)

rt=fread("data_finished.csv", data.table = F)

group <- fread("group.csv")

rownames(rt)=rt[,1]

rt=rt[,2:ncol(rt)]

rt=as.matrix(rt)

exp=rt

exp <- exp[,group$ID]

all(colnames(exp) == group$ID)

m.sd <- apply(exp, 1, sd)

exp <- exp[which(m.sd>quantile(m.sd, probs=seq(0, 1, 0.25))[4]),]

dimnames=list(rownames(exp),colnames(exp))

exp=matrix(as.numeric(as.matrix(exp)),nrow=nrow(exp),dimnames=dimnames)

dim(exp)

length(colnames(exp))

datExpr0 = as.data.frame(t(exp))

datExpr0[1:3,1:3]

gsg = goodSamplesGenes(datExpr0, verbose = 3)

gsg$allOK

sampleTree = hclust(dist(datExpr0), method = "average")

pdf(file = "01.plot1.pdf",width = 12, height = 9)

par(cex = 0.6)

par(mar = c(0,4,2,0))

# plot(sampleTree)

plot(sampleTree, main = "Sample clustering to detect outliers", sub="", xlab="", cex.lab = 1.5, cex.axis = 1.5, cex.main = 2)

abline(h = 80, col = "red")

dev.off()

clust = cutreeStatic(sampleTree, cutHeight = 80, minSize = 10)

table(clust)

keepSamples = (clust==1)

datExpr0 = datExpr0[keepSamples, ]

group$group

traitData=data.frame(Normal=c(rep(1,24),rep(0,19)),

NASH=c(rep(0,24),rep(1,19)))

dim(traitData)

row.names(traitData)=colnames(exp)

sameSample=intersect(rownames(datExpr0), rownames(traitData))

datExpr0=datExpr0[sameSample,]

datTraits=traitData[sameSample,]

sampleTree2 = hclust(dist(datExpr0), method = "average")

plot(sampleTree2)

dev.off()

traitColors = numbers2colors(datTraits, signed = FALSE)

plotDendroAndColors(sampleTree2, traitColors,

groupLabels = names(datTraits),

main = "Sample dendrogram and trait heatmap")

dev.off()

save(datExpr0, datTraits, file = "01-dataInput.RData")

rm(list = ls())

load(file = "01-dataInput.RData")

allowWGCNAThreads()

powers = c(1:20)

sft = pickSoftThreshold(datExpr0, powerVector = powers, verbose = 5)

pdf("scale_independence.pdf", width = 10, height = 8)

par(mfrow = c(1,2))

cex1 = 0.80

plot(sft$fitIndices[,1], -sign(sft$fitIndices[,3])*sft$fitIndices[,2],

xlab="Soft Threshold (power)",ylab="Scale Free Topology Model Fit,signed R^2",type="n",

main = paste("Scale independence"));

text(sft$fitIndices[,1], -sign(sft$fitIndices[,3])*sft$fitIndices[,2],

labels=powers,cex=cex1,col="red");

abline(h=0.80,col="red")

plot(sft$fitIndices[,1], sft$fitIndices[,5],

xlab="Soft Threshold (power)",ylab="Mean Connectivity", type="n",

main = paste("Mean connectivity"))

text(sft$fitIndices[,1], sft$fitIndices[,5], labels=powers, cex=cex1,col="red")

dev.off()

sft

softPower =sft$powerEstimate

adjacency = adjacency(datExpr0, power = softPower)

TOM = TOMsimilarity(adjacency);

dissTOM = 1-TOM

geneTree = hclust(as.dist(dissTOM), method = "average")

plot(geneTree, xlab="", sub="", main = "Gene clustering on TOM-based dissimilarity",

labels = FALSE, hang = 0.04)

minModuleSize = 50

dynamicMods = cutreeDynamic(dendro = geneTree, distM = dissTOM,

deepSplit = 2, pamRespectsDendro = FALSE,

minClusterSize = minModuleSize);

table(dynamicMods)

dynamicColors = labels2colors(dynamicMods)

table(dynamicColors)

pdf("Dynamic_Tree.pdf", width = 6, height = 5)

plotDendroAndColors(geneTree, dynamicColors, "Dynamic Tree Cut",

dendroLabels = FALSE, hang = 0.03,

addGuide = TRUE, guideHang = 0.05,

main = "Gene dendrogram and module colors")

dev.off()

MEList = moduleEigengenes(datExpr0, colors = dynamicColors)

MEs = MEList$eigengenes

MEDiss = 1-cor(MEs);

METree = hclust(as.dist(MEDiss), method = "average")

plot(METree, main = "Clustering of module eigengenes",

xlab = "", sub = "")

MEDissThres = 0.1

abline(h=MEDissThres, col = "red")

merge = mergeCloseModules(datExpr0, dynamicColors,

cutHeight = MEDissThres, verbose = 3)

mergedColors = merge$colors

mergedMEs = merge$newMEs

pdf("Dynamic_merged.pdf", width = 6, height = 5)

plotDendroAndColors(geneTree, mergedColors,"Merged dynamic",

dendroLabels = FALSE, hang = 0.03,

addGuide = TRUE, guideHang = 0.05,

main = "Gene dendrogram and module colors")

dev.off()

pdf("Dynamic_all.pdf", width = 6, height = 5)

plotDendroAndColors(geneTree, data.frame(dynamicColors, mergedColors),c("Dynamic Tree Cut","Merged dynamic"),

dendroLabels = FALSE, hang = 0.03,

addGuide = TRUE, guideHang = 0.05,

main = "Gene dendrogram and module colors")

dev.off()

moduleColors = mergedColors

table(moduleColors)

colorOrder = c("grey", standardColors(50))

moduleLabels = match(moduleColors, colorOrder)-1

MEs = mergedMEs

MEDiss = 1-cor(MEs);

METree = hclust(as.dist(MEDiss), method = "average")

pdf("Module tree.pdf", width = 6, height = 5)

plot(METree, main = "Clustering of module eigengenes",

xlab = "", sub = "")

dev.off()

MEs_col = MEs

library(stringr)

MEs_col = orderMEs(MEs_col)

pdf("Module_cor.pdf", width = 8, height = 8)

plotEigengeneNetworks(

MEs_col,

plotDendrograms = F,

"Eigengene adiacency heatmap",

marDendro = c(3, 3, 2, 4),

marHeatmap = c(3, 4, 2, 2),

xLabelsAngle = 90

)

dev.off()

nGenes = ncol(datExpr0)

nSamples = nrow(datExpr0)

moduleTraitCor = cor(MEs, datTraits, use = "p")

moduleTraitPvalue = corPvalueStudent(moduleTraitCor, nSamples)

textMatrix = paste(signif(moduleTraitCor, 2), "\n(",

signif(moduleTraitPvalue, 1), ")", sep = "")

dim(textMatrix) = dim(moduleTraitCor)

pdf("Module_trait.pdf", width = 6, height = 6)

par(mar = c(4, 8, 2, 1))

labeledHeatmap(Matrix = moduleTraitCor,

xLabels = names(datTraits),

yLabels = names(MEs),

ySymbols = names(MEs),

colorLabels = FALSE,

colors = blueWhiteRed(50),

textMatrix = textMatrix,

setStdMargins = FALSE,

cex.text = 0.5,

zlim = c(-1,1),

main = paste("Module-trait relationships"))

dev.off()

modNames = substring(names(MEs), 3)

geneModuleMembership = as.data.frame(cor(datExpr0, MEs, use = "p"))

MMPvalue = as.data.frame(corPvalueStudent(as.matrix(geneModuleMembership), nSamples))

names(geneModuleMembership) = paste("MM", modNames, sep="")

names(MMPvalue) = paste("p.MM", modNames, sep="")

traitNames=names(datTraits)

geneTraitSignificance = as.data.frame(cor(datExpr0, datTraits, use = "p"))

GSPvalue = as.data.frame(corPvalueStudent(as.matrix(geneTraitSignificance), nSamples))

names(geneTraitSignificance) = paste("GS.", traitNames, sep="")

names(GSPvalue) = paste("p.GS.", traitNames, sep="")

dir.create("cor_GS_MM")

for (trait in traitNames){

traitColumn=match(trait,traitNames)

for (module in modNames){

column = match(module, modNames)

moduleGenes = moduleColors==module

if (nrow(geneModuleMembership[moduleGenes,]) > 1){

outPdf=paste("cor_GS_MM/", trait, "_", module,".pdf",sep="")

pdf(file=outPdf,width=7,height=7)

par(mfrow = c(1,1))

verboseScatterplot(abs(geneModuleMembership[moduleGenes, column]),

abs(geneTraitSignificance[moduleGenes, traitColumn]),

xlab = paste("Module Membership in", module, "module"),

ylab = paste("Gene significance for ",trait),

main = paste("Module membership vs. gene significance\n"),

cex.main = 1.2, cex.lab = 1.2, cex.axis = 1.2, col = module)

abline(v=0.8,h=0.8,col="red")

dev.off()

}

}

}

dir.create("modGenes")

for (mod in 1:nrow(table(moduleColors))) {

modules = names(table(moduleColors))[mod]

probes = colnames(datExpr0)

inModule = (moduleColors == modules)

modGenes = probes[inModule]

write.table(modGenes, file =paste0("modGenes/", modules,".txt"),sep="\t",row.names=F,col.names=F,quote=F)

}

dir.create("modGenes_GS_MM")

GSgenes = rownames(geneTraitSignificance)[abs(geneTraitSignificance[,1]) > 0.8] %>%

intersect(rownames(GSPvalue)[GSPvalue[,1] < 0.05])

for (mod in 1:nrow(table(moduleColors))) {

modules = names(table(moduleColors))[mod]

probes = colnames(datExpr0)

inModule = (moduleColors == modules)

modGenes = probes[inModule]

MMgenes = rownames(geneModuleMembership)[abs(geneModuleMembership[,paste0("MM", modules)]) > 0.8] %>%

intersect(rownames(MMPvalue)[MMPvalue[,paste0("p.MM", modules)] < 0.05])

modGenes <- modGenes %>% intersect(GSgenes) %>% intersect(MMgenes)

write.table(modGenes, file =paste0("modGenes_GS_MM/", modules,".txt"),sep="\t",row.names=F,col.names=F,quote=F)

}

**#Enrichment analysis**

rm(list=ls())

options(stringsAsFactors = F)

Sys.setenv(LANGUAGE = "en")

getwd()

library(clusterProfiler)

library(org.Hs.eg.db)

library(tidyverse)

library(readxl)

library(DO.db)

library(GOplot)

library(AnnotationDbi)

gene <- read_excel("data.xlsx")

DEG <- read_excel("DEGs.xlsx")

DEG <- as.data.frame(DEG)

colnames(DEG)[1] <- "Gene"

rownames(DEG) <- DEG$Gene

abs_logfc <- 0.5

colnames(DEG)

DEG$change <- ifelse(DEG$logFC >= abs_logfc & DEG$P.Value < 0.05, "UP",

ifelse(DEG$logFC <= -abs_logfc & DEG$P.Value < 0.05, "DOWN", "NOT"))

table(DEG$change)

genelist <- bitr(DEG$Gene, fromType = "SYMBOL",

toType = "ENTREZID", OrgDb = 'org.Hs.eg.db')

DEG <- inner_join(DEG, genelist, by = c("Gene" = "SYMBOL"))

gene <- gene$`DEGs AND Autophagy`

gene <- gene[1:49]

gene_diff <- DEG[DEG$Gene %in% gene, ]

# GO

ego <- enrichGO(gene = gene_diff$ENTREZID,

OrgDb = org.Hs.eg.db,

ont = "all",

pAdjustMethod = "BH",

minGSSize = 1,

pvalueCutoff = 0.05,

qvalueCutoff = 0.05,

readable = TRUE)

write.table(ego, file = "GO.txt", sep = "\t", quote = F, row.names = F)

#KEGG

kk <- enrichKEGG(gene = gene_diff$ENTREZID,

organism = 'hsa',

keyType = "kegg",

pvalueCutoff = 0.05,

qvalueCutoff = 0.05)

write.table(kk, file = "KEGG.txt", sep = "\t", quote = F, row.names = F)

KEGG=read.table("KEGG.txt", header = T,sep="\t",check.names=F)

go=data.frame(Category = KEGG$category,ID = KEGG$ID,Term = KEGG$Description, Genes = gsub("/", ", ", KEGG$geneID), adj_pval = KEGG$p.adjust)

genelist <- go$Genes

genelist <- strsplit(genelist, ",")

genelist <- unlist(genelist)

genelist <- unique(genelist)

genelist <- DEG[match(genelist,DEG$ENTREZID),c(9,2)]

row.names(genelist)=genelist[,1]

circ <- circle_dat(go, genelist)

genename <- bitr(circ$genes, fromType = "ENTREZID",

toType = "SYMBOL", OrgDb = 'org.Hs.eg.db')

circ <- circ %>%

mutate(genes = ifelse(genes %in% genename$ENTREZID,

genename$SYMBOL[match(genes, genename$ENTREZID)],

genes))

termNum = length(unique(circ$ID))

geneNum = nrow(genelist)

keys <- keys(org.Hs.eg.db, keytype = "ENTREZID")

Type <- AnnotationDbi::select(org.Hs.eg.db, keys = keys, columns = "SYMBOL", keytype = "ENTREZID")

genelist <- genelist %>%

mutate(ID = ifelse(ID %in% Type$ENTREZID,

Type$SYMBOL[match(ID, Type$ENTREZID)],

ID))

row.names(genelist)=genelist[,1]

chord <- chord_dat(circ, genelist[1:geneNum,], go$ID[1:termNum])

pdf(file="circ.pdf",width = 11,height = 10.5)

GOChord(chord,

space = 0.001,

gene.order = 'logFC',

gene.space = 0.25,

gene.size = 4,

border.size = 0.1,

process.label = 7.5)

dev.off()

**#Machine Learning**

rm(list = ls())

Sys.setenv(LANGUAGE = "en")

options(stringsAsFactors = FALSE)

getwd()

if (!require("glmnet", quietly = TRUE)) {

install.packages("glmnet")

}

library(glmnet)

mydata <- read.csv("inpute.csv")

y <- as.matrix(mydata[, 1])

x <- as.matrix(mydata[, 2:ncol(mydata)])

alpha_values <- seq(0.01, 1, by = 0.01)

cv_errors <- numeric(length(alpha_values))

set.seed(2024)

for (i in 1:length(alpha_values)) {

cv_model <- cv.glmnet(

x, y,

family = "binomial",

alpha = alpha_values[i],

nfolds = 10

)

cv_errors[i] <- min(cv_model$cvm)

}

best_alpha <- alpha_values[which.min(cv_errors)]

lasso_model <- glmnet(

x,

y,

family = "binomial",

alpha = best_alpha

)

print(lasso_model)

cv_model <- cv.glmnet(

x, y,

family = "binomial",

alpha = best_alpha,

nfolds = 10

)

pdf("Diagnostic Coefficient.pdf", width = 8, height = 6)

plot(cv_model)

dev.off()

lambda_min <- cv_model$lambda.min

coef_cv <- coef(lasso_model, s = lambda_min)

print(coef_cv)

pdf("Variable Trajectory.pdf", width = 8, height = 6)

plot(

lasso_model,

xvar = "lambda",

label = F

)

dev.off()

coef_cv <- as.matrix(coef_cv)

coef_df <- data.frame(

variable = rownames(coef_cv),

`lambda.min` = coef_cv[, 1]

)

coef_df$OR <- exp(coef_df$`lambda.min`)

nonzero_vars <- rownames(coef_df[coef_df$lambda.min != 0, ])

write.csv(coef_df, "Lasso_Coefficients.csv", row.names = FALSE)

rm(list = ls())

Sys.setenv(LANGUAGE = "en")

options(stringsAsFactors = FALSE)

getwd()

library(caret)

library(openxlsx)

df_ml <- read.csv(file = 'HC_PE.csv') # the refining data (used for machine learning)

df_ml$group[df_ml$group == 'Control'] <- 0

df_ml$group[df_ml$group == 'PE'] <- 1

df_ml$group <- as.factor(df_ml$group)

# machine learning for selecting key genes

ml_ret <- list()

for (ml_p in c('rfFuncs', 'ldaFuncs')){

ml_ret[[ml_p]] <- rfe(x = df_ml[, 2:ncol(df_ml)], y = df_ml[, 1],

sizes = seq(1, ncol(df_ml)-1, 1),

rfeControl = rfeControl(functions = ldaFuncs, method = 'repeatedcv',

repeats = 10, number = 10,

verbose = TRUE))

}

# plotting

plot(ret[['rfFuncs']], metric = 'Accuracy', type = 'o')

plot(ret[['rfFuncs']], metric = 'Kappa', type = 'o')

plot(ret[['ldaFuncs']], metric = 'Accuracy', type = 'o')

plot(ret[['ldaFuncs']], metric = 'Kappa', type = 'o')

control <- rfeControl(functions = caret::svmFuncs,

method = 'cv',

number = 10,

verbose = TRUE)

set.seed(0528)

rfe_result <- rfe(x = df_ml[, -1],

y = df_ml[, 1],

sizes = c(1:ncol(df_ml)-1),

rfeControl = control)

**# Violin Plot**

rm(list = ls())

Sys.setenv(LANGUAGE = "en")

options(stringsAsFactors = FALSE)

getwd()

library(pacman)

library(openxlsx)

library(patchwork)

library(ggplot2)

library(circlize)

library(ggpubr)

df1 <- read.xlsx("BTG2.xlsx")

df1$`trt` <- factor(df1$`trt`,levels = c("Control","PE"))

levels(df1$`trt`) <- c("Control", "PE")

comparison <- list(c("Control","PE"))

df1$gene_name <- "BTG2"

p1 <- ggplot(data = df1,aes(x = `trt`,y = EXP, color = `trt`)) +

geom_violin(trim = F) +

geom_boxplot(width =0.45,outliers = T)+

labs(title = "",

subtitle = "") +

labs(x = "BTG2", y = "Value") +

scale_colour_manual(values = c("Control" = "#1772b4", "PE" = "#de1c31")) +

ylim(6,8)+

theme_classic()+

theme(

axis.text = element_text(color = "black", size = 12),

axis.title = element_text(color = "black", size = 12),

legend.position = "none",

legend.title = element_blank(),

panel.grid.major = element_blank(),

panel.grid.minor = element_blank(),

panel.background = element_blank()) +

stat_compare_means(comparisons = comparison,method = "wilcox",

tip.length = c(0.05,0.05),bracket.size = 0.5,

label.y = 7.5,vjust = -0.5)

p1

#######

df2 <- read.xlsx("S100A6.xlsx")

df2$`trt` <- factor(df2$`trt`,levels = c("Control","PE"))

levels(df2$`trt`) <- c("Control", "PE")

comparison <- list(c("Control","PE"))

df2$gene_name <- "S100A6"

p2 <- ggplot(data = df2,aes(x = `trt`,y = EXP, color = `trt`)) +

geom_violin(trim = F) +

geom_boxplot(width =0.45,outliers = T)+

labs(title = "",

subtitle = "") +

labs(x = "S100A6", y = "Value") +

scale_colour_manual(values = c("Control" = "#1772b4", "PE" = "#de1c31")) +

ylim(6,8)+

theme_classic()+

theme(

axis.text = element_text(color = "black", size = 12),

axis.title = element_text(color = "black", size = 12),

legend.position = "none",

legend.title = element_blank(),

panel.grid.major = element_blank(),

panel.grid.minor = element_blank(),

panel.background = element_blank()) +

stat_compare_means(comparisons = comparison,method = "wilcox",

tip.length = c(0.05,0.05),bracket.size = 0.5,

label.y = 7.5,vjust = -0.5)

p2

######

df3 <- read.xlsx("SCARB1.xlsx")

df3$`trt` <- factor(df3$`trt`,levels = c("Control","PE"))

levels(df3$`trt`) <- c("Control", "PE")

comparison <- list(c("Control","PE"))

df3$gene_name <- "SCARB1"

P3 <- ggplot(data = df3,aes(x = `trt`,y = EXP, color = `trt`)) +

geom_violin(trim = F) +

geom_boxplot(width =0.45,outliers = T)+

labs(title = "",

subtitle = "") +

labs(x = "SCARB1", y = "Value") +

scale_colour_manual(values = c("Control" = "#1772b4", "PE" = "#de1c31")) +

ylim(6,8)+

theme_classic()+

theme(

axis.text = element_text(color = "black", size = 12),

axis.title = element_text(color = "black", size = 12),

legend.position = "none",

legend.title = element_blank(),

panel.grid.major = element_blank(),

panel.grid.minor = element_blank(),

panel.background = element_blank()) +

stat_compare_means(comparisons = comparison,method = "wilcox",

tip.length = c(0.05,0.05),bracket.size = 0.5,

label.y = 7.5,vjust = -0.5)

p3

#########

df4 <- read.xlsx("INHBA.xlsx")

df$`trt` <- factor(df4$`trt`,levels = c("Control","PE"))

levels(df4$`trt`) <- c("Control", "PE")

comparison <- list(c("Control","PE"))

P4<- ggplot(data = df,aes(x = `trt`,y = EXP, color = `trt`)) +

geom_violin(trim = F) +

geom_boxplot(width =0.45,outliers = T)+

labs(title = "",

subtitle = "") +

labs(x = "INHBA", y = "Value") +

scale_colour_manual(values = c("Control" = "#1772b4", "PE" = "#de1c31")) +

ylim(6,8)+

theme_classic()+

theme(

axis.text = element_text(color = "black", size = 12),

axis.title = element_text(color = "black", size = 12),

legend.position = "none",

legend.title = element_blank(),

panel.grid.major = element_blank(),

panel.grid.minor = element_blank(),

panel.background = element_blank()) +

stat_compare_means(comparisons = comparison,method = "wilcox",

tip.length = c(0.05,0.05),bracket.size = 0.5,

label.y = 7.5,vjust = -0.5)

P4

**#ROC**

rm(list = ls())

Sys.setenv(LANGUAGE = "en")

options(stringsAsFactors = FALSE)

getwd()

library(pROC)

library(ggplot2)

library(readxl)

data <- read_excel("BTG2.xlsx")

roc <- roc(data$OUTCOME,data$Crebbp)

plot(roc,

print.auc=TRUE,print.auc.x=0.5,print.auc.y=0.5,

auc.polygon=TRUE, auc.polygon.col="skyblue",

max.auc.polygon=TRUE,

grid=c(0.1,0.2), grid.col=c("green", "red"),

print.thres=TRUE, print.thres.cex=0.8,

legacy.axes=TRUE,

main="BTG2")

data <- read_excel("S100A6.xlsx")

roc <- roc(data$OUTCOME,data$Crebbp)

plot(roc,

print.auc=TRUE,print.auc.x=0.5,print.auc.y=0.5,

auc.polygon=TRUE, auc.polygon.col="skyblue",

max.auc.polygon=TRUE,

grid=c(0.1,0.2), grid.col=c("green", "red"),

print.thres=TRUE, print.thres.cex=0.8,

legacy.axes=TRUE,

main="S100A6")

data <- read_excel("SCARB1.xlsx")

roc <- roc(data$OUTCOME,data$Crebbp);roc # Build a ROC object and compute the AUC

plot(roc,

print.auc=TRUE,print.auc.x=0.5,print.auc.y=0.5,

auc.polygon=TRUE, auc.polygon.col="skyblue",

max.auc.polygon=TRUE,

grid=c(0.1,0.2), grid.col=c("green", "red"),

print.thres=TRUE, print.thres.cex=0.8,

legacy.axes=TRUE,

main="SCARB1")

data <- read_excel("INHBA.xlsx")

roc <- roc(data$OUTCOME,data$Crebbp);roc # Build a ROC object and compute the AUC

plot(roc,

print.auc=TRUE,print.auc.x=0.5,print.auc.y=0.5,

auc.polygon=TRUE, auc.polygon.col="skyblue",

max.auc.polygon=TRUE,

grid=c(0.1,0.2), grid.col=c("green", "red"),

print.thres=TRUE, print.thres.cex=0.8,

legacy.axes=TRUE,

main="INHBA")

**#GSEA**

rm(list = ls())

Sys.setenv(LANGUAGE = "en")

options(stringsAsFactors = FALSE)

getwd()

library(clusterProfiler)

library(org.Hs.eg.db)

library(tidyverse)

library(enrichplot)

DEG <- data.table::fread("degs.csv")

colnames(DEG)[1] <- "Gene"

rownames(DEG) <- DEG$Gene

genelist <- bitr(DEG$Gene, fromType = "SYMBOL",

toType = "ENTREZID", OrgDb = 'org.Hs.eg.db')

DEG <- inner_join(DEG, genelist, by = c("Gene" = "SYMBOL"))

geneList = DEG$log2FoldChange

names(geneList) = as.character(DEG$ENTREZID)

head(geneList)

geneList = sort(geneList, decreasing = TRUE)

kegmt <- read.gmt("c2.cp.all.v2022.1.Hs.symbols.gmt")

KEGG <- GSEA(geneList, TERM2GENE = kegmt)

KEGG_result_df <- as.data.frame(KEGG)

gseaplot2(KEGG, 1, color = "red", pvalue_table = T)

gseaplot2(KEGG, 2, color = "red", pvalue_table = T)

gseaplot2(KEGG, 3, color = "red", pvalue_table = T)

gseaplot2(KEGG, 4, color = "red", pvalue_table = T)

gseaplot2(KEGG, 5, color = "red", pvalue_table = T)

**#QC and CellType**

rm(list=ls())

setwd("/home/wq030305/MY")

options(stringsAsFactors = F)

library(Seurat)

library(ggplot2)

library(clustree)

library(cowplot)

library(data.table)

library(dplyr)

library(harmony)

sce.all <- readRDS("GSE173193.rds")

mito_genes=rownames(sce.all)[grep("^MT-", rownames(sce.all),ignore.case = T)]

print(mito_genes)

#sce.all=PercentageFeatureSet(sce.all, "^MT-", col.name = "percent_mito")

sce.all=PercentageFeatureSet(sce.all, features = mito_genes, col.name = "percent_mito")

fivenum(sce.all@meta.data$percent_mito)

ribo_genes=rownames(sce.all)[grep("^Rp[sl]", rownames(sce.all),ignore.case = T)]

print(ribo_genes)

sce.all=PercentageFeatureSet(sce.all, features = ribo_genes, col.name = "percent_ribo")

fivenum(sce.all@meta.data$percent_ribo)

Hb_genes=rownames(sce.all)[grep("^Hb[^(p)]", rownames(sce.all),ignore.case = T)]

print(Hb_genes)

sce.all=PercentageFeatureSet(sce.all, features = Hb_genes,col.name = "percent_hb")

fivenum(sce.all@meta.data$percent_hb)

head(sce.all@meta.data)

feats <- c("nFeature_RNA", "nCount_RNA", "percent_mito",

"percent_ribo", "percent_hb")

feats <- c("nFeature_RNA", "nCount_RNA")

p1=VlnPlot(sce.all, group.by = "orig.ident", features = feats, pt.size = 0, ncol = 2) +

NoLegend()

p1

dev.off()

w=length(unique(sce.all$orig.ident))/3+5;w

ggsave(filename="Vlnplot1.pdf",plot=p1,width = w,height = 5)

feats <- c("percent_mito", "percent_ribo", "percent_hb")

p2=VlnPlot(sce.all, group.by = "orig.ident", features = feats, pt.size = 0, ncol = 3, same.y.lims=T) +

scale_y_continuous(breaks=seq(0, 100, 10)) +

NoLegend()

p2

dev.off()

w=length(unique(sce.all$orig.ident))/2+5;w

ggsave(filename="Vlnplot2.pdf",plot=p2,width = w,height = 5)

p3=FeatureScatter(sce.all, "nCount_RNA", "nFeature_RNA", group.by = "orig.ident", pt.size = 0.5)

p3

dev.off()

ggsave(filename="Scatterplot.pdf",plot=p3)

sce.all.filt = sce.all

selected_mito <- WhichCells(sce.all.filt, expression = percent_mito < 25)

selected_ribo <- WhichCells(sce.all.filt, expression = percent_ribo > 3)

selected_hb <- WhichCells(sce.all.filt, expression = percent_hb < 1 )

length(selected_hb)

length(selected_ribo)

length(selected_mito)

sce.all.filt <- subset(sce.all.filt, cells = selected_mito)

sce.all.filt <- subset(sce.all.filt, cells = selected_ribo)

sce.all.filt <- subset(sce.all.filt, cells = selected_hb)

dim(sce.all.filt)

table(sce.all.filt$orig.ident)

length(sce.all.filt$orig.ident)

feats <- c("nFeature_RNA", "nCount_RNA")

p1_filtered=VlnPlot(sce.all.filt, group.by = "orig.ident", features = feats, pt.size = 0, ncol = 2) +

NoLegend()

w=length(unique(sce.all.filt$orig.ident))/3+5;w

ggsave(filename="Vlnplot1_filtered.pdf",plot=p1_filtered,width = w,height = 5)

feats <- c("percent_mito", "percent_ribo", "percent_hb")

p2_filtered=VlnPlot(sce.all.filt, group.by = "orig.ident", features = feats, pt.size = 0, ncol = 3) +

NoLegend()

w=length(unique(sce.all.filt$orig.ident))/2+5;w

ggsave(filename="Vlnplot2_filtered.pdf",plot=p2_filtered,width = w,height = 5)

sce.all.filt <- NormalizeData(sce.all.filt,

normalization.method = "LogNormalize",

scale.factor = 1e4)

sce.all.filt <- FindVariableFeatures(sce.all.filt)

p4 <- VariableFeaturePlot(sce.all.filt)

p4

dev.off()

ggsave(filename="Heigh-gene.pdf",plot=p4,width = w,height = 5)

sce.all.filt <- ScaleData(sce.all.filt)

sce.all.filt <- RunPCA(sce.all.filt, features = VariableFeatures(object = sce.all.filt))

PCA_plot1=VizDimLoadings(sce.all.filt, dims = 1:2, reduction = "pca")

ggsave(filename="PCA_plot1.pdf",plot=PCA_plot1,width = 10,height = 6)

PCA_plot2=DimPlot(sce.all.filt, reduction = "pca") + NoLegend()

ggsave(filename="PCA_plot2.pdf",plot=PCA_plot2,width = 6,height = 5)

PCA_plot3=DimHeatmap(sce.all.filt, dims = 1:12, cells = 500, balanced = TRUE)

ggsave(filename="PCA_plot3.pdf",plot=PCA_plot3,width = 20,height = 20)

dev.off()

seuratObj <- RunHarmony(sce.all.filt, "orig.ident")

names(seuratObj@reductions)

seuratObj <- RunTSNE(seuratObj, dims = 1:15,

reduction = "harmony")

DimPlot(seuratObj,reduction = "tsne",label=F )

dev.off()

tsne_plot = DimPlot(seuratObj,reduction = "tsne",label=F )

ggsave(filename = "tsne_plot.pdf",plot =tsne_plot,width = 8,height = 8 )

sce.all.filt=seuratObj

sce.all.filt <- FindNeighbors(sce.all.filt, reduction = "harmony",

dims = 1:15)

sce.all.filt.all=sce.all.filt

for (res in c(0.1, 0.2, 0.3, 0.4, 0.5, 0.6,0.7,0.8,0.9,1,1.1,1.2)) {

sce.all.filt.all=FindClusters(sce.all.filt.all, #graph.name = "CCA_snn",

resolution = res, algorithm = 1)

}

colnames(sce.all.filt.all@meta.data)

apply(sce.all.filt.all@meta.data[,grep("RNA_snn",colnames(sce.all.filt.all@meta.data))],2,table)

p1_dim=plot_grid(ncol = 3, DimPlot(sce.all.filt.all, reduction = "tsne", group.by = "RNA_snn_res.0.1") +

ggtitle("louvain_0.1"), DimPlot(sce.all.filt.all, reduction = "tsne", group.by = "RNA_snn_res.0.2") +

ggtitle("louvain_0.2"), DimPlot(sce.all.filt.all, reduction = "tsne", group.by = "RNA_snn_res.0.3") +

ggtitle("louvain_0.3"))

ggsave(plot=p1_dim, filename="Dimplot_diff_resolution_low.pdf",width = 24,height = 8)

p1_dim=plot_grid(ncol = 3, DimPlot(sce.all.filt.all, reduction = "tsne", group.by = "RNA_snn_res.0.4") +

ggtitle("louvain_0.4"), DimPlot(sce.all.filt.all, reduction = "tsne", group.by = "RNA_snn_res.0.5") +

ggtitle("louvain_0.5"), DimPlot(sce.all.filt.all, reduction = "tsne", group.by = "RNA_snn_res.0.6") +

ggtitle("louvain_0.6"))

ggsave(plot=p1_dim, filename="Dimplot_diff_resolution_high.pdf",width = 24,height = 8)

p1_dim=plot_grid(ncol = 3, DimPlot(sce.all.filt.all, reduction = "tsne", group.by = "RNA_snn_res.0.7") +

ggtitle("louvain_0.7"), DimPlot(sce.all.filt.all, reduction = "tsne", group.by = "RNA_snn_res.0.8") +

ggtitle("louvain_0.8"), DimPlot(sce.all.filt.all, reduction = "tsne", group.by = "RNA_snn_res.0.9") +

ggtitle("louvain_0.9"))

ggsave(plot=p1_dim, filename="Dimplot_diff_resolution_mediul.pdf",width = 24,height = 8)

p1_dim=plot_grid(ncol = 3, DimPlot(sce.all.filt.all, reduction = "tsne", group.by = "RNA_snn_res.1") +

ggtitle("louvain_1"), DimPlot(sce.all.filt.all, reduction = "tsne", group.by = "RNA_snn_res.1.1") +

ggtitle("louvain_1.1"), DimPlot(sce.all.filt.all, reduction = "tsne", group.by = "RNA_snn_res.1.2") +

ggtitle("louvain_1.2"))

ggsave(plot=p1_dim, filename="Dimplot_diff_resolution_veryh.pdf",width = 24,height = 8)

p1_dim=plot_grid(ncol = 1, DimPlot(sce.all.filt.all, reduction = "tsne", group.by = "RNA_snn_res.0.3") +

ggtitle("louvain_0.3"))

ggsave(plot=p1_dim, filename="Dimplot_diff_resolution_0.3.pdf",width = 8,height = 8)

p2_tree=clustree(sce.all.filt.all@meta.data, prefix = "RNA_snn_res.")

ggsave(plot=p2_tree, filename="Tree_diff_resolution.pdf",width = 10,height = 10)

table(sce.all.filt.all@active.ident)

sel.clust = "RNA_snn_res.0.3"

sce.all.int <- sce.all.filt.all

sce.all.int <- SetIdent(sce.all.int, value = sel.clust)

table(sce.all.int@active.ident)

colnames(sce.all.int@meta.data)

dir.create("./3-Celltype")

setwd("./3-Celltype")

scRNA=sce.all.int

genes_to_check = c('DKK1','IGFBP1','PRL',

'HLA-G' , 'PAPPA2', #EVT

'CGA', 'CYP19A1', 'GH2', #SCT

'PARP1', #VCT

'ECM1' , 'Fibromodulin',

'CD14', 'CD52','CD83','CD86',

'AIF1', 'CD14', 'CD163',"CD209","CD53","CSF1R",

'CD3G' ,'GZMA',

"CD3D","TRBC2","GIMAP2","XCL2","GZMK","IFNG","CCL5","SAMD3",

"CD79A","CD79B","CD19","FCER2",

"CD14","CD300E","CD244","HLA-DRA","CLEC12A","FCN1",

'CD34', 'CDH5','ICAM1','PLVAP',

'CNN1', 'MYH11',

"FCGR3B","CXCL8","MNDA","SELL",

"TCN1","CEACAM8","S100A8","MMP8","DEFA4","CAMP"

)

p = DotPlot(scRNA, features = unique(genes_to_check),

assay='RNA' ) + coord_flip()

p

dev.off()

ggsave(plot=p, filename="Bubble_diagram.pdf",width = 10,height = 10)

mycolors <- c(

'#E64A35', '#4DBBD4', '#01A187', '#6BD66B', '#3C5588', '#F29F80',

'#8491B6', '#91D0C1', '#7F5F48', '#AF9E85', '#4F4FFF', '#CE3D33',

'#739B57', '#EFE685', '#446983', '#BB6239', '#5DB1DC', '#7F2268',

'#800202', '#D8D8CD', '#E6AB02', '#FFD92F', '#66C2A5'

)

tsne =DimPlot(scRNA, reduction = "tsne",cols = mycolors,pt.size = 0.8,

group.by = "RNA_snn_res.0.3",label = T,label.box = T)

tsne

dev.off()

ggsave(plot=tsne, filename="tsen_har_finished.pdf",width = 8,height = 8)

celltype=data.frame(ClusterID=0:18,

celltype= 0:18)

celltype[celltype$ClusterID %in% c( 13),2]='B cell'

celltype[celltype$ClusterID %in% c( 3,11 ),2]='EVT'

celltype[celltype$ClusterID %in% c(9),2]='SCT'

celltype[celltype$ClusterID %in% c( 7,10 ),2]='T/NK cell'

celltype[celltype$ClusterID %in% c( 0,2,5,17 ),2]='VCT'

celltype[celltype$ClusterID %in% c(8 ),2]='Monocytes'

celltype[celltype$ClusterID %in% c( 6 ),2]='Myelocyte'

celltype[celltype$ClusterID %in% c( 1,18 ),2]='Macrophages'

celltype[celltype$ClusterID %in% c( 4,12,15 ),2]='Granulocyte'

celltype[celltype$ClusterID %in% c( 14 ),2]='Decidual cell'

celltype[celltype$ClusterID %in% c( 16 ),2]='VEC'

scRNA@meta.data$celltype = "NA"

for(i in 1:nrow(celltype)){

scRNA@meta.data[which(scRNA@meta.data$RNA_snn_res.0.3 == celltype$ClusterID[i]),'celltype'] <- celltype$celltype[i]}

table(scRNA@meta.data$celltype)

th=theme(axis.text.x = element_text(angle = 45,

vjust = 0.5, hjust=0.5))

library(patchwork)

celltype_tsne =DimPlot(scRNA, reduction = "tsne",cols = mycolors,pt.size = 1,

group.by = "celltype",label = T)

celltype_tsne

dev.off()

ggsave(plot=celltype_tsne, filename="celltype_tsne.pdf",width = 8,height = 8)

sample_tsne =DimPlot(scRNA, reduction = "tsne",cols = mycolors,pt.size = 0.2,

group.by = "group")

ggsave(plot=sample_tsne, filename="sample_tsne.pdf",width = 8,height = 8)

scRNA@meta.data[["patient"]]<- factor(scRNA@meta.data[["patient"]], levels = c("GDM", "PE","Control"))

patient_tsne =DimPlot(scRNA, reduction = "tsne",cols = mycolors,pt.size = 0.2,

group.by = "patient")

ggsave(plot=patient_tsne, filename="patient_tsne.pdf",width = 8,height = 8)

#sample_patient_celltype <- sample_tsne + patient_tsne+celltype_tsne

#ggsave(plot=sample_patient_celltype, filename="sample_patient_celltype_tsen.pdf",width = 20,height = 10)

saveRDS(scRNA, "sce_celltype.rds")

getwd()

setwd("/home/wq030305/MY/3-Celltype")

library(reshape2)

library(ggplot2)

library(dplyr)

scRNA <- readRDS("/home/wq030305/MY/3-Celltype/sce_celltype.rds")

scRNA_GDM <- subset(scRNA, subset = patient == "GDM" | patient == "Control")

table(scRNA_GDM@meta.data$patient)

scRNA_GDM@meta.data$patient <- droplevels(scRNA_GDM@meta.data$patient)

unique(scRNA_GDM@meta.data$patient)

pB2_df <- table(scRNA_GDM@meta.data$celltype,scRNA_GDM@meta.data$patient) %>% melt()

colnames(pB2_df) <- c("Celltype","Sample","Number")

pB2_df$Celltype <- factor(pB2_df$Celltype)

sample_color <- c( "#d294d3",

"#f89155",

"#efe452",

"#e2c2a4",

"#88d5b9",

"#5e90b8",

"#c1a299",

"#eb652d",

"#f0c649",

"#e7e5d0",

"#658a98",

"#eb7faf",

"#94c66b",

"#f4b974",

"#00b483",

"#93a2a9")

levels(factor(pB2_df$Celltype))

pB4 <- ggplot(data = pB2_df, aes(x =Number, y = Sample, fill = Celltype)) +

geom_bar(stat = "identity", width=0.8,position="fill")+

scale_fill_manual(values=sample_color) +

theme_bw()+

theme(panel.grid =element_blank()) +

labs(x="",y="Ratio")+

theme(axis.text.y = element_text(size=12, colour = "black"))+

theme(axis.text.x = element_text(size=12, colour = "black"))+

theme(

axis.text.x.bottom = element_text(hjust = 1, vjust = 1, angle = 45)

)

pB4

dev.off()

ggsave(plot = pB4 , filename="GDM_pB4.pdf",width = 12, height = 9 )

saveRDS(scRNA_GDM,file ='scRNA_GDM.RDS')

############## PE ###########

scRNA_PE <- subset(scRNA, subset = patient == "PE" | patient == "Control")

scRNA_PE@meta.data$patient <- droplevels(scRNA_PE@meta.data$patient)

pB2_df <- table(scRNA_PE@meta.data$celltype,scRNA_PE@meta.data$patient) %>% melt()

colnames(pB2_df) <- c("Celltype","Sample","Number")

pB2_df$Celltype <- factor(pB2_df$Celltype)

sample_color <- c( "#d294d3",

"#f89155",

"#efe452",

"#e2c2a4",

"#88d5b9",

"#5e90b8",

"#c1a299",

"#eb652d",

"#f0c649",

"#e7e5d0",

"#658a98",

"#eb7faf",

"#94c66b",

"#f4b974",

"#00b483",

"#93a2a9")

levels(factor(pB2_df$Celltype))

pB4 <- ggplot(data = pB2_df, aes(x =Number, y = Sample, fill = Celltype)) +

geom_bar(stat = "identity", width=0.8,position="fill")+

scale_fill_manual(values=sample_color) +

theme_bw()+

theme(panel.grid =element_blank()) +

labs(x="",y="Ratio")+

theme(axis.text.y = element_text(size=12, colour = "black"))+

theme(axis.text.x = element_text(size=12, colour = "black"))+

theme(

axis.text.x.bottom = element_text(hjust = 1, vjust = 1, angle = 45)

)

pB4

dev.off()

ggsave(plot = pB4 , filename="PE_pB4.pdf",width = 12, height = 9 )

saveRDS(scRNA_PE,file ='scRNA_PE.RDS')

sce.all=readRDS( "/home/wq030305/MY/chapter2_new/cluster_celltype/3-Celltype/sce_celltype.rds")

dir.create("deg1")

setwd("deg1")

getwd()

r.deg=data.frame()

table(sce.all@meta.data$orig.ident)

table(sce.all@meta.data$celltype)

sce.all.GDM <- subset(sce.all, subset = group == "GDM" | group == "Control")

type=c("B cells" , "EECs" , "Endothelials" ,

"Erythrocytes" , "EVTs", "Fibroblasts" , "Granulocytes" , "Macrophages" ,

"NK cells" , "SCTs" , "SmoothMuscles" , "Stem cells" , "T cells" , "VCTs" , "VECs")

for (i in 1:length(type)) {

Idents(sce.all.GDM)="celltype"

deg=FindMarkers(sce.all.GDM,ident.1 = "GDM",ident.2 = "Control",

group.by = "group",subset.ident =type[i] )

write.csv(deg,file = paste0( type[i],'deg.csv') )

deg$gene=rownames(deg)

deg$celltype=type[i]

deg$unm=i-1

r.deg=rbind(deg,r.deg)

}

table(r.deg$unm)

r.deg <- subset(r.deg, p_val < 0.05 & abs(avg_log2FC) > 0)

r.deg$threshold <- as.factor(ifelse(r.deg$avg_log2FC > 0 , 'Up', 'Down'))

dim(r.deg)

r.deg$p_val_signi <- as.factor(ifelse(r.deg$p_val < 0.01 , 'Highly', 'Lowly'))

r.deg$thr_signi <- paste0(r.deg$threshold, "_", r.deg$p_val_signi)

r.deg$unm %<>% as.vector(.) %>% as.numeric(.)

top_up_label <- r.deg %>%

subset(., threshold%in%"Up") %>%

group_by(unm) %>%

top_n(n = 5, wt = p_val) %>%

as.data.frame()

top_down_label <- r.deg %>%

subset(., threshold %in% "Down") %>%

group_by(unm) %>%

top_n(n = -5, wt = p_val) %>%

as.data.frame()

top_label <- rbind(top_up_label,top_down_label)

top_label$thr_signi %<>%

factor(., levels = c("Up_Highly","Down_Highly","Up_Lowly","Down_Lowly"))

target_label <- subset(r.deg, gene %in% c("BTG2", "S100A6", "SCARB1", "INHBA"))

rownames(target_label) <- NULL

write.csv(target_label,"target_label.csv",quote = F)

colnames(r.deg)

background_position <- r.deg %>%

dplyr::group_by(unm) %>%

dplyr::summarise(Min = min(p_val) - 0.2, Max = max(p_val) + 0.2) %>%

as.data.frame()

## `summarise()` ungrouping output (override with `.groups` argument)

background_position$unm %<>% as.vector(.) %>% as.numeric(.)

background_position$start <- background_position$unm - 0.4

background_position$end <- background_position$unm + 0.4

cluster_bar_position <- background_position

cluster_bar_position$start <- cluster_bar_position$unm - 0.5

cluster_bar_position$end <- cluster_bar_position$unm + 0.5

cluster_bar_position$unm %<>%

factor(., levels = c(0:max(as.vector(.))))

cols_thr_signi <- c("Up_Highly" = "#e64b35",

"Down_Highly" = "#4dbbd5",

"Up_Lowly" = "#d3d3d3",

"Down_Lowly" = "#d3d3d3")

cols_cluster <- c("0" = "#d294d3",

"1" = "#8dd3c7",

"2" = "#ffffb3",

"3" = "#bebada",

"4" = "#fb8072",

"5" = "#80b1d3",

"6" = "#fdb462",

"7" = "#b3de69",

"8" = "#CE3D33",

"9" = "#66c2a5",

"10" = "#FFD92F",

"11" = "#4f4fff")

p= ggplot() +

geom_rect(data = background_position, aes(xmin = start, xmax = end, ymin = Min,

ymax = Max),

fill = "#525252", alpha = 0.1) +

geom_jitter(data = r.deg, aes(x =unm, y = avg_log2FC, colour = thr_signi),

size = 1,position = position_jitter(seed = 1)) +

scale_color_manual(values = cols_thr_signi) +

scale_x_continuous(limits = c(-0.5, max(r.deg$unm) + 0.5),

breaks = seq(0, max(r.deg$unm), 1),

label = c("B cells","EECs","Endothelials","Erythrocytes","EVTs","Fibroblasts","Granulocytes","Macrophages","NK cells","SmoothMuscles","Stem cells","SCTs","T cells","VECs","VCTs"))+

#label = seq(0, max(r.deg$unm),1)) +

geom_text_repel(data = target_label, aes(x =unm, y = avg_log2FC, label = gene),

position = position_jitter(seed = 1), show.legend = F, size = 2.5,

box.padding = unit(0, "lines")) +

labs(x = "Cluster", y = "average log2FC") +

theme_bw()

plot1 <- p + theme(panel.grid.minor = element_blank(),

panel.grid.major = element_blank(),

axis.text.x = element_text(angle = 45, hjust = 1))

plot1

dev.off()

ggsave(filename = "GDM_deg_pointplot.pdf", plot = plot1, width = 12, height = 9)

rm(list = ls())

setwd("/home/wq030305/MY")

dir.create("chapter3_new_DEGs")

setwd("./chapter3_new_DEGs")

getwd()

sce.all=readRDS( "/home/wq030305/MY/chapter2_new/cluster_celltype/3-Celltype/sce_celltype.rds")

dir.create("deg2")

setwd("deg2")

getwd()

r.deg=data.frame()

table(sce.all@meta.data$orig.ident)

table(sce.all@meta.data$celltype)

sce.all.PE <- subset(sce.all, subset = group == "PE" | group == "Control")

type=c("B cells" , "EECs" , "Endothelials" ,

"Erythrocytes" , "EVTs", "Fibroblasts" , "Granulocytes" , "Macrophages" ,

"NK cells" , "SCTs" , "SmoothMuscles" , "Stem cells" , "T cells" , "VCTs" , "VECs")

for (i in 1:length(type)) {

Idents(sce.all)="celltype"

deg=FindMarkers(sce.all,ident.1 = "PE",ident.2 = "Control",

group.by = "group",subset.ident =type[i] )

write.csv(deg,file = paste0( type[i],'deg.csv') )

deg$gene=rownames(deg)

deg$celltype=type[i]

deg$unm=i-1

r.deg=rbind(deg,r.deg)

}

table(r.deg$unm)

r.deg <- subset(r.deg, p_val < 0.05 & abs(avg_log2FC) > 0)

r.deg$threshold <- as.factor(ifelse(r.deg$avg_log2FC > 0 , 'Up', 'Down'))

dim(r.deg)

r.deg$p_val_signi <- as.factor(ifelse(r.deg$p_val < 0.01 , 'Highly', 'Lowly'))

r.deg$thr_signi <- paste0(r.deg$threshold, "_", r.deg$p_val_signi)

r.deg$unm %<>% as.vector(.) %>% as.numeric(.)

top_up_label <- r.deg %>%

subset(., threshold%in%"Up") %>%

group_by(unm) %>%

top_n(n = 5, wt = p_val) %>%

as.data.frame()

top_down_label <- r.deg %>%

subset(., threshold %in% "Down") %>%

group_by(unm) %>%

top_n(n = -5, wt = p_val) %>%

as.data.frame()

top_label <- rbind(top_up_label,top_down_label)

top_label$thr_signi %<>%

factor(., levels = c("Up_Highly","Down_Highly","Up_Lowly","Down_Lowly"))

target_label <- subset(r.deg, gene %in% c("BTG2", "S100A6", "SCARB1", "INHBA"))

rownames(target_label) <- NULL

write.csv(target_label,"target_label_PE.csv",quote = F)

colnames(r.deg)

background_position <- r.deg %>%

dplyr::group_by(unm) %>%

dplyr::summarise(Min = min(p_val) - 0.2, Max = max(p_val) + 0.2) %>%

as.data.frame()

## `summarise()` ungrouping output (override with `.groups` argument)

background_position$unm %<>% as.vector(.) %>% as.numeric(.)

background_position$start <- background_position$unm - 0.4

background_position$end <- background_position$unm + 0.4

cluster_bar_position <- background_position

cluster_bar_position$start <- cluster_bar_position$unm - 0.5

cluster_bar_position$end <- cluster_bar_position$unm + 0.5

cluster_bar_position$unm %<>%

factor(., levels = c(0:max(as.vector(.))))

cols_thr_signi <- c("Up_Highly" = "#e64b35",

"Down_Highly" = "#4dbbd5",

"Up_Lowly" = "#d3d3d3",

"Down_Lowly" = "#d3d3d3")

cols_cluster <- c("0" = "#d294d3",

"1" = "#f89155",

"2" = "#efe452",

"3" = "#e2c2a4",

"4" = "#88d5b9",

"5" = "#5e90b8",

"6" = "#c1a299",

"7" = "#eb652d",

"8" = "#f0c649",

"9" = "#e7e5d0",

"10"="#658a98",

"11"="#eb7faf",

"12"="#94c66b",

"13"="#f4b974",

"14"="#00b483",

"15"="#93a2a9")

p= ggplot() +

geom_rect(data = background_position, aes(xmin = start, xmax = end, ymin = Min,

ymax = Max),

fill = "#525252", alpha = 0.1) +

geom_jitter(data = r.deg, aes(x =unm, y = avg_log2FC, colour = thr_signi),

size = 1,position = position_jitter(seed = 1)) +

scale_color_manual(values = cols_thr_signi) +

scale_x_continuous(limits = c(-0.5, max(r.deg$unm) + 0.5),

breaks = seq(0, max(r.deg$unm), 1),

label = c("B cells","EECs","Endothelials","Erythrocytes","EVTs","Fibroblasts","Granulocytes","Macrophages","NK cells","SmoothMuscles","Stem cells","SCTs","T cells","VECs","VCTs"))+

#label = seq(0, max(r.deg$unm),1)) +

geom_text_repel(data = target_label, aes(x =unm, y = avg_log2FC, label = gene),

position = position_jitter(seed = 1), show.legend = F, size = 2.5,

box.padding = unit(0, "lines")) +

labs(x = "Cluster", y = "average log2FC") +

theme_bw()

plot1 <- p + theme(panel.grid.minor = element_blank(),

panel.grid.major = element_blank(),

axis.text.x = element_text(angle = 45, hjust = 1))

plot1

dev.off()

ggsave(filename = "deg_pointplot_PE.pdf", plot = plot1, width = 12, height = 9)

#**cellchat**

rm(list=ls())

Sys.setenv(LANGUAGE="en")

setwd("/home/wq030305/MY")

options(stringsAsFactors = F)

library(Seurat)

library(harmony)

library(reshape2)

library(ggplot2)

library(RColorBrewer)

library(CellChat)

library(patchwork)

library(ggalluvial)

library(NMF)

library(ggsci)

dir.create("./chapter5_new_cellchat")

setwd("./chapter5_new_cellchat")

getwd()

dir.create("./PE")

setwd("./PE")

getwd()

scRNAsub=readRDS('/home/wq030305/MY/chapter4_new_recelltype/PE_scRNA_VCTs.RDS')

scRNA=readRDS('/home/wq030305/MY/chapter2_new/cluster_celltype/3-Celltype/scRNA_PE.RDS')

scRNAsub$celltype=ifelse(scRNAsub$seurat_clusters %in% c(2,4,6,7),'PE_VCTs','Other_VCTs')

table(scRNA@meta.data$celltype)

scRNAother=subset(scRNA, celltype != 'VCTs')

set.seed(0528)

a=sample(1:ncol(scRNAother),6000)

scRNAother=scRNAother[,a]

scRNA_chat=merge(scRNAsub,c(scRNAother))

scRNA_chat <- subset(scRNA_chat, group=='PE')

meta =scRNA_chat@meta.data # a dataframe with rownames containing cell mata data

scRNA_chat[["RNA"]]$data

# Alternate accessor function with the same result

LayerData(scRNA_chat, assay = "RNA", layer = "data")

scRNA_chat

scRNA_chat <- JoinLayers(scRNA_chat)

scRNA_chat

data_input <- as.matrix(scRNA_chat@assays$RNA$data)

identical(colnames(data_input),rownames(meta))

cellchat <- createCellChat(object = data_input, meta = meta, group.by = "celltype")

CellChatDB <- CellChatDB.human

groupSize <- as.numeric(table(cellchat@idents))

CellChatDB.use <- subsetDB(CellChatDB, search = "Secreted Signaling")

cellchat@DB <- CellChatDB.use

dplyr::glimpse(CellChatDB$interaction)

cellchat <- subsetData(cellchat)

cellchat <- identifyOverExpressedGenes(cellchat)

cellchat <- identifyOverExpressedInteractions(cellchat)

cellchat <- projectData(cellchat, PPI.human)

unique(cellchat@idents)

cellchat <- computeCommunProb(cellchat,raw.use = TRUE)

# Filter out the cell-cell communication if there are only few number of cells in certain cell groups

cellchat <- filterCommunication(cellchat, min.cells = 3)

cellchat <- computeCommunProbPathway(cellchat)

cellchat <- aggregateNet(cellchat)

df.net.1 <- subsetCommunication(cellchat,slot.name = "netP")

write.csv(df.net.1,"df.net.1.csv",row.names = F)

df.net.2 <- subsetCommunication(cellchat )

write.csv(df.net.2,"df.net.2.csv",row.names = F)

table(df.net.1$pathway_name)

table(df.net.2$pathway_name)

groupSize <- as.numeric(table(cellchat@idents))

par(mfrow = c(1,2), xpd=TRUE)

netVisual_circle(cellchat@net$count, vertex.weight = groupSize, weight.scale = T, label.edge= F, title.name = "Number of interactions")

netVisual_circle(cellchat@net$weight, vertex.weight = groupSize, weight.scale = T, label.edge= F, title.name = "Interaction weights/strength")

p_bubble= netVisual_bubble(cellchat,

targets.use = c('Other_VCTs','PE_VCTs'),

remove.isolate = FALSE)+coord_flip()

p_bubble

dev.off()

ggsave(plot = p_bubble , filename = "two_VCTs_target.pdf",width = 12,height = 9)

p_bubble= netVisual_bubble(cellchat,

sources.use = c('Other_VCTs','PE_VCTs'),

remove.isolate = FALSE)+coord_flip()

p_bubble

dev.off()

ggsave(plot = p_bubble , filename = "two_VCTs_source.pdf",width = 12,height = 9)

df.net_source <- subsetCommunication(cellchat, sources.use = c(10,11), targets.use = c(1,2,3,4,5,6,7,8,9,12,13,14,15,16))

df.net_target <- subsetCommunication(cellchat, sources.use = c(1,2,3,4,5,6,7,8,9,12,13,14,15,16), targets.use = c(10,11))

df.net_source

df.net_target

table(df.net_source$pathway_name)

table(df.net_target$pathway_name)

df.net_VCTs_VCTs <- subsetCommunication(cellchat, sources.use = c(10,11), targets.use = c(10,11))

table(df.net_VCTs_VCTs$pathway_name)

source <- unique(df.net_source$pathway_name)

target <- unique(df.net_target$pathway_name)

VCTs_VCTs <- unique(df.net_VCTs_VCTs$pathway_name)

all_PE_VCTs_relate_pathway <- unique(c(source, target, VCTs_VCTs))

df <- data.frame(pathway = all_PE_VCTs_relate_pathway)

write.csv(df, file = "all_PE_VCTs_relate_pathway.csv", row.names = FALSE)

saveRDS(cellchat,file ='cellchat.RDS')

setwd("/home/wq030305/MY/chapter5_new_cellchat/PE")

dir.create("river")

setwd("river")

getwd()

cellchat=readRDS('/home/wq030305/MY/chapter5_new_cellchat/PE/cellchat.RDS')

library(NMF)

library(ggalluvial)

selectK(cellchat, pattern = "outgoing")

nPatterns = 3

cellchat <- identifyCommunicationPatterns(cellchat, pattern = "outgoing", k = nPatterns)

##river plot

netAnalysis_river(cellchat, pattern = "outgoing")

netAnalysis_dot(cellchat, pattern = "outgoing")

selectK(cellchat, pattern = "incoming")

nPatterns = 3

cellchat <- identifyCommunicationPatterns(cellchat, pattern = "incoming", k = nPatterns)

netAnalysis_river(cellchat, pattern = "incoming")

netAnalysis_dot(cellchat, pattern = "incoming")

dir.create("./hierarchy_plot")

setwd("./hierarchy_plot")

getwd()

pdf("hierarchy_plot_MK.pdf", width = 12, height = 8)

vertex.receiver = seq(5,9) # a numeric vector.

netVisual_aggregate(cellchat, signaling = "CCL",

vertex.receiver = c(10,11),layout="hierarchy")

dev.off()

par(mfrow=c(1,1))

netVisual_aggregate(cellchat, signaling ="MK", layout = "circle",signaling.name="MK Signaling Pathway")

pdf("chord_target_MK.pdf", width = 6, height = 6)

netVisual_aggregate(cellchat,

signaling ="MK",

targets.use = c(5,9),

sources.use = c(1,2,3,4,6,7,8,10,11),

layout = "chord",

vertex.size = groupSize)

dev.off()

pdf("chord_source_MK.pdf", width = 6, height = 6)

netVisual_aggregate(cellchat,

signaling ="MK",

sources.use = c(5,9),

targets.use = c(1,2,3,4,6,7,8,10,11),

layout = "chord",

vertex.size = groupSize)

dev.off()

netAnalysis_contribution(cellchat, signaling = "MK")

netAnalysis_contribution(cellchat, signaling = c("MK","MIF"))

pathways.show <- "MIF"

netAnalysis_contribution(cellchat, signaling = pathways.show)

pairLR.MK <- extractEnrichedLR(cellchat, signaling = "MK", geneLR.return = F)

LR.show <- pairLR.MK[6,] # show one ligand-receptor pair

netVisual_individual(cellchat,

signaling ="MK" ,

pairLR.use = LR.show,

vertex.receiver = vertex.receiver,

layout="hierarchy")

netVisual_individual(cellchat, signaling =pathways.show , pairLR.use = LR.show, vertex.receiver = c(5,9),layout="hierarchy")

plotGeneExpression(cellchat, signaling = "MK")

setwd("/home/wq030305/liver-bufenceng/cell_chat_5")

cellchat <- netAnalysis_computeCentrality(cellchat, slot.name = "netP")

pdf("signaling patterns.pdf", width = 12, height = 8)

h1=netAnalysis_signalingRole_heatmap(cellchat, pattern = "outgoing")

h2=netAnalysis_signalingRole_heatmap(cellchat, pattern = "incoming")

h1 + h2

dev.off()

gg1 <- netAnalysis_signalingRole_scatter(cellchat)

gg1

dev.off()

ggsave(plot = gg1 , filename = "gg1.pdf",height = 8,width = 12)

library(NMF)

library(ggalluvial)

selectK(cellchat, pattern = "outgoing")

nPatterns = 5

cellchat <- identifyCommunicationPatterns(cellchat, pattern = "outgoing", k = nPatterns)

netAnalysis_river(cellchat, pattern = "outgoing")

netAnalysis_dot(cellchat, pattern = "outgoing")

selectK(cellchat, pattern = "incoming")

nPatterns = 4

cellchat <- identifyCommunicationPatterns(cellchat, pattern = "incoming", k = nPatterns)

netAnalysis_river(cellchat, pattern = "incoming")

netAnalysis_dot(cellchat, pattern = "incoming")

mat <- cellchat@net$weight

par(mfrow = c(3,3), xpd=TRUE)

for (i in 1:nrow(mat)) {

mat2 <- matrix(0, nrow = nrow(mat), ncol = ncol(mat), dimnames = dimnames(mat))

mat2[i, ] <- mat[i, ]

netVisual_circle(mat2, vertex.weight = groupSize, weight.scale = T, edge.weight.max = max(mat), title.name = rownames(mat)[i])

}

mat <- cellchat@net$count

par(mfrow = c(3,3), xpd=TRUE)

for (i in 1:nrow(mat)) {

mat2 <- matrix(0, nrow = nrow(mat), ncol = ncol(mat), dimnames = dimnames(mat))

mat2[i, ] <- mat[i, ]

netVisual_circle(mat2, vertex.weight = groupSize, weight.scale = T, edge.weight.max = max(mat), title.name = rownames(mat)[i])

}

df.net <- subsetCommunication(cellchat, sources.use = c(1,2), targets.use = c(4,5))

df.net <- subsetCommunication(cellchat, signaling = c("WNT", "TGFb"))

groupSize <- as.numeric(table(cellchat@idents))

par(mfrow = c(1,2), xpd=TRUE)

netVisual_circle(cellchat@net$count, vertex.weight = groupSize, weight.scale = T, label.edge= F, title.name = "Number of interactions")

netVisual_circle(cellchat@net$weight, vertex.weight = groupSize, weight.scale = T, label.edge= F, title.name = "Interaction weights/strength")

mat <- cellchat@net$weight

par(mfrow = c(4,4), xpd=TRUE)

for (i in 1:nrow(mat)) {

mat2 <- matrix(0, nrow = nrow(mat), ncol = ncol(mat), dimnames = dimnames(mat))

mat2[i, ] <- mat[i, ]

netVisual_circle(mat2, vertex.weight = groupSize, weight.scale = T, edge.weight.max = max(mat), title.name = rownames(mat)[i])

}

mat <- cellchat@net$count

par(mfrow = c(3,3), xpd=TRUE)

for (i in 1:nrow(mat)) {

mat2 <- matrix(0, nrow = nrow(mat), ncol = ncol(mat), dimnames = dimnames(mat))

mat2[i, ] <- mat[i, ]

netVisual_circle(mat2, vertex.weight = groupSize, weight.scale = T, edge.weight.max = max(mat), title.name = rownames(mat)[i])

}

**#Proposed temporal trajectory analysis**

rm(list=ls())

options(stringsAsFactors = F)

Sys.setenv("LANGUAGE"="en")

setwd("/home/wq030305/MY/7_monocle2")

library(tidyverse)

library(tinyarray)

library(data.table)

library(Seurat)

library(ggplot2)

library(clustree)

library(cowplot)

library(dplyr)

library(monocle)

scRNAsub=readRDS('/home/wq030305/MY/6_smallcelltype/scRNA_EVT_GDM.RDS')

scRNA=readRDS('/home/wq030305/MY/3-Celltype/scRNA_GDM.RDS')

table(scRNAsub@meta.data$seurat_clusters)

scRNAsub$celltype=ifelse(scRNAsub$seurat_clusters %in% c(1,6,10),'GDM_EVT','Other_EVT')

scRNAsub_1610=subset(scRNAsub,celltype %in% 'GDM_EVT')

table(Idents(scRNAsub_1610))

sce = scRNAsub_1610

sce@meta.data$seurat_clusters <- droplevels(sce@meta.data$seurat_clusters)

allCells=names(Idents(sce))

allType = levels(Idents(sce))

cg_sce = sce

table(Idents(cg_sce))

table(sce@meta.data$seurat_clusters)

Mono_tj<-cg_sce

Mono_matrix<-as(as.matrix(GetAssayData(Mono_tj,layer = "counts")), 'sparseMatrix')

feature_ann<-data.frame(gene_id=rownames(Mono_matrix),gene_short_name=rownames(Mono_matrix))

rownames(feature_ann)<-rownames(Mono_matrix)

Mono_fd<-new("AnnotatedDataFrame", data = feature_ann)

sample_ann<-Mono_tj@meta.data

rownames(sample_ann)<-colnames(Mono_matrix)

Mono_pd<-new("AnnotatedDataFrame", data =sample_ann)

Mono.cds<-newCellDataSet(Mono_matrix,phenoData =Mono_pd,featureData =Mono_fd,expressionFamily=negbinomial.size())

head(pData(Mono.cds))

head(fData(Mono.cds))

Mono.cds <- estimateSizeFactors(Mono.cds)

Mono.cds <- estimateDispersions(Mono.cds)

disp_table <- dispersionTable(Mono.cds)

unsup_clustering_genes <- subset(disp_table, mean_expression >= 0.1)

Mono.cds <- setOrderingFilter(Mono.cds, unsup_clustering_genes$gene_id)

Mono.cds <- reduceDimension(

Mono.cds,

max_components = 2,

method = 'DDRTree')

Mono.cds <- orderCells(Mono.cds)

head(pData(Mono.cds))

pdf("plot_cell_trajectory1.pdf", width = 12, height = 9)

plot_cell_trajectory(Mono.cds,cell_size = 1)

dev.off()

pdf("plot_cell_trajectory2-1.pdf", width = 12, height = 9)

plot_cell_trajectory(Mono.cds,color_by="seurat_clusters", size=1,show_backbone=TRUE)

dev.off()

pdf("plot_cell_trajectory2-2.pdf", width = 12, height = 9)

plot_cell_trajectory(Mono.cds,color_by="Pseudotime", size=1,show_backbone=TRUE)

dev.off()

pdf("plot_cell_trajectory3.pdf", width = 24, height = 9)

plot_cell_trajectory(Mono.cds,cell_size = 1 ) + facet_wrap("~patient", nrow = 1)

dev.off()

library(RColorBrewer)

mycolors <- brewer.pal(11, "Set3")

plot_complex_cell_trajectory(Mono.cds,x=1,y=2,color_by="seurat_clusters")+

scale_color_manual(values =mycolors)+

theme(legend.title = element_blank())

library(ggpubr)

df <- pData(Mono.cds)

view(df)

pdf("monocle4.pdf", width = 12, height = 9)

ggplot(df,aes(Pseudotime, colour = seurat_clusters, fill=seurat_clusters))+

geom_density(bw=0.5,size=1,alpha =0.5)+theme_classic2()

dev.off()

head(unsup_clustering_genes)

write.csv(unsup_clustering_genes,"unsup_clustering_genes.csv",row.names = F)

s.genes <- c("BTG2","S100A6","SCARB1","INHBA")

p1 <- plot_genes_jitter(Mono.cds[s.genes,],

grouping = "State", color_by = "State")

p2 <- plot_genes_violin(Mono.cds[s.genes,],

grouping = "State", color_by = "State")

p3 <- plot_genes_in_pseudotime(Mono.cds[s.genes,], color_by = "State")

pdf("p1.pdf", width = 12, height = 9)

p1

dev.off()

pdf("p2.pdf", width = 12, height = 9)

p2

dev.off()

pdf("p3.pdf", width = 12, height = 9)

p3

dev.off()

disp.genes <- subset(disp_table, mean_expression >= 1&dispersion_empirical >= 3*dispersion_fit)

disp.genes <- as.character(disp.genes$gene_id)

diff_test <- differentialGeneTest(Mono.cds[disp.genes,], cores = 4,

fullModelFormulaStr = "~sm.ns(Pseudotime)")

sig_gene_names <- row.names(subset(diff_test, qval < 1e-50))

pdf("heatplot1.pdf", width = 12, height = 9)

plot_pseudotime_heatmap(Mono.cds[sig_gene_names,], num_clusters=4,

show_rownames=T, return_heatmap=T)

dev.off()

saveRDS(Mono.cds,file ='Mono.cds.RDS')

Mono.cds=readRDS('/home/wq030305/MY/7_monocle2/Mono.cds.RDS')
